# Inclusive Pattern Generation Protocols to Decode Thiol-Mediated Uptake

**DOI:** 10.1021/acscentsci.3c01601

**Published:** 2024-04-17

**Authors:** Saidbakhrom Saidjalolov, Filipe Coelho, Vincent Mercier, Dimitri Moreau, Stefan Matile

**Affiliations:** § Department of Organic Chemistry, University of Geneva, CH-1211 Geneva, Switzerland; † Department of Biochemistry, University of Geneva, CH-1211 Geneva, Switzerland

## Abstract

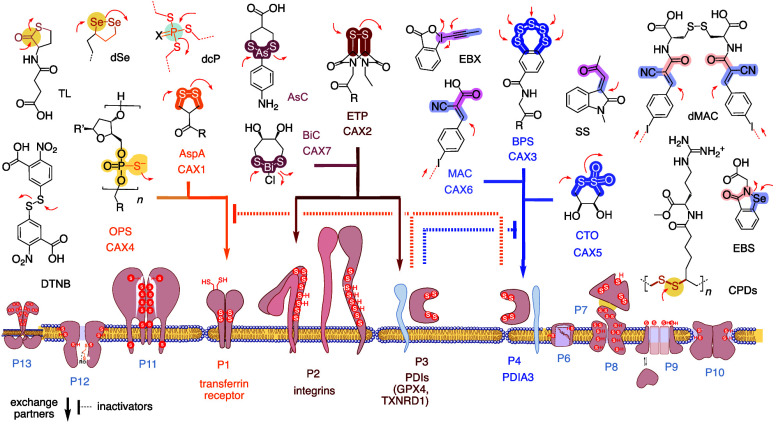

Thiol-mediated uptake (TMU) is an intriguing enigma in current chemistry and biology. While the appearance of cell-penetrating activity upon attachment of cascade exchangers (CAXs) has been observed by many and is increasingly being used in practice, the molecular basis of TMU is essentially unknown. The objective of this study was to develop a general protocol to decode the dynamic covalent networks that presumably account for TMU. Uptake inhibition patterns obtained from the removal of exchange partners by either protein knockdown or alternative inhibitors are aligned with original patterns generated by CAX transporters and inhibitors and patterns from alternative functions (here cell motility). These inclusive TMU patterns reveal that the four most significant CAXs known today enter cells along three almost orthogonal pathways. Epidithiodiketopiperazines (ETP) exchange preferably with integrins and protein disulfide isomerases (PDIs), benzopolysulfanes (BPS) with different PDIs, presumably PDIA3, and asparagusic acid (AspA), and antisense oligonucleotide phosphorothioates (OPS) exchange with the transferrin receptor and can be activated by the removal of PDIs with their respective inhibitors. These findings provide a solid basis to understand and use TMU to enable and prevent entry into cells.

## Abstract 2

Methods are developed to show that different, almost orthogonal dynamic covalent networks operate in thiol-mediated uptake to deliver substrates of interest into cells and to reveal which ones are involved.

## Introduction

**Figure 1:**
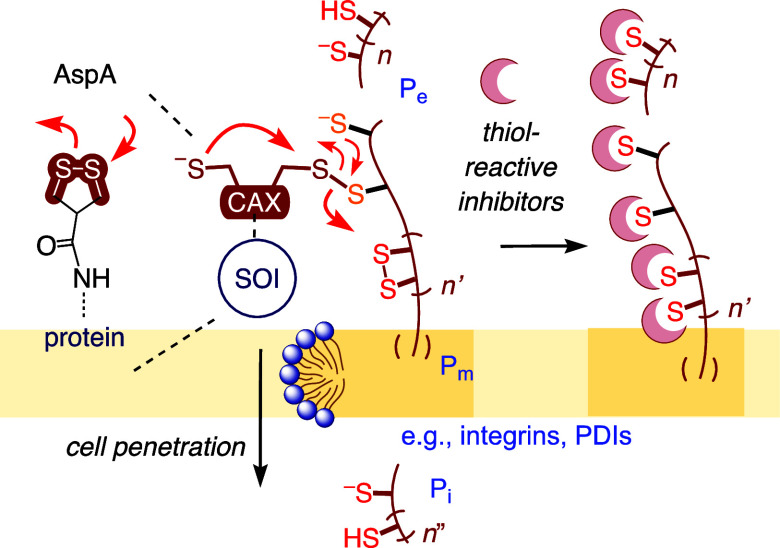
Thiol-mediated uptake (TMU) stands for the emergence of cell-penetrating activity in the presence of a cascade exchanger (CAX) attached to the substrate of interest (SOI, left), for example, a protein delivered with AspA, and the inhibition of this activity with thiol-reactive agents (right). The power of CAXs implies that TMU operates with exchange cascades including extracellular (P_e_), intracellular (P_i_), and membrane-related (P_m_) cellular partners.

Thiol-mediated uptake (TMU) refers to the cell-penetrating activity acquired by the attachment of thiol-reactive motifs to the substrates of interest (SOI),^1−8^ such as probes,^1,9^ drugs,^10^ proteins,^4,7,9,11−16^ oligonucleotides,^17−25^ liposomes,^24,26−28^ quantum dots,^9^ nanoparticles,^4,29,30^ and so on (Figure 1). Since this process appears to operate with dynamic covalent exchange reactions with cellular thiols and disulfides, e.g., cysteine and cystine residues, it can be inhibited by other thiol-reactive agents, which is the hallmark of TMU. So far, the best-performing thiol-reactive motifs are cascade exchangers (CAXs), such as cyclic disulfides, that are capable of multiple reversible exchanges with thiols and disulfides.^1,22,31^

**Figure 2:**
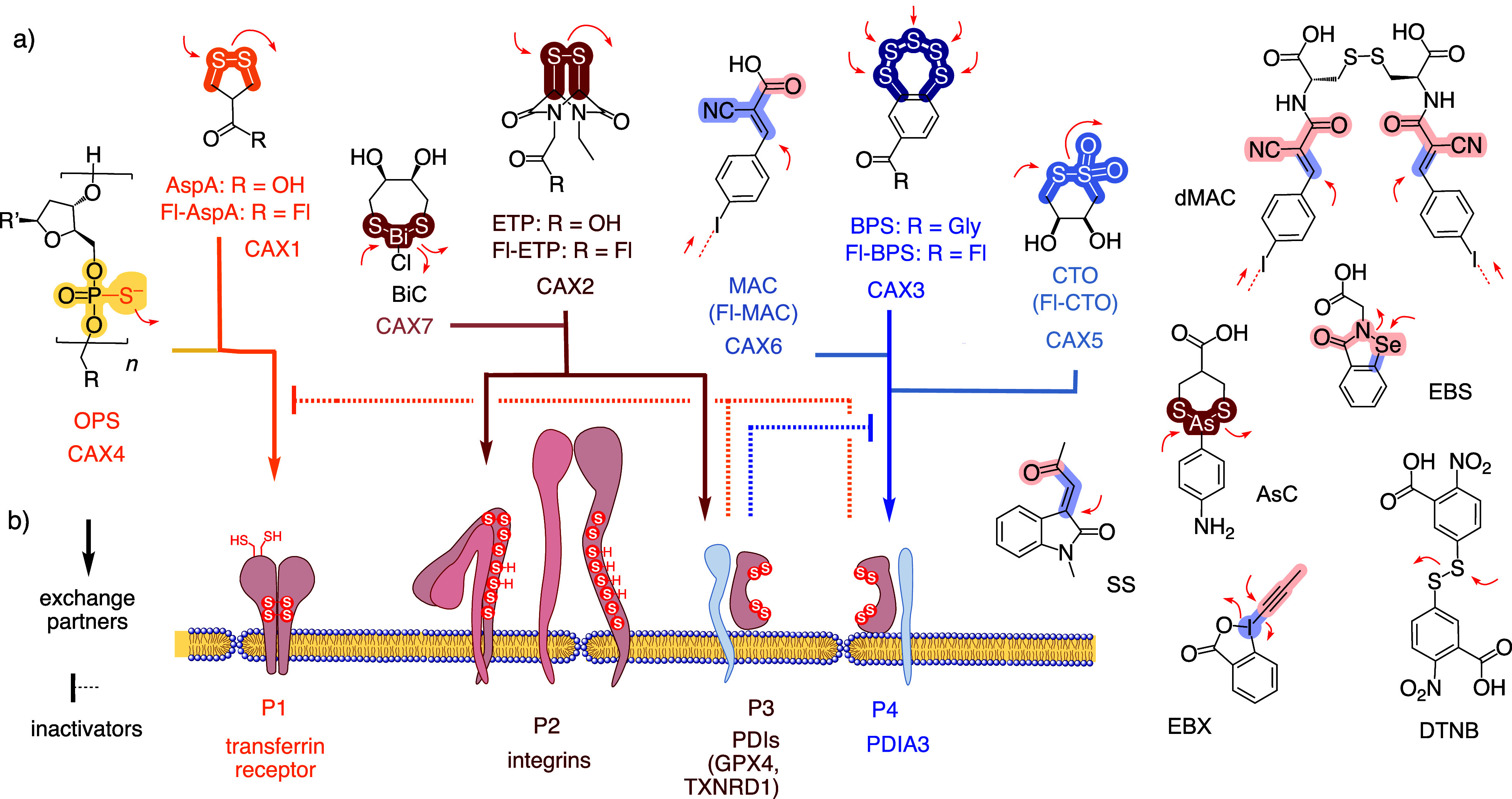
(a) CAX universe and (b) possible exchange partners (P) in thiol-mediated uptake, with new partners (P3 and P4) and three quasi-orthogonal networks assigned in this study to CAX1–7. Bold: Productive exchange partners, TMU inactivated by knockdown (KD), or alternative inhibitor (AI) of P; dashed: inactivators, KD or AI of P enhance TMU. OPS: R = Cy5, R′ = AGGTCCCCATACACCGAC. See Figure 3 for the structure of Fl in CAX1 and Figure S2 for complete structures of Fl-CAXs.

TMU has long been known from eclectic observations but without much follow-up.^1,32^ In recent years, however, a rapidly growing number of reports have demonstrated their power in advanced applications. Namely, TMU works also in vivo, i.e., animals (up to genome editing),^6,21,25,33,34^ plants,^17^ and bacteria,^10^ delivers well into deep tissue,^12,20,35^ and accounts for the cell-penetrating ability of antisense oligophosphorothioates (OPS, Figure 2b).^22−24^ Moreover, TMU inhibitors also attenuate the cellular entry of pathogens, including several viruses,^36,37^ and the cell motility.^38^ These activities suggest that TMU is relevant not only for drug delivery but also for drug discovery.

Despite this emerging importance, the molecular mechanism of TMU is essentially unknown.^1^ It is understood that the TMU can operate in combination with different uptake mechanisms. Examples exist for membrane fusion,^24^ including the first observation during viral entry,^36^ endocytosis,^12,13,15,18,20,22,35^ and mostly direct penetration through the plasma membrane.^1^ Accounting also for most endosomal escape, the problematic step during endocytosis, direct penetration, has attracted most attention for the cytosolic delivery of SOIs by TMU^1^ (the shift from endosomal capture to cytosolic delivery upon activation of TMU has been exemplified with OPS^22^). It has been recognized early on that size-independent movement across membranes is best conceivable through elastic toroidal membrane spots (also referred to as toroidal or micellar pores,^39^Figure 1).^1,40^ These toroidal elastics are believed to be central as for cell-penetrating peptides (CPPs)^15,16,40^ and do not deserve further attention in efforts to understand TMU. The key difference is that CPPs interact noncovalently with anionic membranes,^40,41^ while TMU operates with proteins by dynamic covalent exchange with thiols and disulfides.^1,40^ This shift from noncovalent interactions with membranes to dynamic covalent reactions with proteins is attractive to minimize toxicity from prolonged disordering membrane contacts and access selectivity inherent to protein chemistry. To elaborate on this distinctive nature of TMU, the identification of the proteins that participate as exchange partners in the dynamic covalent exchange cascades has thus emerged as the key question. The high activity of CAXs directly implied that TMU requires more than one exchange, that is, dynamic covalent exchange cascades (analogous to the noncovalent counterion exchange cascades at work with CPPs).^1,40^ These dynamic covalent exchange cascades with cellular thiols and disulfides are likely to include extracellular (P_e_),^15^ intracellular (P_i_), and membrane-associated proteins (P_m_, Figure 1), together with small molecules such as glutathione. For the most important membrane proteins, this could be limited to a single catch and release step to enable the passage through a toroidal elastic (*n*′ = 0) or involve longer cascades with one or more exchanges along the same (*n*′ > 1) or different proteins (Figure 1), with or without massive conformational changes during local temporary misfolding.

To answer these questions, a rich collection of chalcogen-,^42^ pnictogen-,^37^ and tetrel-centered CAXs^31,43^ has been developed (Figure 2a). The identification of their cellular exchange partners is expected to decode the TMU networks (Figure 2b). The objective of this study was to develop general methods to identify and assign these partners. The resulting pattern generation protocol identifies different protein disulfide isomerases (PDIs),^44−47^ particularly the multifunctional PDIA3,^48,49^ as TMU exchange partners besides integrins^38^ and the transferrin receptor,^50^ and reveals that the central CAXs known today enter cells along these three almost orthogonal cascade exchange networks.

## Results

### CAXs as Transporters

**Figure 3:**
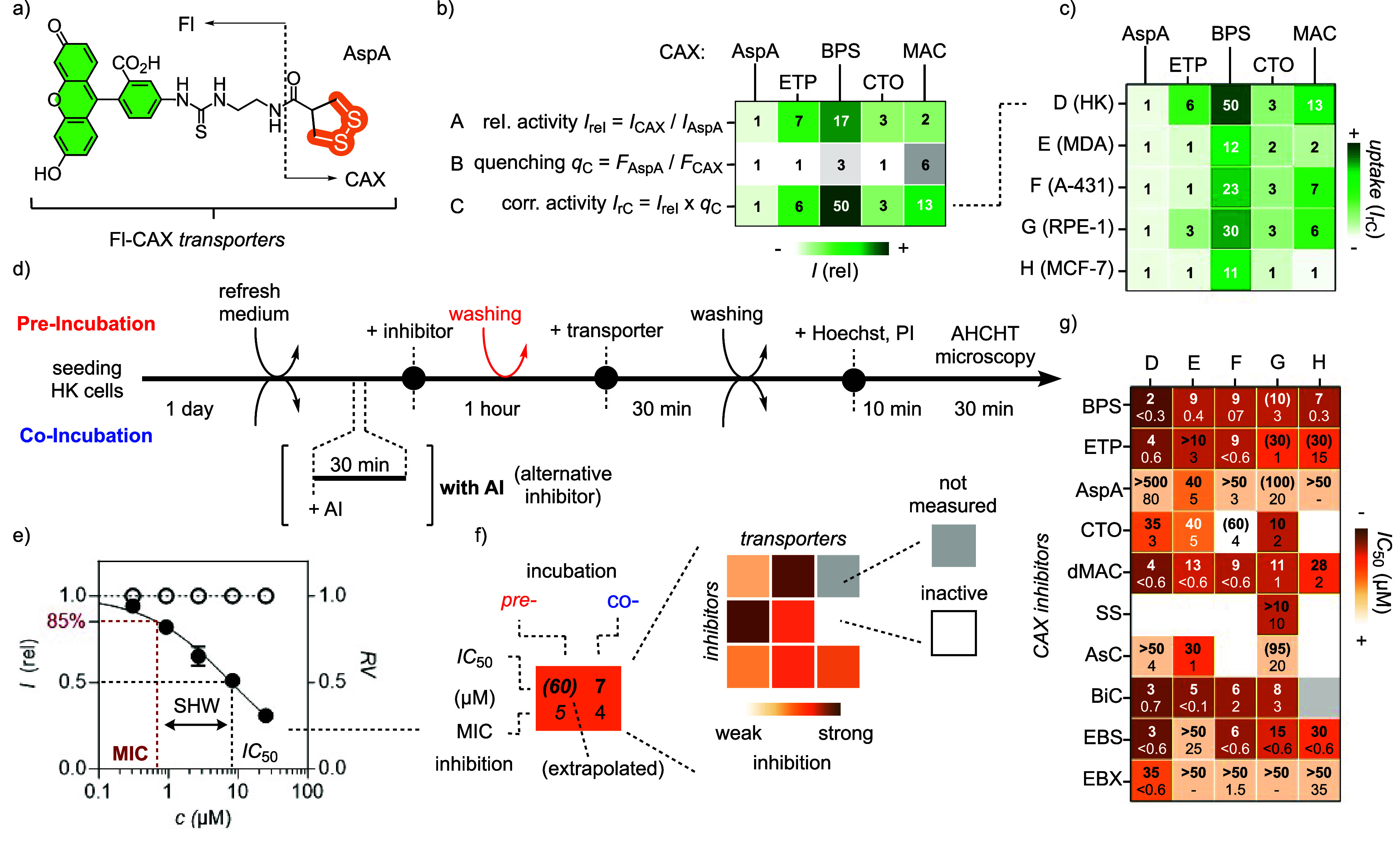
Methods development to report (a–c) the uptake activity and (d–h) the inhibition of CAX transporters. (a) Structure of Fl-CAXs as formal transporters of a fluorescent probe. See Figure S2 for complete structures. (b) Fluorescence intensity of Fl-CAX in HK cells compared to Fl-AspA (A, *I*_rel_), corrected for quenching (B, *q*_C_) due to the CAXs in the presence of DTT to give uptake activity (C, *I*_rC_). (c) Uptake of Fl-CAXs into different cell lines. (d) Workflow for inhibitor screening (PI, propidium iodide, added to label dead cells; Hoechst 33342 to label nuclei). (e) Representative AHCHT analysis of Fl-ETP uptake (constant concentration, filled circles) and relative cell viability (empty circles) as a function of the ETP concentration. SHW: Switching-half-window. (f) Transcription rules for pattern generation. (g) Dependence of Fl-ETP uptake inhibition by CAX on the nature of cells.

To characterize the TMU of different CAXs, they were labeled with a fluorescent probe, fluorescein, because its anionic nature minimizes passive diffusion into cells (Figure 3a). All Fl-CAXs were synthesized following previously reported procedures (Figures S1, S2).^31,42,51,52^ Uptake into HeLa Kyoto (HK) cells was evaluated under identical conditions by automated high-content high-throughput (AHCHT) microscopy.^43,53^ In this imaging-based technique, fluorescence images of thousands of cells are acquired and analyzed in an unbiased manner to give reliable information about the relative quantity and location of the probe in a very short time. Masks are automatically generated to distinguish between live and dead cells, allowing the measurement of fluorescence intensity only within live cells and reporting cell viability simultaneously. Within cells, different Fl-CAXs labeled different sites, suggesting that their mostly unknown intracellular targets are different.^51,52^ Since intracellular targeting is easily adjustable, for example with HaloTags,^35,54^ it is irrelevant in the context of this study.

The relative TMU activity of individual Fl-CAXs was reported as *I*_rel_, that is, the average fluorescence intensity *I*_CAX_ in cells after incubation with Fl-CAXs divided by *I*_AspA_ of Fl-AspA (Figure 3bA). For a more realistic assessment of TMU, the *I*_rel_ values were corrected with *q*_C_, quantifying the fluorescence quenching by the nearby CAX under cytosol mimetic reducing conditions (Figures 3bB, S3, S4, Table S1).^52^ The general trend of the resulting *I*_rC_ was comparable to that of uncorrected *I*_rel_ except for Fl-MAC, which would be underestimated with *I*_rel_ due to a heavy iodo quencher (Figures 3b, S6, S7).

The trends found in HK cells were reasonably well preserved in different cell lines, that is, MDA-MB-123 (Figure 3cE), an aggressive, invasive breast cancer cell line, A-431, a cancerous cell line derived from an epidermoid carcinoma tumor (F), RPE-1, a noncancerous retinal pigment epithelial cell line (G), and finally MCF-7, another breast cancer cell line (H). Global activities compared to HK cells were equal to or up to six times lower, except for Fl-MAC, which was 2–13 times less active (Figures 3c, S8–S13).

The re-evaluation under identical conditions confirmed the previous results and ranked the benzopolysulfanes (BPS) as the most powerful CAX of the series (Figure 2b, 3a–c).^52^ BPS occur in marine natural products with various biological activities. They cling to thiol/ate affinity columns and evolve into adaptive networks consisting of linear and cyclic oligomers through ring expansion, contraction, and opening upon exposure to thiol/ates.^52^ Epidithiodiketopiperazines (ETPs) are the second most active CAX without correction (Figure 3bA), in part due to their minimal fluorescence quenching. The bioinspired ETPs exchange ultrafast because ring tension is maximal, but are poorly retained on thiol/ate affinity columns.^51^ In CAXs derived from asparagusic acid (AspA),^55^ reduced ring tension compared to ETP coincides with reduced cell penetration, while less strained disulfides show even weaker activity.^51,52^

Cyclic thiosulfonates (CTOs) operate on a higher oxidation level, which accelerates the first and adds selectivity to the second step of cascade exchange, promising also against the entry of SARS-CoV-2 lentivectors.^42,56^ Reversible Michael acceptors (MACs)^57,58^ show good uptake, but suffer from the highest quenching due to the iodine, which is however essential for activity because it contributes a halogen-bonding^59,60^ switch for pseudocascade exchange.^43^ The cell-penetrating activity of OPS^22−24,61^ was not directly comparable because OPS are oligomers and labeled with a different fluorophore, i.e., Cy5 (Figure 2).^22^

### CAXs as Inhibitors

For the AHCHT screening of TMU inhibitors, the currently used standard protocol has matured over several years (Figure 3d). From AHCHT images, the TMU of Fl-CAX transporters in the presence of the inhibitor at various concentrations is analyzed and reported as IC_50_ and MIC, the minimal inhibitory concentration equal to IC_15_ (Figure 3f). Both values were valuable because the IC_50_ cannot always be reached and the difference between the two provided the switching-half-window (SHW), informing on cooperativity.^62,63^

In the preincubation protocol, incubation of cells with the inhibitors was followed by a washing step to remove the unbound inhibitors before the addition of transporters, while in “co-incubation” the washing was not performed (Figure 3d). Neither procedure is perfect. Preincubation generally gives lower inhibitory activities because even covalently bound inhibitors can be washed away depending on the reversibility of bonds. Higher activities found with co-incubation, on the other hand, could include misleading contributions from the reaction between transporter and inhibitor. For the construction of heatmaps, IC_50_ (bold) and MIC for pre- (italics) and co-incubation were introduced as a complete and consistent format for reporting (Figure 3f).

From the massive inhibitor screens realized in the past,^1,43,53^ only privileged motifs with distinct exchange characteristics were preserved (Figure 2). This included all CAXs used in transporters (Figure 3), with a negative charge in place of the fluorescent moiety. The tetrel-centered MAC exchanger was complemented by a dMAC dimer with much higher activity^31^ and “superspice” SS, a cinnamaldehyde analogue known to activate the pain receptor TRPA1 by conjugate addition of a cysteine residue on the cytosolic side.^1,64^ The hypervalent ethynyl benziodoxolone (EBX) is an example of an irreversible tetrel-centered thiol-reactive agent operating on hypervalent iodine chemistry.^65,66^ The pnictogen-centered CAXs AsC and BiC did not only excel as inhibitors of TMU but were promising also as entry inhibitors of SARS-CoV-2 lentivectors^37^ and of cell motility,^38^ and relate to diverse pertinent topics, from molecular walkers^67^ to Ehrlichs magic bullet.^68^ Similarly high activities supported the inclusion of the widely used dynamic covalent selenium-centered ebselen analogue (EBS).^37^ Ellman’s reagent DTNB (5,5′-dithio-bis(2-nitrobenzoic acid)) is the established, yet poorly performing standard thiol-reactive inhibitor in biology,^4,36,53,69,70^ proposed long ago to inhibit cellular entry of HIV by inhibiting PDIs on the cell surface.^1,36^ To probe for cytosolic delivery by TMU early on, millimolar DTNB concentrations were needed,^71^ that is, 3 orders of magnitude above the best inhibitors in the CAX collection. Not yet ready for consideration in this study were other privileged or emerging CAXs like tetrel-centered thiolactones and thioesters,^31^ higher phosphorothioates with exchange cascades centered on dynamic covalent phosphorus,^72^ cyclic diselenides,^73,74^ and the popular cell-penetrating poly(disulfide)s,^1,4^ mentioned here only to highlight the dimensions of the CAX universe waiting to be explored.

If not commercially available, TMU inhibitors were prepared as previously reported (Figure 2).^31,37,42,43,51,52,65^ Before initiating AHCHT screens, the dependence of inhibition on the nature of the cells was determined (Figures 3g, S22, Table S10). Even more pronounced than for transporters, CAX inhibitors were nearly independent of the nature of cells. Also as for CAX transporters,^51^ CAX inhibitors were hardly affected by the presence of serum, while irreversible covalent inhibitors were inactivated (not shown).

### The Central Heatmap

**Figure 4:**
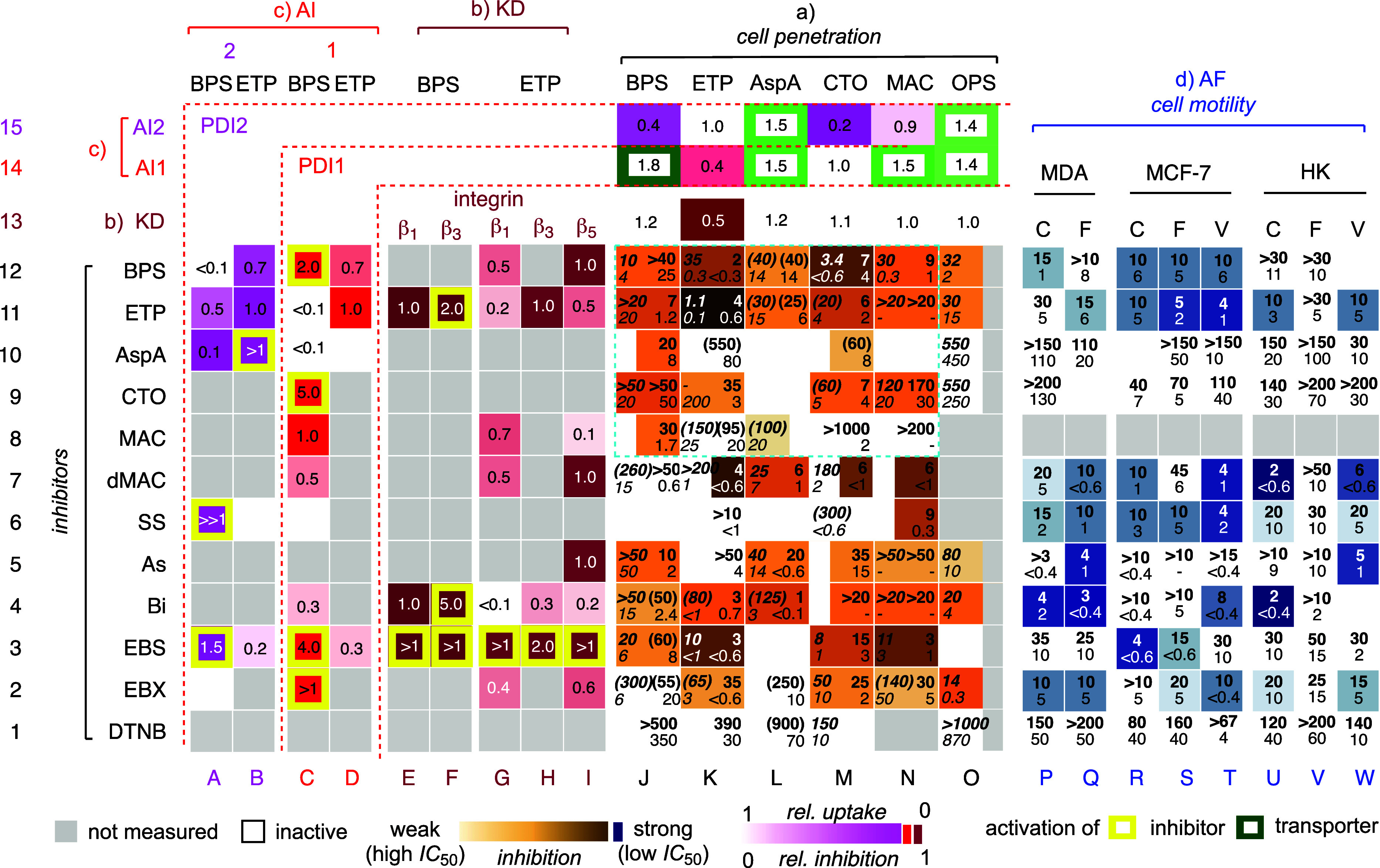
Inclusive heatmap to decode TMU networks, composed of (a) the central heatmap for the inhibition of Fl-CAX uptake surrounded by the impact of (b) knockdown (KD) and (c) inactivation by alternative inhibitors (AI) of potential TMU exchange partners on Fl-CAX uptake (horizonal) and their inhibition by various CAXs (vertical) and (d) the comparison with the inhibition of alternative function (AF). (a) Inhibition of Fl-CAX (J–O) uptake by inhibitors 1–12 into HK cells, reported as IC_50_ (top) and MIC (bottom, in μM) for pre- (left, italics) and co-incubation (right) of inhibitors (compare Figure 3). (b, c) Relative changes compared to WT upon (b) KD of integrins β_1_, β_3_, or β_5_ in uptake (J-O13 for β_1_, 1: no change (white), <1: less uptake (red)) and uptake inhibition IC_50_ (E1-I12, 1: no change (red), <1: weaker (toward white), >1: stronger inhibition (yellow frame)), and (c) addition of AI1 (16F16, 50 μM) for PDI P3 and AI2 (LOC14, 25 μM) for PDI P4 in uptake (J14-O15, 1: no change (white), <1: less uptake (red), >1: more uptake (green frame)) and Fl-ETP/BPS uptake inhibition (A1-D12, 1: no change (red), <1: less uptake (toward white), >1: more inhibition (yellow frame)). (d) MIC (top) and IC_50_ (bottom, in μM) for the inhibition of the motility of MDA-MB-231, MCF-7, and HK cells on collagen I (C), fibronectin (F), and vitronectin(V). All data in columns N,^43^ O,^22^ and P–W^38^ and parts of J, K,^37,53^ and M^42^ are from the literature.

In the central heatmap, the selected CAXs are compared as transporters and inhibitors of TMU, without further modifications applied to the system (Figure 4a, gold, J1-O12, Figures S14–S21, Tables S2–S9). Toward inclusive heatmap expansion, we adapted a chess players’ notation, labeling rows 1–15 and columns A–W. This notation allows one to pinpoint every entry precisely. Entry K11, for instance, describes the “self-inhibition” of a fluorescent ETP transporter by an invisible ETP inhibitor.

Most previously reported inhibition data were remeasured under uniform conditions. Inhibitor screening for one of the most popular transporters, Fl-AspA, was performed for the first time, while the same for Fl-BPS was also largely new (Figure 4a, L, and J). The inhibition of a single CAX transporter by different CAX inhibitors was listed in columns J–O. The ability of a single CAX inhibitor to inhibit different CAX transporters was listed in rows 1–12. The distinct pattern visible in the central heatmap showed that the inhibition activities do not simply depend on the reactivity, supporting the notion that more complex cascade exchange networks are underlying TMU.

### Effects of Protein Knockdown on TMU

The first addition to the central heatmap focused on the knockdown of possible cellular exchange partners in the TMU (Figure 4b). Previous knockdown studies have identified the transferrin receptor as an exchange partner P1 in TMU of Fl-AspA^50^ but not Fl-ETP (Figure 2b).^51^ The essential cysteines to initiate exchange cascades are far from the transferrin binding site. This finding was intriguing because the transferrin receptor has also been implicated in viral entry.^1^ Efficient transcytosis, needed to deliver iron to the brain, could explain why TMU of AspA conjugates excels at penetrating deep tissue.^12,20,35^

In this study, the knockdown of integrins was selected to uncover their role in TMU, as implied by our recent studies on cell motility (P2, Figure 2b).^38^ This was interesting because integrins are not only responsible for cell adhesion and motility but also involved in signal transduction, viral entry, thrombosis, and tumor progression. In addition, the β subunit of the integrin dimer contains one of the most spectacular linear disulfide arrays in nature (Figure 2b). The integrin family consists of 24 heterodimers. Their activation from inactive, bent conformers to active, linearized conformers can depend on glutathione and PDIs.^38^

**Figure 5:**
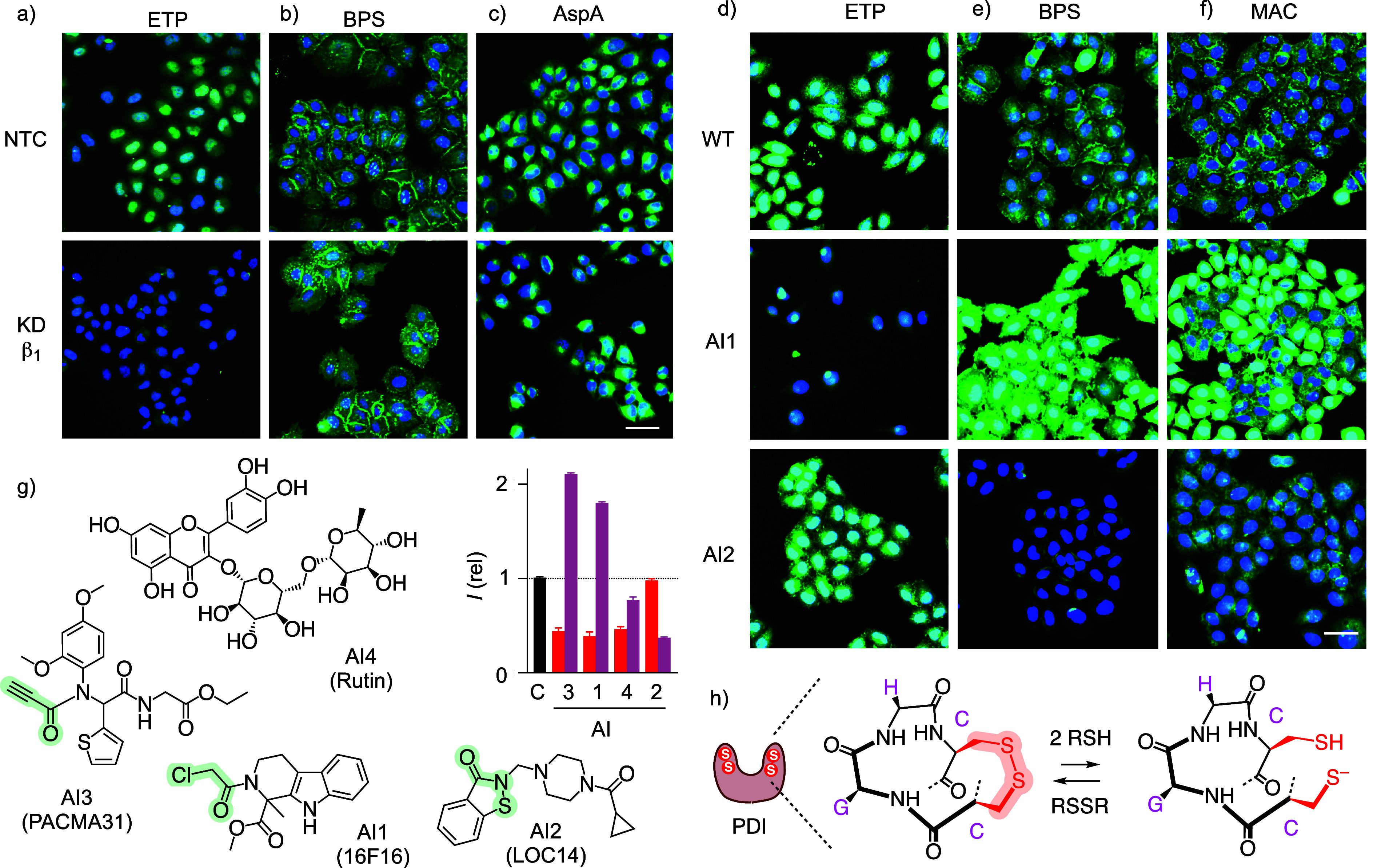
(a–c) Spinning disk confocal microscopy (SDCM) images of nontarget control (NTC, top) and INT β_1_ siRNA knockdown HK cells (bottom) incubated with Fl-ETP (a), Fl-BPS (b), and Fl-AspA (c, green; blue: Hoechst 33342, nuclei; scale bars, 30 μm). (d–f) Fluorescence images of wild-type HK cells incubated with Fl-ETP (d), BPS (e), and MAC (f, green) without (top) and with 16F16 (middle, AI1) or LOC14 (bottom, AI2; blue: Hoechst 33342, nuclei; scale bars: 50 μm). (g) Normalized fluorescence intensity of HK cells incubated with AI1–4 followed by Fl-ETP (red) or Fl-BPS (pink). (i) Schematic structure and function of the PDIs.

Using siRNA technology, we knocked down β_1_, β_3_, or β_5_ integrin subunits in HK cells (see Figure S24). Changes in the activity of CAX transporters in response to integrin knockdown were recorded by AHCHT imaging (Figure 5a–c). Strongly reduced TMU of Fl-ETP was clearly visible in confocal images (Figures 5a), while BPS, AspA, and other transporters were not affected much (Figures 5b,c, S25–S30). In the expanded heatmap, the representative results obtained with integrin β_1_-depleted cells were reported in row 13 as the relative fluorescence intensity compared to the wild-type cells (WT, Figure 4b). Among the colorless squares, red K13 stands out, revealing the selective deterioration of Fl-ETP uptake by integrin knockdown.

Changes in the ability of CAXs to inhibit TMU of ETP and BPS transporters in integrin-deficient cells were recorded under the co-incubation conditions (Figures S31–S36, Tables S11–S13) and reported in columns E–I as relative MIC values in WT/KD cells. The resulting heatmap consistently reflected the opposite responsiveness of Fl-ETP and Fl-BPS to integrin knockdown with weakened (G–I, lighter red) and enhanced inhibition (E, F, yellow frames), respectively.

### Effects of Alternative Inhibitors on TMU

To further expand the central TMU heatmap, alternative inhibitors (AIs) of possible protein partners were considered (Figure 4c). PDIs were selected to elaborate on this approach to inclusive pattern generation.^44,45^ Although the main role of the 21 different PDIs^44,49^ is to control protein folding in the ER, they have been observed throughout cells and on cell surfaces, involved in viral entry, signal transduction, integrin activation, and much more.^49,75,76^ PDIs are not membrane proteins and can be considered like giant cyclic disulfides analogous to AspA or ETP (Figure 2), the protein versions of CAXs (Figure 1), with macrocyclic disulfide ring tension and thiol acidity controlled by the α helix N-capping^77,78^ position of the CXXC motif^46,79,80^ and by neighboring ionic residues (Figure 4h).^47^

Four commercial PDI inhibitors were chosen as AI1 to AI4 (Figure 5d–g).^45^ AI1, known as 16F16, a thiol/ate reactive chloroacetamide, is a high-affinity, irreversible PDI inhibitor with rather poor selectivity.^45,81−84^ Similar to AI1 is AI3, the popular PACMA31, an irreversible Michael acceptor that reacts with a large group of PDIs.^85−87^ AI2, referred to as LOC14, is an allosteric, dynamic covalent inhibitor.^84,88^ Contrary to 16F16 and PACMA31, LOC14 also inhibits PDIA3 with an IC_50_ = 5 μM, by binding next to the active site, thereby locking the U-shaped PDI in the disulfide state (Figure 5g,h).^83^ This PDIA3 selectivity of AI2 contrasts with PDIA1, for instance, which is inhibited by 16F16 and LOC14 with equal efficiency.^84^ AI4 is rutin, one of several natural product antioxidants that allosterically inhibit PDIs with lower selectivity and efficiency.^88,89^

These alternative inhibitors were used at comparably high concentrations to ensure the best possible deactivation and, thus, removal of their target(s) from TMU networks. In the presence of inhibitor AI1, TMU of Fl-ETP was strongly reduced (Figure 5d). In contrast, the same inhibitor AI1 increased the TMU of Fl-BPS (Figure 5e) as well as most other Fl-CAXs (Figure 5f). Consistent with their similar modes of action, almost the same trend was observed with AI3 (Figure 5g). The noncovalent AI4 reduced TMU of Fl-ETP to a similar extent as AI1 and AI3, but did not increase the uptake of Fl-BPS. With nearly perfect complementarity, AI2 reduced the uptake of Fl-BPS but did not affect Fl-ETP (Figure 5d, e, g). These results implied that the target(s) of AI1 and AI3 are different from the target(s) of AI4 and orthogonal to the target(s) of AI2, a finding that might also be of interest for target identification in cellular redox homeostasis.^90,91^

In the inclusive heatmap, the impact of AI1 and AI2 on TMU of all measured CAX transporters was recorded in rows 14 and 15 (Figures 4c, S37–S53, Tables S14–S19). Weakened TMU, formally equivalent to uptake inhibition, was highlighted in red, implying that the respective AI target(s), referred to as PDI P3 and P4, might function as exchange partners in TMU of the respective CAXs. Increased TMU compared to the absence of AIs, highlighted with green frames, indicated that the respective AI targets PDI P3/4 inactivate TMU of the respective CAX.

Changes in the ability of CAXs to inhibit ETP and BPS transporters in the presence of AI1 and AI2 were recorded under modified co-incubation conditions (Figure 3d) and reported in columns A–D. Color codings were kept as for integrin knockdown (Figures 4c, A–D, S54–S59, Table S20–S22).

### Alternative Functions Affected by CAXs

To complete the expansion of the central TMU heatmap, alternative functions (AFs) of possible protein partners were compared (Figure 4d). The inhibition of cell motility by CAX inhibitors has been recently explored to elaborate on integrins as possible exchange partners in TMU (Figure 2b).^38^ An AHCHT procedure was developed to record heatmaps for three different types of cells moving on three different surfaces, that is, collagen 1 (C), fibronectin (F), and vitronectin (V). To expand pattern generation, this existing heatmap on AF was integrated into the inclusive TMU heatmap (Figure 4d, P1-W12). As for TMU (J1-O12), motility inhibition heatmaps generated a distinct pattern that went beyond simple reactivity. In principle, similarities between the patterns generated for TMU and motility should indicate TMU networks including integrins.

## Discussion

### The Inclusive Heatmap

The collection of the above data produced an inclusive TMU heatmap that compares CAX inhibitors (1–12) with CAX transporters (J–O), partner knockdown (E–I), alternative inhibitors (A–D), and alternative functions (P–W, Figure 4). In general, this inclusive pattern generation protocol was expected to reveal the dynamic covalent cascade exchange networks responsible for thiol-mediated uptake (Figure 2, and alternative functions such as cell motility or redox homeostasis). First to note, the consistently poor performance of Ellman’s reagent DTNB throughout the inclusive heatmap was not surprising^53^ but alarming because of its frequent use in biological studies^4,36,53,69,70^ (Figure 4, J-W1).

### Three Orthogonal Networks

Patterns generated by the inclusive heatmap (Figure 4) suggested that the four most popular CAXs operate along three almost orthogonal dynamic covalent cascade exchange networks to penetrate cells (Figure 2b). AspA as CAX1 with transferrin receptor as exchange partner P1 during TMU has been identified previously.^50^ Now, our results add that this *AspA network* is hindered by PDIs P3 and P4, and OPS as CAX4 uses the same cellular exchange partners to penetrate cells. The *ETP network* introduces integrins P2 and PDIs P3 as specific exchange partners in TMU, while the transferrin receptor^50^ and PDI P4 are not involved in the ETP pathway. The *BPS network*, finally, introduces PDIA3 as an essential exchange partner P4, while integrins do not participate, and other PDIs P3 inhibit TMU.

### The ETP Pathway

Already the pattern produced by CAX inhibitors in the central heatmap hinted toward a quasi-3D orthogonality of ETP (K1–12) compared to BPS (J1–12) and AspA transporters (L1–12). The ETP pathway emerged most visibly from 50% TMU suppression upon integrin knockdown, either of the β_1_, β_3_, or β_5_ subunit (K13, Figures 5a, S26). This dependence on integrin was unique; other transporters were almost integrin independent. Around 50% residual activity without one subunit was consistent with the participation of all β_1_, β_3_, and β_5_ integrin subunits and the existence of additional exchange partners in the ETP pathway.

In agreement with these interpretations, the powerful self-inhibition of ETP transporters with ETP inhibitors (K11) weakened without β_5_ (I11) and particularly β_1_ (G11). In contrast, ETP uptake in the absence of integrin β_1_ (G12) and particularly β_5_ (I12) could still be well inhibited by the less integrin-dependent BPS, hinting again at exchange partners other than integrins in the ETP pathway.

Inhibition of ETP uptake by alternative inhibitor 1 (AI1, 16F16) was similarly unique (K14). It was contrary to the increased TMU of BPS (J14) and AspA (L14). Controls excluded direct exchange of ETP and other CAXs with AI1 under uptake conditions (Figure S37). Therefore, a 60% decrease of uptake in entry K14 supported that at least one of the targets of AI1 operates as exchange partner P3 in the ETP pathway (Figure 2b). Since PDIs are known to be involved in the activation of integrins, the two exchange partners P2 and P3 in the ETP pathway might be coupled. However, since 16F16 is a poorly selective irreversible inhibitor and is also similar to inhibitors of other proteins, for example, RSL3, the identity of P3 could not be defined at this point and could include partners beyond PDIs, such as GPX4 (glutathione peroxidase 4) or TXNRD1 (thioredoxin reductase 1) involved in ferroptosis.^90,91^ Preliminary results indicated that TMU inhibition patterns of 16F16 and RSL3 are nearly the same, but RSL3 is more toxic (not shown).

Insensitivity of the ETP uptake to AI2 (LOC14) was similarly unique (K15). This suggested that specific targets of AI2, particularly PDIA3,^48,49^ do not participate in the ETP pathway (Figure 2b). Finally, the similarity in the inhibition patterns generated for ETP uptake (K1–12) and cell motility (Figure 4d) was consistent with integrins as exchange partners in the ETP pathway (Figure 4d). Overall superior cell motility inhibition by ETP compared to BPS and AspA (P-W 11 vs 10 and 12) was further in support of this hypothesis.

### The BPS Pathway

The most distinct feature for the BPS pathway was around 60% TMU attenuation upon deactivation of PDI P4 with AI2 (J15), while ETP was insensitive (K15) and AspA was enhanced (L15). Controls confirmed that BPS and other CAX do not exchange directly with AI2 under uptake conditions (Figure S38). Entry J15 thus supported that this PDI operates as exchange partner P4 in the network, accounting for TMU of BPS (Figure 2b). In contrast, the presence of inhibitor AI1 increased TMU of BPS (J14). Entry J14 thus implied that the PDIs P3, which serve as exchange partners of ETP (K14), hinder TMU of BPS. Contrary to ETP (K13), insensitivity to integrin β_1_, β_3_, and β_5_ knockdown suggested that they are not involved as exchange partners in the BPS pathway (J13).

Like the poor self-inhibition of ETP transporters without most integrins (G11, I11), the self-inhibition of BPS transporters was completely abolished upon inactivation of its main exchange partner, P4 (A12). Like the integrin-insensitive BPS continuing to inhibit ETP transporters without integrins (I12, partially G12), the P4-insensitive ETP continued to partially inhibit BPS transporters without active PDI P4 (A11). With less binding to integrins, the ability of ETP to inhibit BPS transporters remained (E11) or even increased (F11). With less binding to P3, the ability of ETP to inhibit BPS transporters, however, vanished (C11). This contrast could be understood by the increased activity of BPS transporters without P3 (J14) but much less so without integrins (J13). Without unproductive binding to P3, the effective concentration of BPS transporters should thus increase and outcompete ETP inhibitors to afford C11. These patterns were, overall, remarkably consistent.

Based on these insights, we assigned the exchange partner P4 in the BPS pathway to PDIA3, which is a target of LOC14 but not of 16F16.^83^ This PDIA3, also known as ERp57, P58, ER60, ERp60, ERp61, GRP57, GRP58, PI-PLC, HsT17083, HEL-S-269, HEL-S-93n, and 1,25D3-MARRS, has been observed at the cell surface, involved in many processes, such as signal transduction, translocation, viral entry, and redox homeostasis.^48,49,75,83,84^ The many PDIA3 partners known in the literature include caveolins,^76^ integrins, EGFR, vitamin D,^76,92^ MHC I, angiotensin II, vasopressin, and calreticulin.^48^ However, the assignment of PDIA3 as an exchange partner in the BPS network is made with less confidence than of integrins to ETP and transferrin receptor to AspA, since it is based on inactivation by competitive inhibitors rather than on knockdown.

### The AspA Pathway

The AspA pathway has been suggested previously to operate with the transferrin receptor.^50^ To this, the inclusive heatmap added that the AspA pathway does not include integrins (L13) and is hindered by both PDI P3 (L14) and P4 (L15). Indeed, in contrast to ETP and BPS transporters, TMU of AspA was enhanced in each case when PDIs P3 and P4 were formally removed using AI (L14, 15).

### OPS Enter Cells along the AspA Pathway

Already the similarity of patterns generated in the central heatmap for OPS transporters (O1–12) and AspA transporters (L1–12) suggested that they might enter cells through a shared exchange network (Figure 2b). Like AspA, OPS transporters were insensitive to integrin knockdown (O13) and activated by removal of PDI P3 (O14) and P4 (O15). Additional co-localization experiments with fluorescently labeled transferrin conjugate and OPS resulted in more than 50% overlap (Figures S60–S65), supporting that transferrin receptors are the exchange partners of the OPS used in this study. However, pertinent literature suggested that this conclusion could be limited to the OPS used and might depend strongly on OPS sequence.^23,61^

### CTOs Follow the BPS Pathway

The almost complete loss of uptake activity in the presence of AI2 implied that CTO^42^ as CAX5 penetrates cells along the BPS pathway with PDIA3 as exchange partner P4 (M15, Figure 2b). This hypothesis was supported by similar patterns in the central heatmap (J, M1–12) and poor performance of CTOs in inhibiting cell motility (P-W9).

To a lesser extent, MAC transporters followed the same trend indicative of the BPS pathway (N) and were tentatively labeled CAX6 (Figure 2).

### Pnictogen-Centered CAXs Follow the ETP Pathway

Among CAXs available as inhibitors but not as transporters, the patterns generated by pnictogen-centered BiC^37^ and AsC^37^ were indicative of the ETP pathway with integrins and PDIs P3 as exchange partners (Figure 2). Upon integrin knockdown, BiC failed to inhibit ETP transporters (G-I4) and gained activity in inhibiting BPS transporters (F4) to an extent that exceeded the sensitivity of ETP inhibitors (E-I11). BiC also reproduced the peculiar failure of ETP to inhibit BPS transporters in the presence of AI1 (C4, C11). Thus, tentatively labeled as CAX7 (Figure 2), BiC also was an overall excellent inhibitor of cell motility (P-W4).

For other CAXs, patterns will need further development to draw conclusions with confidence. Occasional strong increases in inhibitory activity should not be overestimated when original inhibition is very weak. EBS showed patterns reminiscent of the ETP pathway (E-I3) but inhibited cell motility only weakly (P-W3). Interestingly, the tetrel-centered Michael acceptors SS and dMAC seemed to inhibit motility better than TMU (P6-W7). Tentatively, this observation might place SS and dMAC also close to integrins and the ETP pathway.

## Conclusion

Thiol-mediated uptake is currently emerging as a fascinating enigma in chemistry and biology. Increasingly appreciated in bringing matter into cells, the phenomenon is essentially not understood. Different lines of evidence suggest that TMU operates with cascade exchange networks that include diverse cellular exchange partners P which may differ from CAX to CAX. Before this study, all that was known was the transferrin receptor as P1, assigned as an exchange partner of AspA as CAX1, and integrins as partner P2 of not yet specified CAXs. With the pattern generation protocol developed in this study, four orthogonal exchange partners P1–P4 could be assigned to the four currently most popular CAXs, CAX1–CAX4 (Figure 2b). The *ETP pathway* operates with integrins and PDIs P3 as exchange partners, while the transferrin receptor and PDI P4 are not involved. The *BPS pathway* offers PDIA3 as an exchange partner P4, does not involve integrin P2, and is hindered by PDIs P3. The *AspA pathway* with the transferrin receptor as confirmed exchange partner P1 is insensitive to integrin P2 and hindered by PDIs P3 and P4. The selectivities differentiating these three quasi-orthogonal pathways are remarkably significant and consistent.

Among other CAXs covered, CTOs and in part also MACs follow the BPS pathway, while pnictogen-centered BiC and in part also AsC exchange along the ETP pathway. OPS follow the AspA pathway, suggesting that cell penetration of at least the here-used OPS occurs by TMU with transferrin receptor and can be enhanced by removing PDIs P3 and P4 with their respective inhibitors. Complementary to the previously reported activation with pseudo-disulfide-forming reagents like MMTS,^22^ this finding could be of interest to improve cell-penetrating antisense oligonucleotide phosphorothioates.^22−24,61^

In general, the activation of TMU by removal of nonproductive “anti-partners”, here PDIs, with the respective alternative inhibitors is a finding of immense practical importance. The use of alternative inhibitors is identified as a powerful complement to genetic knockdown for decoding TMU and identifying new exchange partners. The formal PDI inhibitors tested produced different patterns in the inclusive heatmap, suggesting that the developed protocol to crack TMU might also serve well to discriminate and assign targets in redox biology. The newly identified PDIA3^48,49^ as exchange partner in the BPS pathway adds a new facet to this intriguing multifunctional PDI and fascinating new directions for TMU to explore.

Analogous to the methods introduced in this study to decode dynamic covalent networks accounting for TMU, method development will be needed to elucidate their selectivity on the molecular level. The preference of CAXs for specific cellular exchange partners is not dictated by simple noncovalent host–guest chemistry with strongest ground-state stabilization being best. Goldilocks-type intermediate binding will be better, as in catalysis.^93^ Too strong binding would favor inhibition over penetration, resulting in prolonged plasma membrane disorganization, usually toxic, as exemplified recently with Fl-dMAC, naturally excluded as transporter in this study.^31^ In contrast, differences in reactivity, that is, complex coupled kinetics, will contribute, at best, to determine preferences in dynamic covalent cascade exchange chemistry.

The assignment of CAXs to cellular exchange partners realized in this study ignores the dependence on the substrate of interest, particularly substrate size. Recent results with cell-penetrating GFP-AspA conjugates were similar to the patterns generated with Fl-AspA (Figure 4a), with five out of seven matching inhibitors, including the top two.^12^ Nevertheless, existing reports on SOI dependence of the absolute activity of CAX suggest that pattern generation will also turn out to be SOI dependent to an extent that remains to be explored CAX by CAX.

The present study focuses on decoding exchange networks but does not explain how TMU really achieves penetration, the crossing of the plasma membrane. As stated in the Introduction, size-independent movement across membranes is best conceivable with toroidal elastics, which is similar to CPPs.^1,40^ Methods to confirm the existence and nature of toroidal elastics during TMU in living cells remain to be developed but are not essential for progress with TMU because its distinguishing characteristics originate from cascade exchange networks with cellular proteins.

With the expanding CAX universe, new knockdowns, AIs, and AFs will be realized to further enlarge inclusive heatmaps. The ultimate objective would be a complete set of CAXs assigned to their partners, decoded for reliable cellular uptake and inhibition of cellular entry, and ready to respond to any new challenge (Figure 2). The integrative protocol developed in this study provides the method needed to achieve this most demanding objective.

## Supporting Information Available

Materials and methods, synthesis of transporters (Fl-CAXs) and inhibitors (CAXs), cell culture, AHCHT imaging of cellular uptake, with general experimental and data analysis procedures, quenching factors for Fl-CAXs, AHCHT inhibitor screening, uptake and inhibition in integrin knockdown cells, uptake and inhibition in the presence of PDI inhibitors, co-localization of OPS-Cy5 and transferrin CF488A conjugate, and supporting references (PDF)

## References

[ref1] Laurent Q.; Martinent R.; Lim B.; Pham A.-T.; Kato T.; López-Andarias J.; Sakai N.; Matile S. Thiol-Mediated Uptake. JACS Au 2021, 1, 710–728. 10.1021/jacsau.1c00128. 34467328 PMC8395643

[ref2] Wan Y.; Wang W.; Lai Q.; Wu M.; Feng S. Advances in Cell-Penetrating Poly(disulfide)s for Intracellular Delivery of Therapeutics. Drug Discovery Today 2023, 28, 103668 10.1016/j.drudis.2023.103668. 37321318

[ref3] Ulrich S. Growing Prospects of Dynamic Covalent Chemistry in Delivery Applications. Acc. Chem. Res. 2019, 52, 510–519. 10.1021/acs.accounts.8b00591. 30676745

[ref4] Du S.; Liew S. S.; Li L.; Yao S. Q. Bypassing Endocytosis: Direct Cytosolic Delivery of Proteins. J. Am. Chem. Soc. 2018, 140, 15986–15996. 10.1021/jacs.8b06584. 30384589

[ref5] Zhou J.; Shao Z.; Liu J.; Duan Q.; Wang X.; Li J.; Yang H. From Endocytosis to Nonendocytosis: The Emerging Era of Gene Delivery. ACS Appl. Bio Mater. 2020, 3, 2686–2701. 10.1021/acsabm.9b01131. 35025403

[ref6] Chen Y.; Ping Y. Development of CRISPR/Cas Delivery Systems for In Vivo Precision Genome Editing. Acc. Chem. Res. 2023, 56, 2185–2196. 10.1021/acs.accounts.3c00279. 37525893

[ref7] Chen N.; He Y.; Zang M.; Zhang Y.; Lu H.; Zhao Q.; Wang S.; Gao Y. Approaches and Materials for Endocytosis-Independent Intracellular Delivery of Proteins. Biomaterials 2022, 286, 121567 10.1016/j.biomaterials.2022.121567. 35580476

[ref8] Lu F.; Zhang H.; Pan W.; Li N.; Tang B. Delivery Nanoplatforms Based on Dynamic Covalent Chemistry. Chem. Commun. 2021, 57, 7067–7082. 10.1039/D1CC02246F. 34195709

[ref9] Derivery E.; Bartolami E.; Matile S.; Gonzalez-Gaitan M. Efficient Delivery of Quantum Dots into the Cytosol of Cells Using Cell-Penetrating Poly(disulfide)s. J. Am. Chem. Soc. 2017, 139, 10172–10175. 10.1021/jacs.7b02952. 28741941 PMC5553715

[ref10] Shchelik I. S.; Gademann K. Synthesis and Antimicrobial Evaluation of New Cephalosporin Derivatives Containing Cyclic Disulfide Moieties. ACS Infect. Dis. 2022, 8, 2327–2338. 10.1021/acsinfecdis.2c00393. 36251034

[ref11] Lu J.; Wang H.; Tian Z.; Hou Y.; Lu H. Cryopolymerization of 1,2-Dithiolanes for the Facile and Reversible Grafting-from Synthesis of Protein-Polydisulfide Conjugates. J. Am. Chem. Soc. 2020, 142, 1217–1221. 10.1021/jacs.9b12937. 31927989

[ref12] Maynard J. R. J.; Saidjalolov S.; Velluz M.-C.; Vossio S.; Aumeier C.; Moreau D.; Sakai N.; Matile S. Toward a Traceless Tag for the Thiol-Mediated Uptake of Proteins. ChemistryEurope 2023, 1, e202300029 10.1002/ceur.202300029.

[ref13] Goerdeler F.; Reuber E. E.; Lühle J.; Leichnitz S.; Freitag A.; Nedielkov R.; Groza R.; Ewers H.; Möller H. M.; Seeberger P. H.; et al. Thiol-Mediated Uptake of a Cysteine-Containing Nanobody for Anticancer Drug Delivery. ACS Cent. Sci. 2023, 9, 1111–1118. 10.1021/acscentsci.3c00177. 37396861 PMC10311659

[ref14] Meng X.; Li T.; Zhao Y.; Wu C. CXC-Mediated Cellular Uptake of Miniproteins: Forsaking “Arginine Magic.. ACS Chem. Biol. 2018, 13, 3078–3086. 10.1021/acschembio.8b00564. 30272440

[ref15] Arafiles J. V. V.; Hirose H.; Hirai Y.; Kuriyama M.; Sakyiamah M. M.; Nomura W.; Sonomura K.; Imanishi M.; Otaka A.; Tamamura H.; et al. Discovery of a Macropinocytosis-Inducing Peptide Potentiated by Medium-Mediated Intramolecular Disulfide Formation. Angew. Chem., Int. Ed. 2021, 60, 11928–11936. 10.1002/anie.202016754. 33629482

[ref16] Arafiles J. V. V.; Franke J.; Franz L.; Gómez-González J.; Kemnitz-Hassanin K.; Hackenberger C. P. R. Cell-Surface-Retained Peptide Additives for the Cytosolic Delivery of Functional Proteins. J. Am. Chem. Soc. 2023, 145, 24535–24548. 10.1021/jacs.3c05365. 37906525 PMC10655119

[ref17] Mou Q.; Xue X.; Ma Y.; Banik M.; Garcia V.; Guo W.; Wang J.; Song T.; Chen L.-Q.; Lu Y. Efficient Delivery of a DNA Aptamer-Based Biosensor into Plant Cells for Glucose Sensing through Thiol-Mediated Uptake. Sci. Adv. 2022, 8, eabo0902 10.1126/sciadv.abo0902. 35767607 PMC9242441

[ref18] Shu Z.; Tanaka I.; Ota A.; Fushihara D.; Abe N.; Kawaguchi S.; Nakamoto K.; Tomoike F.; Tada S.; Ito Y.; et al. Disulfide-Unit Conjugation Enables Ultrafast Cytosolic Internalization of Antisense DNA and siRNA. Angew. Chem., Int. Ed. 2019, 58, 6611–6615. 10.1002/anie.201900993. 30884043

[ref19] Zhou J.; Sun L.; Wang L.; Liu Y.; Li J.; Li J.; Li J.; Yang H. Self-Assembled and Size-Controllable Oligonucleotide Nanospheres for Effective Antisense Gene Delivery through an Endocytosis-Independent Pathway. Angew. Chem., Int. Ed. 2019, 58, 5236–5240. 10.1002/anie.201813665. 30809927

[ref20] Kohata A.; Hashim P. K.; Okuro K.; Aida T. Transferrin-Appended Nanocaplet for Transcellular siRNA Delivery into Deep Tissues. J. Am. Chem. Soc. 2019, 141, 2862–2866. 10.1021/jacs.8b12501. 30724083

[ref21] Guo J.; Wan T.; Li B.; Pan Q.; Xin H.; Qiu Y.; Ping Y. Rational Design of Poly(disulfide)s as a Universal Platform for Delivery of CRISPR-Cas9Machineries toward Therapeutic Genome Editing. ACS Cent. Sci. 2021, 7, 990–1000. 10.1021/acscentsci.0c01648. 34235260 PMC8227594

[ref22] Laurent Q.; Martinent R.; Moreau D.; Winssinger N.; Sakai N.; Matile S. Oligonucleotide Phosphorothioates Enter Cells by Thiol-Mediated Uptake. Angew. Chem., Int. Ed. 2021, 60, 19102–19106. 10.1002/anie.202107327. PMC8456962 34173696

[ref23] Batistatou N.; Kritzer J. A. Investigation of Sequence-Penetration Relationships of Antisense Oligonucleotides. ChemBioChem. 2023, 24, e202300009 10.1002/cbic.202300009. 36791388 PMC10305730

[ref24] Yan A.; Chen X.; He J.; Ge Y.; Liu Q.; Men D.; Xu K.; Li D. Phosphorothioated DNA Engineered Liposomes as a General Platform for Stimuli-Responsive Cell-Specific Intracellular Delivery and Genome-Editing. Angew. Chem., Int. Ed. 2023, 62, e202303973 10.1002/anie.202303973. 37100742

[ref25] Zhou J.; Zhang J.; Chen S.; Lin Q.; Zhu R.; Wang L.; Chen X.; Li J.; Yang H. Direct Cytoplasmic Delivery of RNAi Therapeutics through a Non-Lysosomal Pathway for Enhanced Gene Therapy. Acta Biomater. 2023, 170, 401–414. 10.1016/j.actbio.2023.08.039. 37625679

[ref26] Qualls M. L.; Lou J.; McBee D. P.; Baccile J. A.; Best M. D. Cyclic Disulfide Liposomes for Membrane Functionalization and Cellular Delivery. Chem.—Eur. J. 2022, 28, e202201164 10.1002/chem.202201164. 35699671

[ref27] Li T.; Takeoka S. Enhanced Cellular Uptake of Maleimide-Modified Liposomes via Thiol-Mediated Transport. Int. J. Nanomedicine 2014, 9, 2849–2861. 10.2147/IJN.S58540. 24940060 PMC4051732

[ref28] Chuard N.; Gasparini G.; Moreau D.; Lörcher S.; Palivan C.; Meier W.; Sakai N.; Matile S. Strain-Promoted Thiol-Mediated Cellular Uptake of Giant Substrates: Liposomes and Polymersomes. Angew. Chem., Int. Ed. 2017, 56, 2947–2950. 10.1002/anie.201611772. 28261969

[ref29] Knoll P.; Francesco Racaniello G.; Laquintana V.; Veider F.; Saleh A.; Seybold A.; Denora N.; Bernkop-Schnürch A. Lipid-Based Nanoparticles: Enhanced Cellular Uptake via Surface Thiolation. Int. J. Pharm. 2023, 635, 122753 10.1016/j.ijpharm.2023.122753. 36863545

[ref30] Kanjilal P.; Dutta K.; Thayumanavan S. Thiol-Disulfide Exchange as a Route for Endosomal Escape of Polymeric Nanoparticles. Angew. Chem., Int. Ed. 2022, 61, e202209227 10.1002/anie.202209227. PMC9452476 35866880

[ref31] Lim B.; Sakai N.; Matile S. Tetrel-Centered Exchange Cascades to Decouple Inhibition and Induction of Thiol-Mediated Uptake: Introducing Cell-Penetrating Thiolactones, Focus on Reversible Michael Acceptor Dimers. Helv. Chim. Acta 2023, 106, e202300020 10.1002/hlca.202300020.

[ref32] Torres A. G.; Gait M. J. Exploiting Cell Surface Thiols to Enhance Cellular Uptake. Trends Biotechnol. 2012, 30, 185–190. 10.1016/j.tibtech.2011.12.002. 22260747

[ref33] Zhu Y.; Lin M.; Hu W.; Wang J.; Zhang Z.-G.; Zhang K.; Yu B.; Xu F.-J. Controllable Disulfide Exchange Polymerization of Polyguanidine for Effective Biomedical Applications by Thiol-Mediated Uptake. Angew. Chem., Int. Ed. 2022, 61, e202200535 10.1002/anie.202200535. 35304808

[ref34] Hei M.-W.; Zhan Y.-R.; Chen P.; Zhao R.-M.; Tian X.-L.; Yu X.-Q.; Zhang J. Lipoic Acid-Based Poly(disulfide)s as Versatile Biomolecule Delivery Vectors and the Application in Tumor Immunotherapy. Mol. Pharmaceutics 2023, 20, 3210–3222. 10.1021/acs.molpharmaceut.3c00231. 37150945

[ref35] Martinent R.; Tawffik S.; López-Andarias J.; Moreau D.; Laurent Q.; Matile S. Dithiolane Quartets: Thiol-Mediated Uptake Enables Cytosolic Delivery in Deep Tissue. Chem. Sci. 2021, 12, 13922–13929. 10.1039/D1SC04828G. 34760179 PMC8549803

[ref36] Ryser H. J.-P.; Flückiger R. Keynote Review: Progress in Targeting HIV-1 Entry. Drug Discovery Today 2005, 10, 1085–1094. 10.1016/S1359-6446(05)03550-6. 16182193

[ref37] Lim B.; Kato T.; Besnard C.; Poblador Bahamonde A. I.; Sakai N.; Matile S. Pnictogen-Centered Cascade Exchangers for Thiol-Mediated Uptake: As(III)-, Sb(III)-, and Bi(III)-Expanded Cyclic Disulfides as Inhibitors of Cytosolic Delivery and Viral Entry. JACS Au 2022, 2, 1105–1114. 10.1021/jacsau.2c00017. 35615714 PMC9063988

[ref38] Coelho F.; Saidjalolov S.; Moreau D.; Thorn-Seshold O.; Matile S. Inhibition of Cell Motility by Cell-Penetrating Dynamic Covalent Cascade Exchangers: Integrins Participate in Thiol-Mediated Uptake. JACS Au 2023, 3, 1010–1016. 10.1021/jacsau.3c00113. 37124287 PMC10131202

[ref39] Matsuzaki K.; Murase O.; Fujii N.; Miyajima K. An Antimicrobial Peptide, Magainin 2, Induced Rapid Flip-Flop of Phospholipids Coupled with Pore Formation and Peptide Translocation. Biochemistry 1996, 35, 11361–11368. 10.1021/bi960016v. 8784191

[ref40] Gasparini G.; Bang E.-K.; Montenegro J.; Matile S. Cellular Uptake: Lessons from Supramolecular Organic Chemistry. Chem. Commun. 2015, 51, 10389–10402. 10.1039/C5CC03472H. 26030211

[ref41] Takeuchi T.; Kosuge M.; Tadokoro A.; Sugiura Y.; Nishi M.; Kawata M.; Sakai N.; Matile S.; Futaki S. Direct and Rapid Cytosolic Delivery Using Cell-Penetrating Peptides Mediated by Pyrenebutyrate. ACS Chem. Biol. 2006, 1, 299–303. 10.1021/cb600127m. 17163758

[ref42] Kato T.; Lim B.; Cheng Y.; Pham A.-T.; Maynard J.; Moreau D.; Poblador-Bahamonde A. I.; Sakai N.; Matile S. Cyclic Thiosulfonates for Thiol-Mediated Uptake: Cascade Exchangers, Transporters, Inhibitors. JACS Au 2022, 2, 839–852. 10.1021/jacsau.1c00573. 35557769 PMC9088311

[ref43] Shybeka I.; Maynard J. R. J.; Saidjalolov S.; Moreau D.; Sakai N.; Matile S. Dynamic Covalent Michael Acceptors to Penetrate Cells: Thiol-Mediated Uptake with Tetrel-Centered Exchange Cascades, Assisted by Halogen-Bonding Switches. Angew. Chem., Int. Ed. 2022, 61, e202213433 10.1002/anie.202213433. PMC10098706 36272154

[ref44] Appenzeller-Herzog C.; Ellgaard L. The Human PDI Family: Versatility Packed into a Single Fold. Biochim. Biophys. Acta 2008, 1783, 535–548. 10.1016/j.bbamcr.2007.11.010. 18093543

[ref45] Powell L. E.; Foster P. A. Protein Disulphide Isomerase Inhibition as a Potential Cancer Therapeutic Strategy. Cancer Med. 2021, 10, 2812–2825. 10.1002/cam4.3836. 33742523 PMC8026947

[ref46] Sousa S. F.; Neves R. P. P.; Waheed S. O.; Fernandes P. A.; Ramos M. J. Structural and Mechanistic Aspects of S-S Bonds in the Thioredoxin-like Family of Proteins. Biol. Chem. 2019, 400, 575–587. 10.1515/hsz-2018-0319. 30367780

[ref47] Kozlov G.; Määttänen P.; Thomas D. Y.; Gehring K. A Structural Overview of the PDI Family of Proteins. FEBS J. 2010, 277, 3924–3936. 10.1111/j.1742-4658.2010.07793.x. 20796029

[ref48] Chichiarelli S.; Altieri F.; Paglia G.; Rubini E.; Minacori M.; Eufemi M. ERp57/PDIA3: New Insight. Cell. Mol. Biol. Lett. 2022, 27, 12 10.1186/s11658-022-00315-x. 35109791 PMC8809632

[ref49] Mahmood F.; Xu R.; Awan M. U. N.; Song Y.; Han Q.; Xia X.; Zhang J. PDIA3: Structure, Functions and Its Potential Role in Viral Infections. Biomed. Pharmacother. 2021, 143, 112110 10.1016/j.biopha.2021.112110. 34474345

[ref50] Abegg D.; Gasparini G.; Hoch D. G.; Shuster A.; Bartolami E.; Matile S.; Adibekian A. Strained Cyclic Disulfides Enable Cellular Uptake by Reacting with the Transferrin Receptor. J. Am. Chem. Soc. 2017, 139, 231–238. 10.1021/jacs.6b09643. 28001050

[ref51] Zong L.; Bartolami E.; Abegg D.; Adibekian A.; Sakai N.; Matile S. Epidithiodiketopiperazines: Strain-Promoted Thiol-Mediated Cellular Uptake at the Highest Tension. ACS Cent. Sci. 2017, 3, 449–453. 10.1021/acscentsci.7b00080. 28573207 PMC5445525

[ref52] Cheng Y.; Zong L.; López-Andarias J.; Bartolami E.; Okamoto Y.; Ward T. R.; Sakai N.; Matile S. Cell-Penetrating Dynamic-Covalent Benzopolysulfane Networks. Angew. Chem., Int. Ed. 2019, 58, 9522–9526. 10.1002/anie.201905003. PMC6618005 31168906

[ref53] Cheng Y.; Pham A.-T.; Kato T.; Lim B.; Moreau D.; López-Andarias J.; Zong L.; Sakai N.; Matile S. Inhibitors of Thiol-Mediated Uptake. Chem. Sci. 2021, 12, 626–631. 10.1039/D0SC05447J. PMC8179002 34163793

[ref54] Peraro L.; Deprey K. L.; Moser M. K.; Zou Z.; Ball H. L.; Levine B.; Kritzer J. A. Cell Penetration Profiling Using the Chloroalkane Penetration Assay. J. Am. Chem. Soc. 2018, 140, 11360–11369. 10.1021/jacs.8b06144. 30118219 PMC6205923

[ref55] Ņikitjuka A.; Žalubovskis R. Asparagusic Acid - A Unique Approach toward Effective Cellular Uptake of Therapeutics: Application, Biological Targets, and Chemical Properties. ChemMedChem. 2023, 18, e202300143 10.1002/cmdc.202300143. 37366073

[ref56] Ferreira R. B.; Law M. E.; Jahn S. C.; Davis B. J.; Heldermon C. D.; Reinhard M.; Castellano R. K.; Law B. K. Novel Agents That Downregulate EGFR, HER2, and HER3 in Parallel. Oncotarget 2015, 6, 10445–10459. 10.18632/oncotarget.3398. 25865227 PMC4496366

[ref57] Zhong Y.; Xu Y.; Anslyn E. V. Studies of Reversible Conjugate Additions. Eur. J. Org. Chem. 2013, 2013, 5017–5021. 10.1002/ejoc.201300358.

[ref58] Bravin C.; Duindam N.; Hunter C. A. Artificial Transmembrane Signal Transduction Mediated by Dynamic Covalent Chemistry. Chem. Sci. 2021, 12, 14059–14064. 10.1039/D1SC04741H. 34760189 PMC8565364

[ref59] Cavallo G.; Metrangolo P.; Milani R.; Pilati T.; Priimagi A.; Resnati G.; Terraneo G. The Halogen Bond. Chem. Rev. 2016, 11, 2478–2601. 10.1021/acs.chemrev.5b00484. PMC4768247 26812185

[ref60] Jakka S. R.; Govindaraj V.; Mugesh G. A Single Atom Change Facilitates the Membrane Transport of Green Fluorescent Proteins in Mammalian Cells. Angew. Chem., Int. Ed. 2019, 58, 7713–7717. 10.1002/anie.201902347. 30994954

[ref61] Crooke S. T.; Seth P. P.; Vickers T. A.; Liang X. The Interaction of Phosphorothioate-Containing RNA Targeted Drugs with Proteins Is a Critical Determinant of the Therapeutic Effects of These Agents. J. Am. Chem. Soc. 2020, 142, 14754–14771. 10.1021/jacs.0c04928. 32786803

[ref62] Mammen M.; Choi S.-K.; Whitesides G. M. Polyvalent Interactions in Biological Systems: Implications for Design and Use of Multivalent Ligands and Inhibitors. Angew. Chem., Int. Ed. 1998, 37, 2754–2794. 10.1002/(SICI)1521-3773(19981102)37:20<2754::AID-ANIE2754>3.0.CO;2-3. 29711117

[ref63] Hunter C. A.; Anderson H. L. What Is Cooperativity?. Angew. Chem., Int. Ed. 2009, 48, 7488–7499. 10.1002/anie.200902490. 19746372

[ref64] Macpherson L. J.; Dubin A. E.; Evans M. J.; Marr F.; Schultz P. G.; Cravatt B. F.; Patapoutian A. Noxious Compounds Activate TRPA1 Ion Channels Through Covalent Modification of Cysteines. Nature 2007, 445, 541–545. 10.1038/nature05544. 17237762

[ref65] Lim B.; Cheng Y.; Kato T.; Pham A.-T.; Le Du E.; Mishra A. K.; Grinhagena E.; Moreau D.; Sakai N.; Waser J.; Matile S. Inhibition of Thiol-Mediated Uptake with Irreversible Covalent Inhibitors. Helv. Chim. Acta 2021, 104, e2100085 10.1002/hlca.202100085.

[ref66] Abegg D.; Frei R.; Cerato L.; Hari D. P.; Wang C.; Waser J.; Adibekian A. Proteome-Wide Profiling of Targets of Cysteine Reactive Small Molecules by Using Ethynyl Benziodoxolone Reagents. Angew. Chem., Int. Ed. 2015, 54, 10852–10857. 10.1002/anie.201505641. 26211368

[ref67] Qing Y.; Ionescu S. A.; Pulcu G. S.; Bayley H. Directional Control of a Processive Molecular Hopper. Science 2018, 361, 908–912. 10.1126/science.aat3872. 30166488

[ref68] Lloyd N. C.; Morgan H. W.; Nicholson B. K.; Ronimus R. S. The Composition of Ehrlich’s Salvarsan: Resolution of a Century-Old Debate. Angew. Chem., Int. Ed. 2005, 44, 941–944. 10.1002/anie.200461471. 15624113

[ref69] Popielarski M.; Ponamarczuk H.; Stasiak M.; Watała C.; Świątkowska M. Modifications of Disulfide Bonds in Breast Cancer Cell Migration and Invasiveness. Am. J. Cancer Res. 2019, 9, 1554–1582. 31497343 PMC6727000

[ref70] Aubry S.; Burlina F.; Dupont E.; Delaroche D.; Joliot A.; Lavielle S.; Chassaing G.; Sagan S. Cell-Surface Thiols Affect Cell Entry of Disulfide-Conjugated Peptides. FASEB J. 2009, 23, 2956–2967. 10.1096/fj.08-127563. 19403512

[ref71] Gasparini G.; Bang E.-K.; Molinard G.; Tulumello D. V.; Ward S.; Kelley S. O.; Roux A.; Sakai N.; Matile S. Cellular Uptake of Substrate-Initiated Cell-Penetrating Poly(disulfide)s. J. Am. Chem. Soc. 2014, 136, 6069–6074. 10.1021/ja501581b. 24735462

[ref72] Bouffard J.; Coelho F.; Sakai N.; Matile S. Dynamic Phosphorus: Thiolate Exchange Cascades with Higher Phosphorothioates. Angew. Chem., Int. Ed. 2023, 62, e202313931 10.1002/anie.202313931. 37847524

[ref73] Chuard N.; Poblador-Bahamonde A. I.; Zong L.; Bartolami E.; Hildebrandt J.; Weigand W.; Sakai N.; Matile S. Diselenolane-Mediated Cellular Uptake. Chem. Sci. 2018, 9, 1860–1866. 10.1039/C7SC05151D. 29675232 PMC5892345

[ref74] Lang W.; Tan W.; Zhou B.; Zhuang Y.; Zhang B.; Jiang L.; Yao S. Q.; Ge J. Mitochondria-Targeted Gene Silencing Facilitated by Mito-CPDs. Chem.—Eur. J. 2023, 29, e202204021 10.1002/chem.202204021. 36806226

[ref75] Turano C.; Gaucci E.; Grillo C.; Chichiarelli S. ERp57/GRP58: A Protein with Multiple Functions. Cell. Mol. Biol. Lett. 2011, 16, 539 10.2478/s11658-011-0022-z. 21837552 PMC6275603

[ref76] Chen J.; Olivares-Navarrete R.; Wang Y.; Herman T. R.; Boyan B. D.; Schwartz Z. Protein-Disulfide Isomerase-Associated 3 (PDIA3) Mediates the Membrane Response to 1,25-Dihydroxyvitamin D3 in Osteoblasts. J. Biol. Chem. 2010, 285, 37041–37050. 10.1074/jbc.M110.157115. 20843786 PMC2978632

[ref77] Leduc A.-M.; Trent J. O.; Wittliff J. L.; Bramlett K. S.; Briggs S. L.; Chirgadze N. Y.; Wang Y.; Burris T. P.; Spatola A. F. Helix-Stabilized Cyclic Peptides as Selective Inhibitors of Steroid Receptor-Coactivator Interactions. Proc. Natl. Acad. Sci. U. S. A. 2003, 100, 11273–11278. 10.1073/pnas.1934759100. 13679575 PMC208747

[ref78] Lee M. K.; Kim H. K.; Lee T. Y.; Hahm K.-S.; Kim K. L. Structure-Activity Relationships of Anti-HIV-1 Peptides with Disulfide Linkage between D- and L-Cysteine at Positions i and I+3, Respectively, Derived from HIV-1 Gp41 C-Peptide. Exp. Mol. Med. 2006, 38, 18–26. 10.1038/emm.2006.3. 16520549

[ref79] Iqbalsyah T. M.; Moutevelis E.; Warwicker J.; Errington N.; Doig A. J. The CXXC Motif at the N Terminus of an α-Helical Peptide. Protein Sci. 2006, 15, 1945–1950. 10.1110/ps.062271506. 16877711 PMC2242585

[ref80] Quan S.; Schneider I.; Pan J.; Von Hacht A.; Bardwell J. C. A. The CXXC Motif Is More than a Redox Rheostat. J. Biol. Chem. 2007, 282, 28823–28833. 10.1074/jbc.M705291200. 17675287

[ref81] Ge J.; Zhang C.-J.; Li L.; Chong L. M.; Wu X.; Hao P.; Sze S. K.; Yao S. Q. Small Molecule Probe Suitable for *In Situ* Profiling and Inhibition of Protein Disulfide Isomerase. ACS Chem. Biol. 2013, 8, 2577–2585. 10.1021/cb4002602. 24070012

[ref82] Hoffstrom B. G.; Kaplan A.; Letso R.; Schmid R. S.; Turmel G. J.; Lo D. C.; Stockwell B. R. Inhibitors of Protein Disulfide Isomerase Suppress Apoptosis Induced by Misfolded Proteins. Nat. Chem. Biol. 2010, 6, 900–906. 10.1038/nchembio.467. 21079601 PMC3018711

[ref83] Chamberlain N.; Korwin-Mihavics B. R.; Nakada E. M.; Bruno S. R.; Heppner D. E.; Chapman D. G.; Hoffman S. M.; van der Vliet A.; Suratt B. T.; Dienz O.; et al. Lung Epithelial Protein Disulfide Isomerase A3 (PDIA3) Plays an Important Role in Influenza Infection, Inflammation, and Airway Mechanics. Redox Biol. 2019, 22, 101129 10.1016/j.redox.2019.101129. 30735910 PMC6365984

[ref84] Kaplan A.; Gaschler M. M.; Dunn D. E.; Colligan R.; Brown L. M.; Palmer A. G.; Lo D. C.; Stockwell B. R. Small Molecule-Induced Oxidation of Protein Disulfide Isomerase Is Neuroprotective. Proc. Natl. Acad. Sci. U. S. A. 2015, 112, E2245-E2252 10.1073/pnas.1500439112. 25848045 PMC4418888

[ref85] Xu S.; Sankar S.; Neamati N. Protein Disulfide Isomerase: A Promising Target for Cancer Therapy. Drug Discovery Today 2014, 19, 222–240. 10.1016/j.drudis.2013.10.017. 24184531

[ref86] Xu S.; Butkevich A. N.; Yamada R.; Zhou Y.; Debnath B.; Duncan R.; Zandi E.; Petasis N. A.; Neamati N. Discovery of an Orally Active Small-Molecule Irreversible Inhibitor of Protein Disulfide Isomerase for Ovarian Cancer Treatment. Proc. Natl. Acad. Sci. U. S. A. 2012, 109, 16348–16353. 10.1073/pnas.1205226109. 22988091 PMC3479552

[ref87] Powell L. E.; Foster P. A. Protein Disulphide Isomerase Inhibition as a Potential Cancer Therapeutic Strategy. Cancer Med. 2021, 10, 2812–2825. 10.1002/cam4.3836. 33742523 PMC8026947

[ref88] Lin L.; Gopal S.; Sharda A.; Passam F.; Bowley S. R.; Stopa J.; Xue G.; Yuan C.; Furie B. C.; Flaumenhaft R.; et al. Quercetin-3-Rutinoside Inhibits Protein Disulfide Isomerase by Binding to Its B′x Domain. J. Biol. Chem. 2015, 290, 23543–23552. 10.1074/jbc.M115.666180. 26240139 PMC4583019

[ref89] Jasuja R.; Passam F. H.; Kennedy D. R.; Kim S. H.; Van Hessem L.; Lin L.; Bowley S. R.; Joshi S. S.; Dilks J. R.; Furie B.; et al. Protein Disulfide Isomerase Inhibitors Constitute a New Class of Antithrombotic Agents. J. Clin. Invest. 2012, 122, 2104–2113. 10.1172/JCI61228. 22565308 PMC3366406

[ref90] Cheff D. M.; Huang C.; Scholzen K. C.; Gencheva R.; Ronzetti M. H.; Cheng Q.; Hall M. D.; Arnér E. S. J. The Ferroptosis Inducing Compounds RSL3 and ML162 Are Not Direct Inhibitors of GPX4 but of TXNRD1. Redox Biol. 2023, 62, 102703 10.1016/j.redox.2023.102703. 37087975 PMC10149367

[ref91] Dixon S. J.; Lemberg K. M.; Lamprecht M. R.; Skouta R.; Zaitsev E. M.; Gleason C. E.; Patel D. N.; Bauer A. J.; Cantley A. M.; Yang W. S.; et al. Ferroptosis: An Iron-Dependent Form of Nonapoptotic Cell Death. Cell 2012, 149, 1060–1072. 10.1016/j.cell.2012.03.042. 22632970 PMC3367386

[ref92] Hii C. S.; Ferrante A. The Non-Genomic Actions of Vitamin D. Nutrients 2016, 8, 135 10.3390/nu8030135. 26950144 PMC4808864

[ref93] Wolfenden R.; Snider M. J. The Depth of Chemical Time and the Power of Enzymes as Catalysts. Acc. Chem. Res. 2001, 34, 938–945. 10.1021/ar000058i. 11747411

