# Supplementary material for: Inclusive Pattern Generation Protocols to Decode Thiol-Mediated Uptake
Source: ACS Cent Sci. 2024 Apr 17;10(5):1033–43. doi: 10.1021/acscentsci.3c01601 (PMC11117725; doi:10.1021/acscentsci.3c01601)
Supplement: Supplementary file 1 — oc3c01601_si_001.pdf [file oc3c01601_si_001.pdf]

Supporting Information

Inclusive Pattern Generation Protocols to Decode Thiol-Mediated Uptake

Saidbakhrom Saidjalolov,[§,‡] Filipe Coelho,[§,‡] Vincent Mercier,[†] Dimitri Moreau,[†] and Stefan Matile*,[§]

[§]Department of Organic Chemistry, University of Geneva, CH-1211 Geneva, Switzerland

[†]Department of Biochemistry, University of Geneva, CH-1211 Geneva, Switzerland

*E-mail: stefan.matile@unige.ch

[‡]These two authors contributed equally

Table of Contents

1. Materials and Methods S5

2. Compounds S7

    2.1. Inhibitors S7

    2.2. Transporters S8

3. Quenching Factors S9

4. Cell Culture S12

5. Automated High-Content High-Throughput (AHCHT) Imaging-Based Cellular Uptake Assay for Fl-CAX Transporters S13

    5.1. General Experimental Procedure S13

    5.2. Data Analysis S13

    5.3. Uptake of Fl-CAXs in Various Cell Lines S15

6. AHCHT Inhibitor Screening S21

    6.1. General Experimental Procedure S21

    6.2. Data Analysis S22

    6.3. Inhibitor Screening in HK Cells S23

        6.3.1. Fl-BPS Transporter S23

        6.3.2. Fl-ETP Transporter S29

        6.3.3. Fl-AspA Transporter S34

        6.3.4. Fl-CTO Transporter S40

        6.3.5. Fl-MAC Transporter S45

        6.3.6. OPS-Cy5 Transporter S45

    6.4. Inhibitor Screening in Various Cell Lines S46

7. AHCHT Screening for OPS-Cy5 S54

    7.1 General Experimental Procedure for OPS-Cy5 Uptake S54

7.2 Data Analysis S55

8. Uptake and Inhibition Studies in Integrin Knockdown HK Cells S56

8.1. Knockdown Procedure S56

8.2. Protein Knockdown Quantification by Immunofluorescence S56

8.3. Cellular Uptake in Integrin Knocked-Down Cells S58

8.3.1. Fl-CAXs S58

8.3.2. OPS-Cy5 S63

8.4. Inhibitor Screening in Knocked-down Cells S64

8.4.1. Fl-BPS Transporter S64

8.4.2. Fl-ETP Transporter S67

9. Effects of PDI Inhibitors S75

9.1. LC-MS Analysis of Transporters in Presence of PDI Inhibitors S75

9.2. Cellular Uptake in the Presence of PDI Inhibitors S77

9.2.1. Effects on Fl-BPS Uptake in HK Cells S77

9.2.2. Effects on Fl-ETP Uptake in HK Cells S80

9.2.3. Effects on Fl-AspA Uptake in HK Cells S83

9.2.4. Effects on Fl-CTO Uptake in HK Cells S86

9.2.5. Effects on Fl-MAC Uptake in HK Cells S89

9.2.6. Effects on OPS-Cy5 Uptake in HK Cells S92

9.2.7. Fl-BPS Uptake in Different Cell Lines S94

9.3. Inhibitor Screening in the Presence of PDI Inhibitors S95

9.3.1. Fl-BPS Transporter S96

9.3.2. FI-ETP Transporter S102

10. Co-Localization of OPS-Cy5 and Transferrin CF488A Conjugate in HK Cells S106

11. Data Analysis of OPS-Cy5 and Transferrin-CF448A Co-Localization S108

12. Supporting References S110

1. Materials and Methods

As mentioned in reference S1, phosphate-buffered saline (PBS, pH = 7.4), FDMEM (4.5 g/L D-glucose, without phenol red) medium, Penicillin- Streptomycin, RNAiMAX, Fetal Bovine Serum, TrypLE Express Enzyme and V96-MicroWell plate were obtained from Thermo Fisher Scientific. μ-Plate 96-Well was obtained from Ibidi. Hoechst 33342 (10 mg/mL solution in water) was obtained from Invitrogen by Thermo Fisher Scientific. DTNB, 16F16, PACMA-31 and Rutin were obtained from Sigma-Aldrich. LOC14 was purchased from MedChemExpress. OPS (Cy5-*A*G*G*T*C*C*C*C*A*T*A*C*A*C*C*G*A*C, * stands for O→S substitution) was purchased from Merck KGaA and used as received. The siITGB1, siITGB3, siITGB5 (NCBI Gene ID 3688, 3690, 3693, respectively) and non-target were purchased from siTOOLs Biotech. The lipofectamine RNAiMAX was purchased from Invitrogen (13778-030). The reduced serum medium Opti-MEM was obtained from Gibco. The anti-integrin β1 and β3 antibodies were obtained from abcam (mouse monoclonal, ab30394 and ab24693 respectively). The anti-integrin β5 antibody was purchased from Invitrogen (14-0497-82). The secondary antibody was obtained from Jackson Immuno LTD (715-605-150). Imaging was performed using an IXM-C automated microscope from ImageXpress equipped with a Lumencor Aura III with 5 independent selectable solid-state light sources, bandpass filters and 5 objectives (4X to 60X). Washing steps were performed using a plate washer Biotek EL406®. LC-MS analyses were performed on Advion Avant® UHPLC system with Advion Expression® CMS in ESI mode. All mass data are reported as mass-per-charge ratio m/z. UV-Vis spectra were recorded on a JASCO V-650 spectrophotometer equipped with a stirrer and a temperature controller (20 ºC) and are reported as maximal absorption wavelength λ in nm (extinction coefficient ε in M^{-1} cm^{-1}). Fluorescent measurements were performed on a FluoroMax-4 (Horiba Scientific).

Abbreviations. AsC: 2-(4-Aminophenyl)-1,3,2-dithiarsinane-5-carboxylic acid; AspA: Asparagusic acid; AHCHT: Automated high-content high-throughput; BiC: 2-Chloro-1,3,2-dithiabismepane-5,6-diol; BPS: Benzopolysulfane; BSA: Bovine serum albumin; CAX: Covalent exchangers; CTO: Cyclic thiosulfonate; dMAC: Double Michael acceptor; DMEM: Dulbecco's Modified Eagle Medium; DTNB: 5,5'-Dithiobis-(2-nitrobenzoic acid); DTT: Dithiothreitol; EBS: Ebselen analogue; EBX: Ethynylbenziodoxolone; ETP: Epidithiodiketopiperazine; FBS: Fetal bovine serum; FDMEM: FluoroBrite DMEM; FITC: Fluorescein isothiocyanate; HeLa: Henrietta Lacks; HK: HeLa Kyoto; IC_{50}: Half maximal inhibitory concentration; MAC: Michael acceptor; MIC: Minimum inhibitory concentration; OPS: Oligonucleotide phosphorothioate; PBS: Phosphate-buffered saline; PFA: Paraformaldehyde; PDI: Protein disulfide isomerase; PI: Propidium iodide; rt: Room temperature; RV: Relative viability; SD: Standard deviation; SDCM: Spinning disk confocal microscopy; SEM: Standard error of mean; SS: Super-spice; TMU: Thiol-mediated uptake; TRIS buffer: Tris(hydroxymethyl)aminoethane buffer; WI: Water immersion; WT: Wild type.

2. Compounds

2.1. Inhibitors

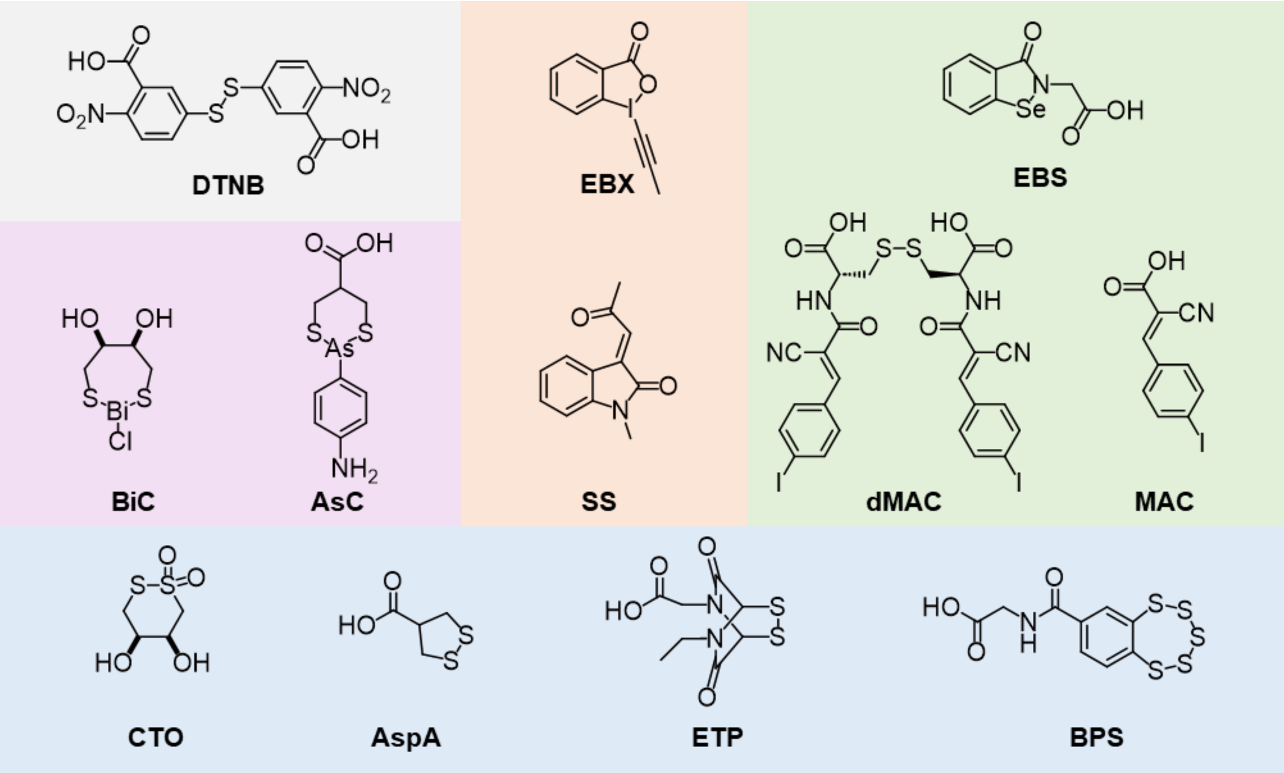

Figure S1. Structures of inhibitors. Color code: light grey: control; light orange: irreversible inhibitors; light green: reversible inhibitors; light purple: pnictogen-centered inhibitors; blue: CAX inhibitors.

EBX was synthesized according to procedures described in ref. S2.

EBS was synthesized according to procedures described in ref. S3.

AsC and **BiC** were synthesized according to procedures described in ref. S4.

SS was synthesized according to procedures described in ref. S5.

MAC and **dMAC** were synthesized according to procedures described in ref. S1.

CTO was synthesized according to procedures described in ref. S6.

AspA was synthesized according to procedures described in ref. S7.

ETP and **BPS** were synthesized according to procedures described in ref. S8.

2.2. Transporters

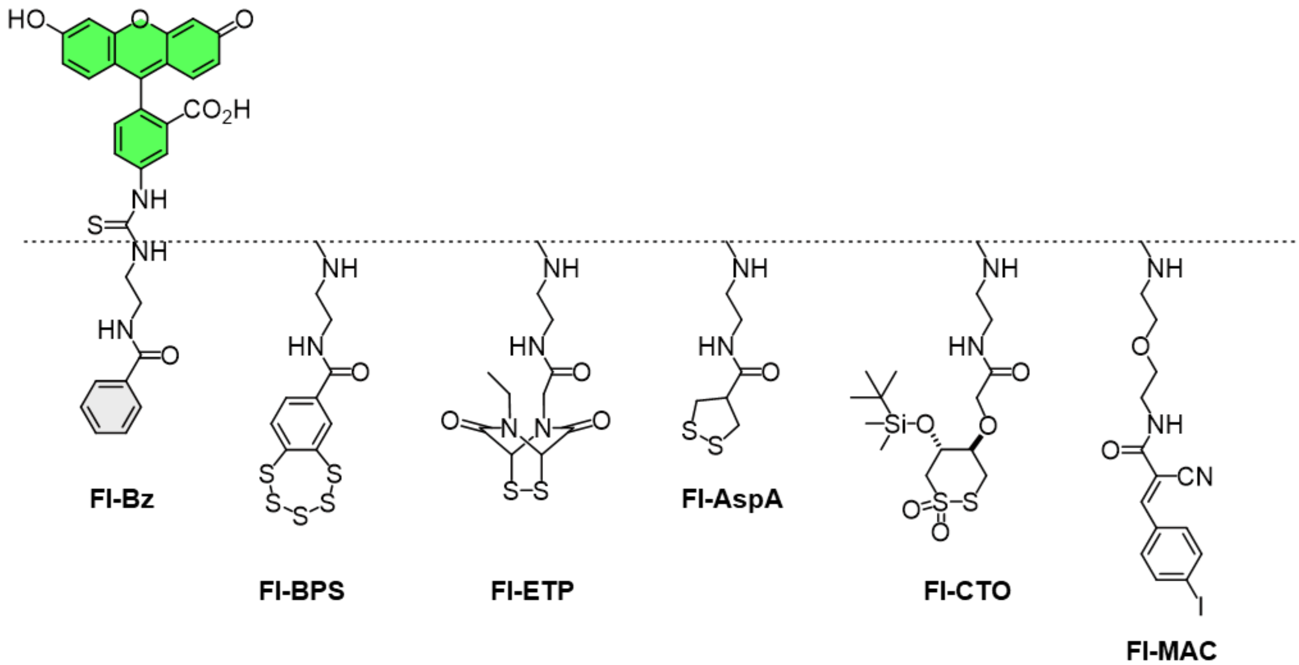

Figure S2. Structure of negative control (**Fl-Bz**) and CAX-transporters bearing a fluorescein (**Fl-CAX**).

Fl-Bz and **Fl-BPS** were synthesized according to procedures described in ref. S9.

Fl-ETP was synthesized according to procedures described in ref. S10.

Fl-AspA was synthesized according to procedures described in ref. S7.

Fl-CTO was synthesized according to procedures described in ref. S6.

Fl-MAC was synthesized according to procedures described in ref. S1.

3. Quenching Factors

Quenching factors were determined following the procedure described in reference S9. Briefly, stock solutions of transporters **Fl-Bz**, **Fl-AspA**, **Fl-ETP**, **Fl-BPS**, **Fl-CTO** and **Fl-MAC** (10 mM in DMSO) were diluted to give 1 µM solutions in PBS buffer. The fluorescence emission spectra were then recorded from 498 nm to 600 nm upon excitation at 488 nm (slits 1.5 nm). Quenching factors of closed CAX were calculated by first dividing the value of emission intensity at 512 nm by the absorbance at 494 nm, and then dividing the obtained value of **Fl-AspA** by that of others. The results are reported in Figure S3 and Table S1. The experiment was done in independent duplicates.

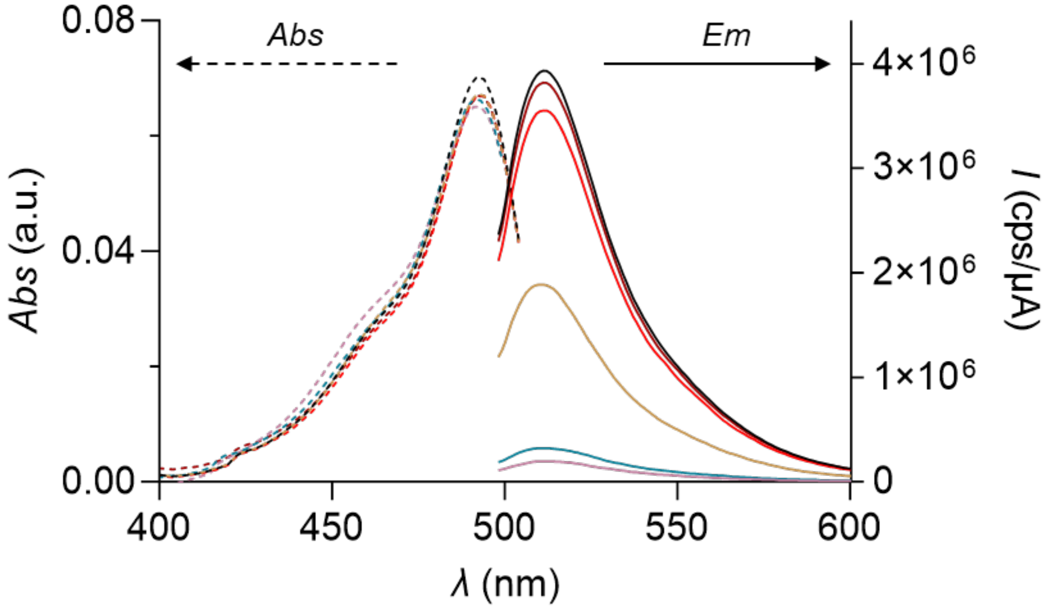

Figure S3. Absorption (dashed) and emission (solid) spectra of **Fl-Bz** (black), **Fl-AspA** (gold), **Fl-ETP** (red), **Fl-BPS** (light purple), **Fl-CTO** (bordeaux), and **Fl-MAC** (teal) in PBS buffer upon excitation at 488 nm.

To obtain the quenching factor under reducing conditions, solutions of fluorescent compounds (10 µL, 100 µM in PBS buffer) were mixed with DTT (10 µL, 2 mM in PBS buffer) for 30 min before diluting to 1 µM in PBS buffer. The fluorescence emission was then recorded and normalized against the control in the same manner as described above. The results are reported in Figure S4 and Table S1. The experiment was done in independent duplicates.

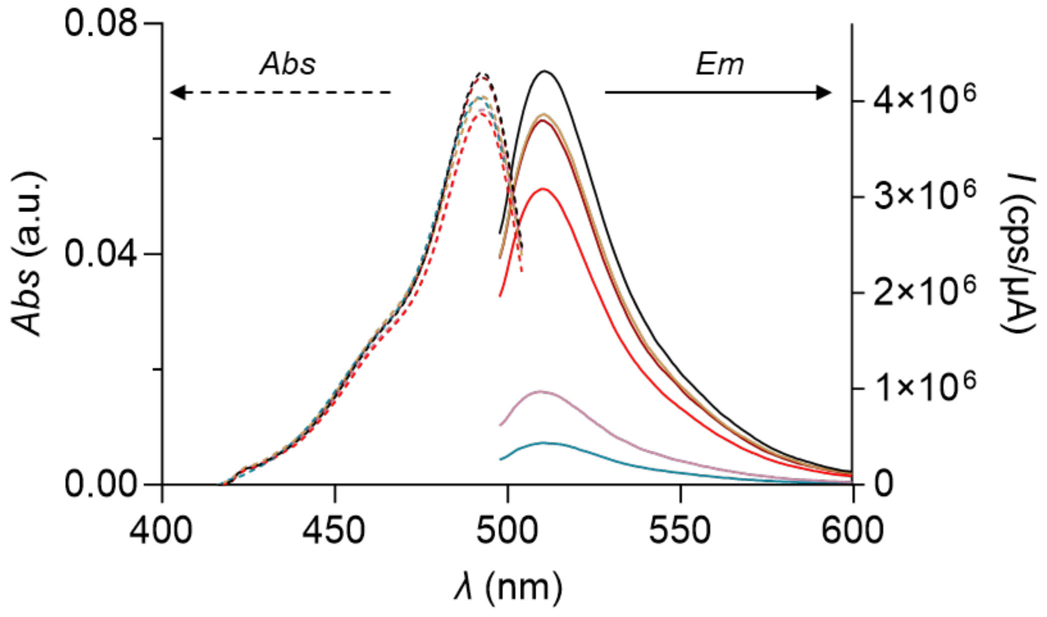

Figure S4. Absorption (dashed) and emission (solid) spectra of **Fl-Bz** (black), **Fl-AspA** (gold), **Fl-ETP** (red), **Fl-BPS** (light purple), **Fl-CTO** (bordeaux), and **Fl-MAC** (teal) in PBS buffer upon excitation at 488 nm.

Table S1. Open and closed fluorescent quenching correction factor (q_C) ± SEM.

Entry	Compound	I_{em} (closed)[a]	I_{em} (open)[a]	Abs[b]	$q_{C\text{-closed}}$	$\boldsymbol{q_C}$
1	**Fl-Bz**	3.9×10^6	4.3×10^6	0.070	0.5	0.7
2	**Fl-AspA**	1.9×10^6	3.9×10^6	0.069	1.0 ± 0.1	1.0 ± 0.2
3	**Fl-ETP**	3.5×10^6	3.1×10^6	0.068	0.5	0.9 ± 0.1
4	**Fl-BPS**	1.9×10^5	9.6×10^5	0.067	9.0 ± 0.4	3.0 ± 0.2
5	**Fl-CTO**	3.8×10^6	3.8×10^6	0.069	0.5	0.9 ± 0.1
6	**Fl-MAC**	3.2×10^5	4.3×10^5	0.067	6.0 ± 0.2	6.0 ± 2.0

[a]Emission intensity at λ_{em} = 512 nm. [b]Absorbance at 494 nm.

4. Cell Culture

Human cervical cancer-derived HeLa Kyoto, human breast cancer-derived MCF-7 and MDA-MB-231, human retinal pigment epithelial-1 (RPE-1) and human epidermoid carcinoma (A-431) cells were cultured in complete FDMEM (GlutaMAX, 4.5 g/L D-glucose) medium, which contains 10% fetal bovine serum (FBS) and 1% Penicillin/Streptomycin (PS). The cells were grown under 5% CO_2 humidified atmosphere at 37 °C on a 75 cm^3 tissue culture flask (TPD Corporation). Cells were harvested by treatment with 3 mL of phenol-red free TrypLE Express, followed by the addition of 10 mL of complete FDMEM (GlutaMAX, 4.5 g/L D-glucose) medium at 37 °C. The cells were spun down at 1500 g for 3 min, re-suspended in complete FDMEM (GlutaMAX, 4.5 g/L D-glucose) medium, and plated according to the concentration needed. For uptake or inhibition experiments, the cells were seeded in a μ-Plate 96-well Black ibiTreat sterile at 12 000 cells/well (HK, RPE-1, A-431) or 18 000 cells/well (MCF-7, MDA-MB-231) in complete FDMEM and left incubating under 5% CO_2 humidified atmosphere at 37 °C overnight.

5. Automated High-Content High-Throughput (AHCHT) Imaging-Based Cellular Uptake Assay for **Fl-CAX** Transporters

5.1. General Experimental Procedure

Cells were prepared in a 96 well plate as described in section 4, then medium was removed, and cells were washed with PBS (3 × 3 mL/well) followed by fresh FDMEM serum-free medium (4 × 100 μL/well) using a plate washer (Biotek EL406®), and kept in a 100 μL of the latter medium. The solution of **Fl-CAX** (10 mM, DMSO) was diluted in FDMEM to give a solution at 3x final concentration, of which 50 μL was added to the well resulting in a final volume of 150 μL per well. The cells were incubated under 5% CO_2 humidified atmosphere at 37 °C for the indicated time (30 – 240 minutes). Afterward, to remove the excess of fluorescent transporter, the cells were washed with PBS and the medium was exchanged with FDMEM keeping a final volume of 100 μL/well, and a solution of Hoechst 33342 (100 μg/mL) and PI (10 μg/mL) in PBS (15 μL/well) was added. After 10 min of incubation under 5% CO_2 humidified atmosphere at 37 °C, cells were washed with PBS (3 × 3 mL/well) and kept in FDMEM (100 μL/well) for live cell imaging. The distribution of fluorescent signals was captured on a IXM-C automated microscope with three channels, blue for Hoechst 33342 (377/50 nm excitation filter; 477/60 nm emission filter), green for **Fl-CAX** transporter (475/34 nm excitation filter; 536/40 nm emission filter) and red for PI (531/40 nm excitation filter; 593/40 nm emission filter). The rest of the parameters were adjusted according to the nature of the experiment. Duplicates were performed for each condition.

5.2. Data Analysis

Resulting images were automatically analyzed and quantified using a protocol similar to that in reference S8. Briefly, the nuclei and cell bodies were segmented using the blue channel image (Hoechst 33342). Dividing and dying cells were detected based on their shape factor (round shape, > 0.8) and filtered out. Dead cells were also filtered out using the PI channel. Bright aggregates resulting from transporter precipitation were segmented and filtered out based on the maximum signal intensity

and their size. The resulting objects were then grown and all cells in the vicinity were removed from the analysis. All the cells touching the border of the image were removed to prevent inaccurate quantification in the final mask (Figure S5). Finally, fluorescence of the green channel was quantified as the average fluorescence intensity per pixels I_{cell} in live cells (pink mask) minus the average intensity I_{bg} of the background (blue) to give average fluorescence intensity in cells I_{CAX}. Relative cell viability (*RV*) was calculated as the count of Hoechst 33342 stained cells minus the count of PI-positive cells divided by the count of Hoechst 33342 stained cells in absence of inhibitor.

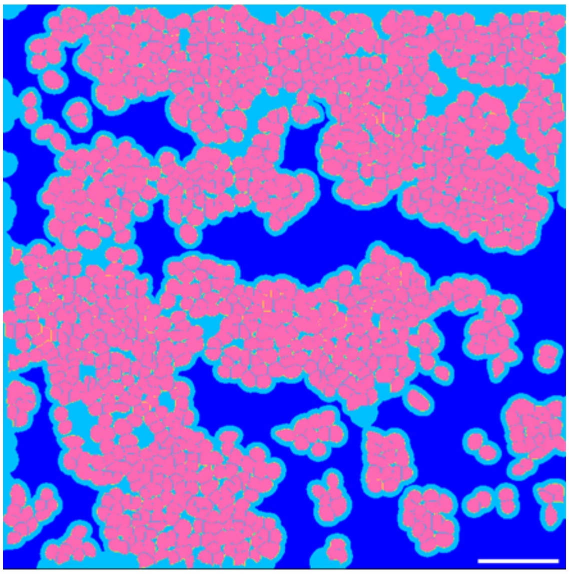

Figure S5. Final mask: pink – cells used for quantification; cyan –small inter-cellular space; dark blue – background. Scale bar 200 µm.

5.3. Uptake of Fl-CAXs in Various Cell Lines

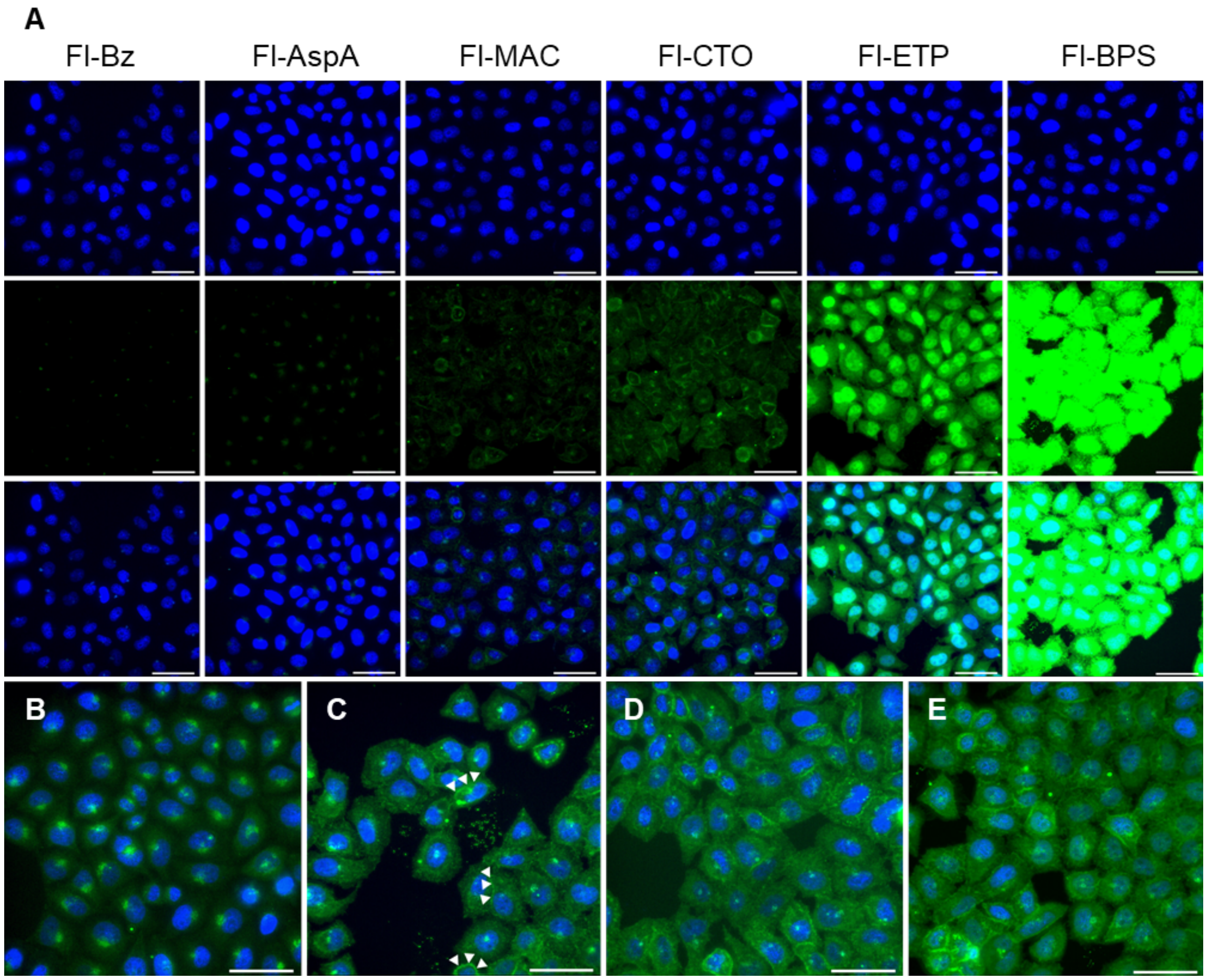

Figure S6. A) SDCM images (60X WI) of HK cells after incubation for 1 h with negative control (**Fl-Bz**) and various **Fl-CAXs** (10 µM, green) (top, blue channel; middle, green channel; bottom, merge), using the same brightness settings. B–E) The brightness-adjusted images of B) **Fl-AspA**, C) **Fl-CTO** with highlighted membrane staining (triangles), D) **Fl-MAC**, and E) **Fl-BPS**. Scale bars 50 µm.

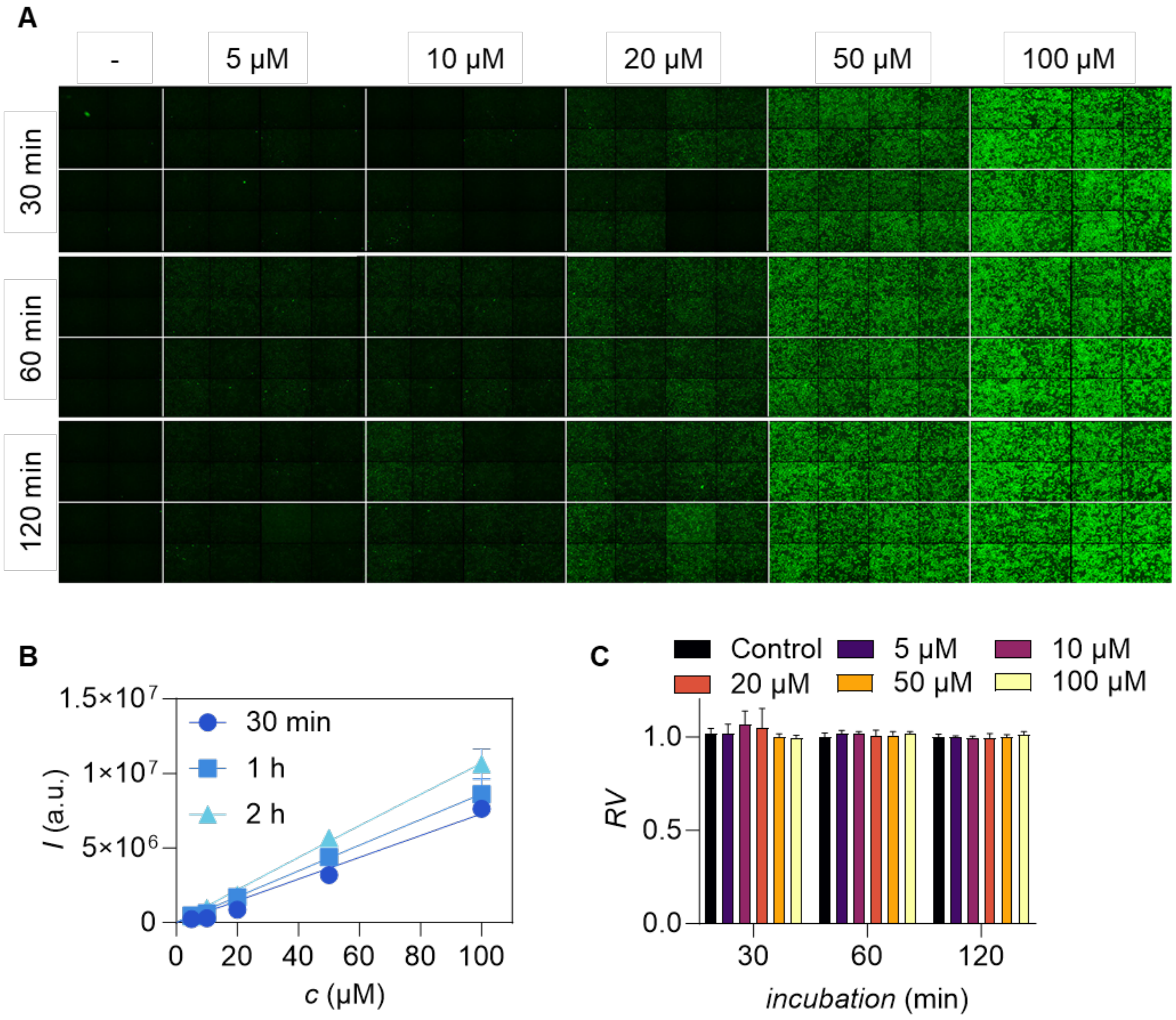

Figure S7. A) SDCM images (10X, widefield, green channel) of HK cells in the 96-well plate after 30, 60, or 120 min incubation with 0 to 100 µM of **Fl-AspA**. B) Fluorescence intensity (*I*) ± SEM and C) relative cell viability (*RV*) ± SEM in HK cells after 30 to 120 minutes of incubation with 5 to 100 µM of **Fl-AspA**.

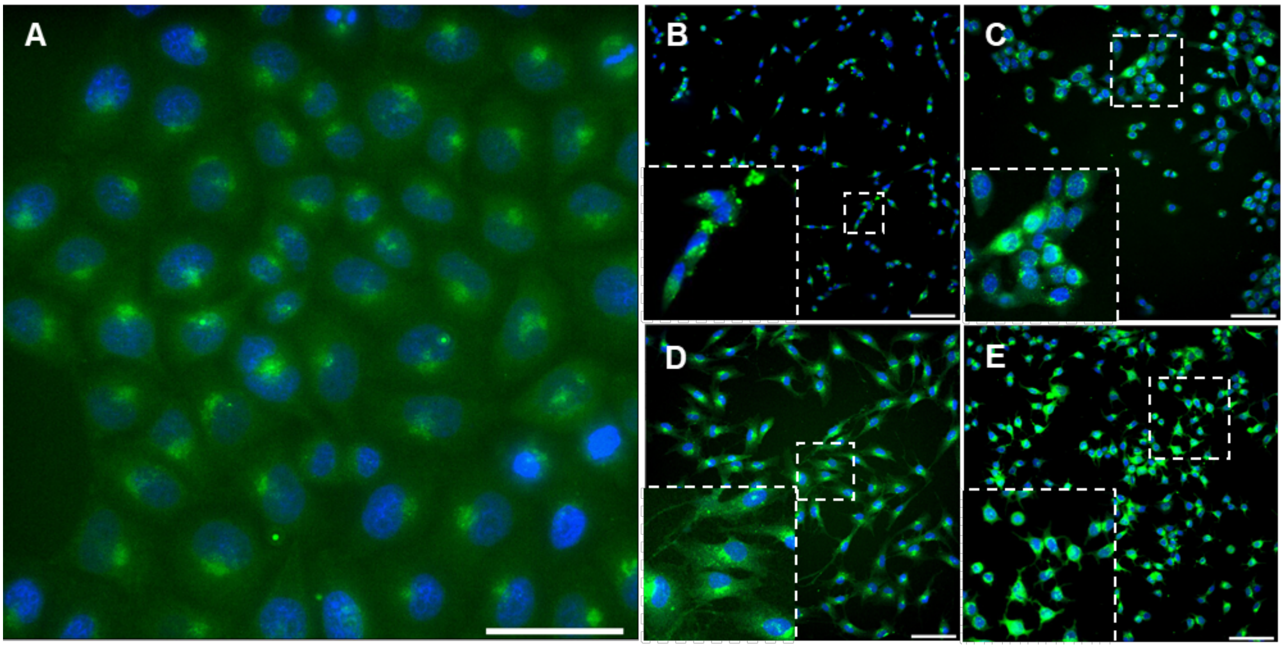

Figure S8. SDCM images (60X WI and 20X WI) of A) HK, B) MDA-MB-231, C) A-431, D) RPE-1 and E) MCF-7 after incubation with **Fl-AspA** (10 µM, green) for 1 h (blue: Hoechst 33342, nuclei; scale bar 50 µm for A and 100 µm for B, C, D and E).

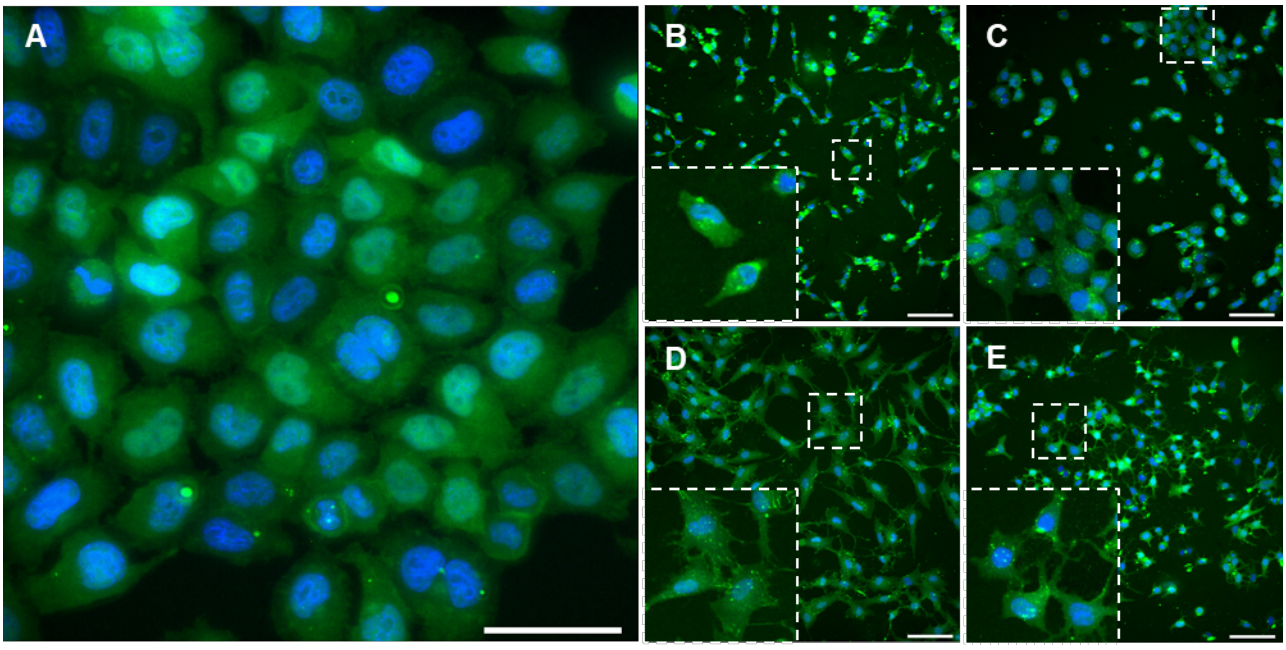

Figure S9. SDCM images (60X WI and 20X WI) of A) HK, B) MDA-MB-231, C) A-431, D) RPE-1 and E) MCF-7 after incubation with **Fl-ETP** (10 µM, green) for 1 h (blue: Hoechst 33342, nuclei; scale bar 50 µm for A and 100 µm for B, C, D and E).

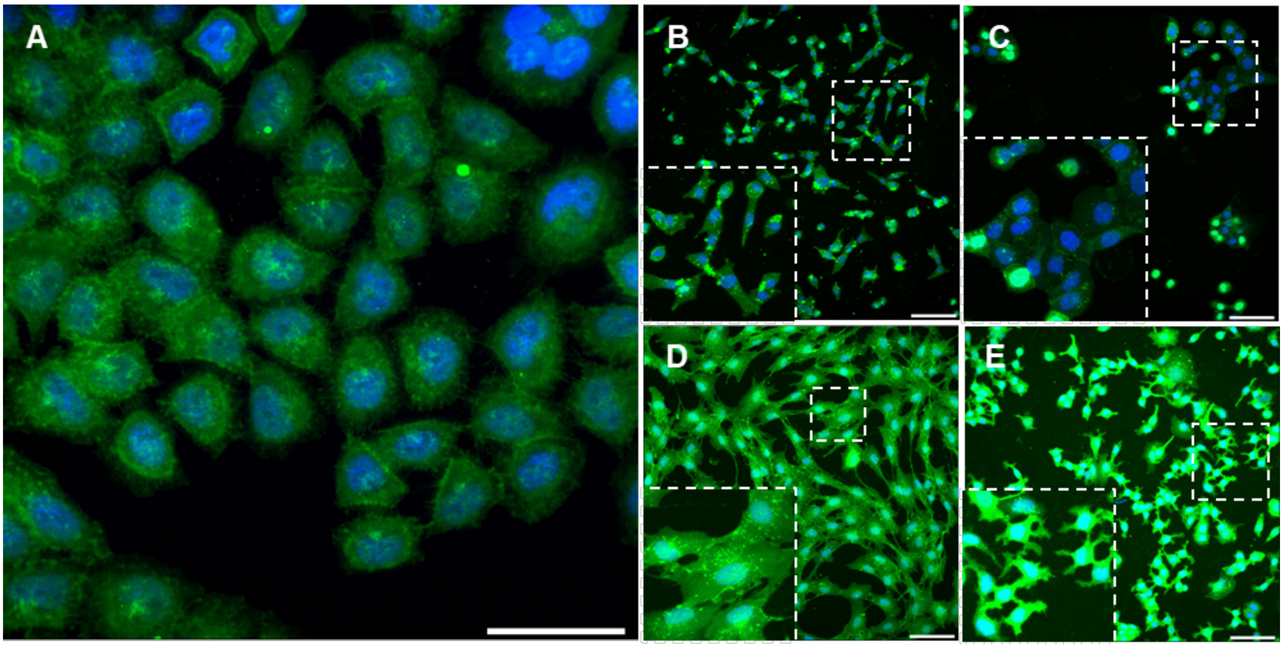

Figure S10. SDCM images (60X WI and 20X WI) of A) HK, B) MDA-MB-231, C) A-431, D) RPE-1 and E) MCF-7 after incubation with **Fl-BPS** (10 µM, green) for 1 h (blue: Hoechst 33342, nuclei; scale bar 50 µm for A and 100 µm for B, C, D and E).

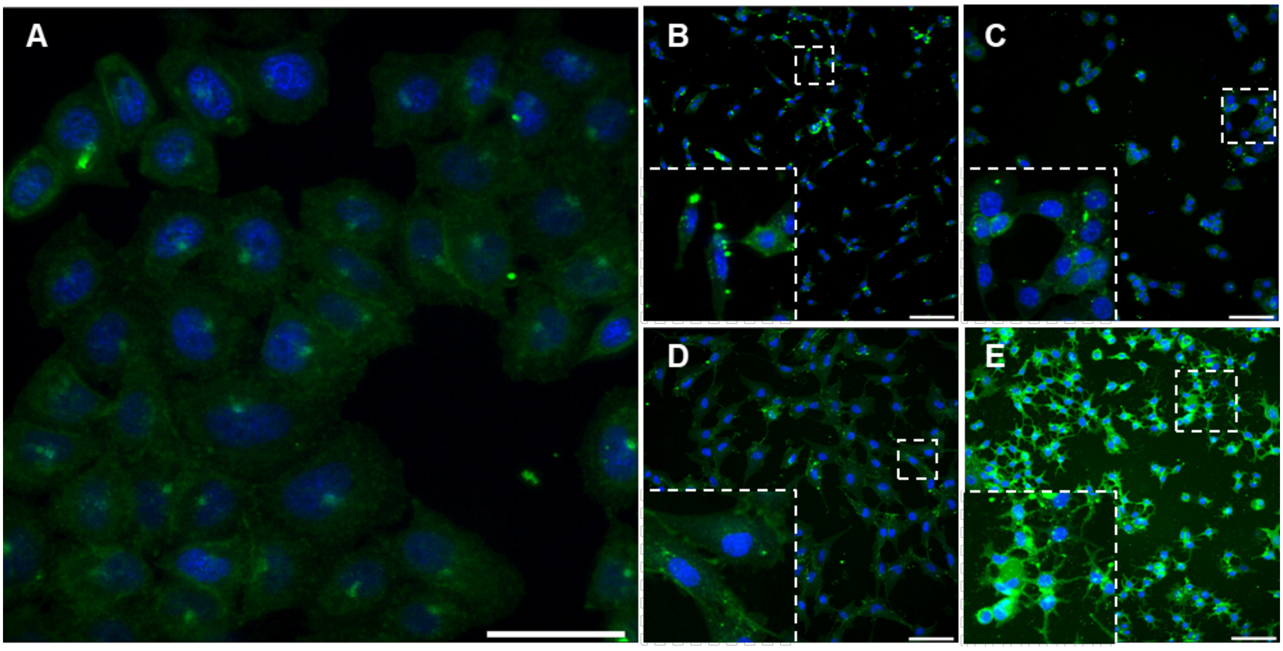

Figure S11. SDCM images (60X WI and 20X WI) of A) HK, B) MDA-MB-231, C) A-431, D) RPE-1 and E) MCF-7 after incubation with **Fl-CTO** (10 µM, green) for 1 h (blue: Hoechst 33342, nuclei; scale bar 50 µm for A and 100 µm for B, C, D and E).

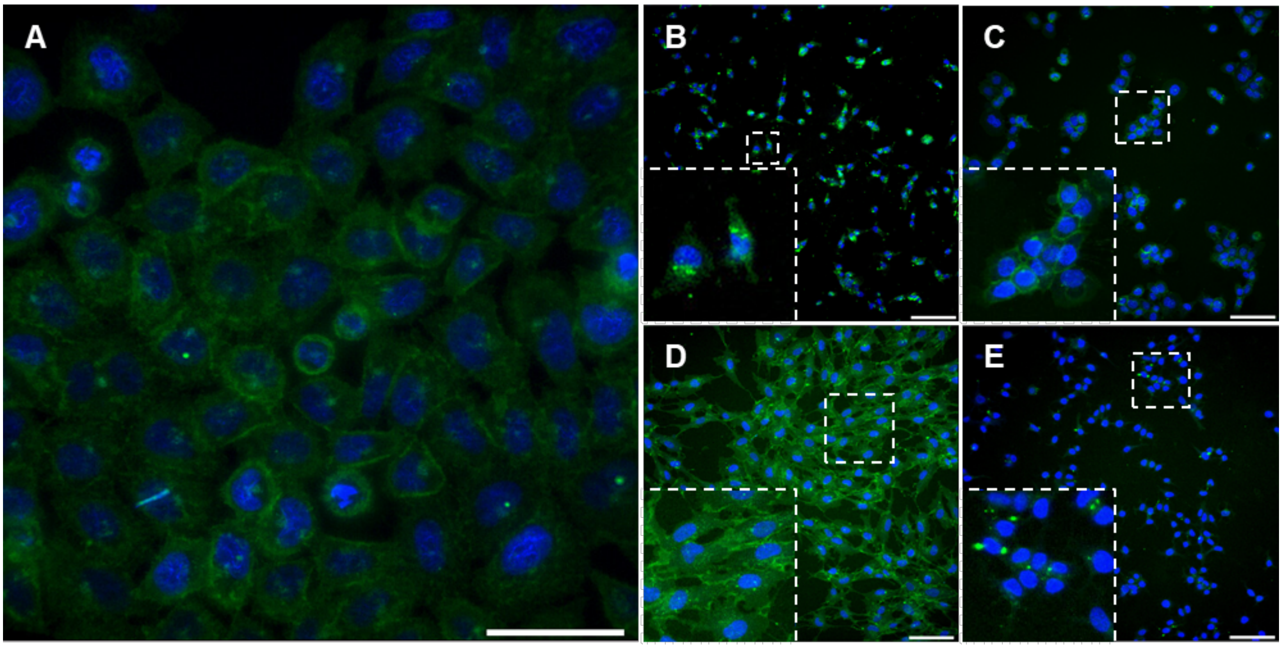

Figure S12. SDCM images (60X WI and 20X WI) showing fluorescence intensity (green) of A) HK, B) MDA-MB-231, C) A-431, D) RPE-1 and E) MCF-7 after incubation with **Fl-MAC** (10 µM) for 1 h (blue: Hoechst 33342, nuclei; scale bar 50 µm for A and 100 µm for B, C, D and E).

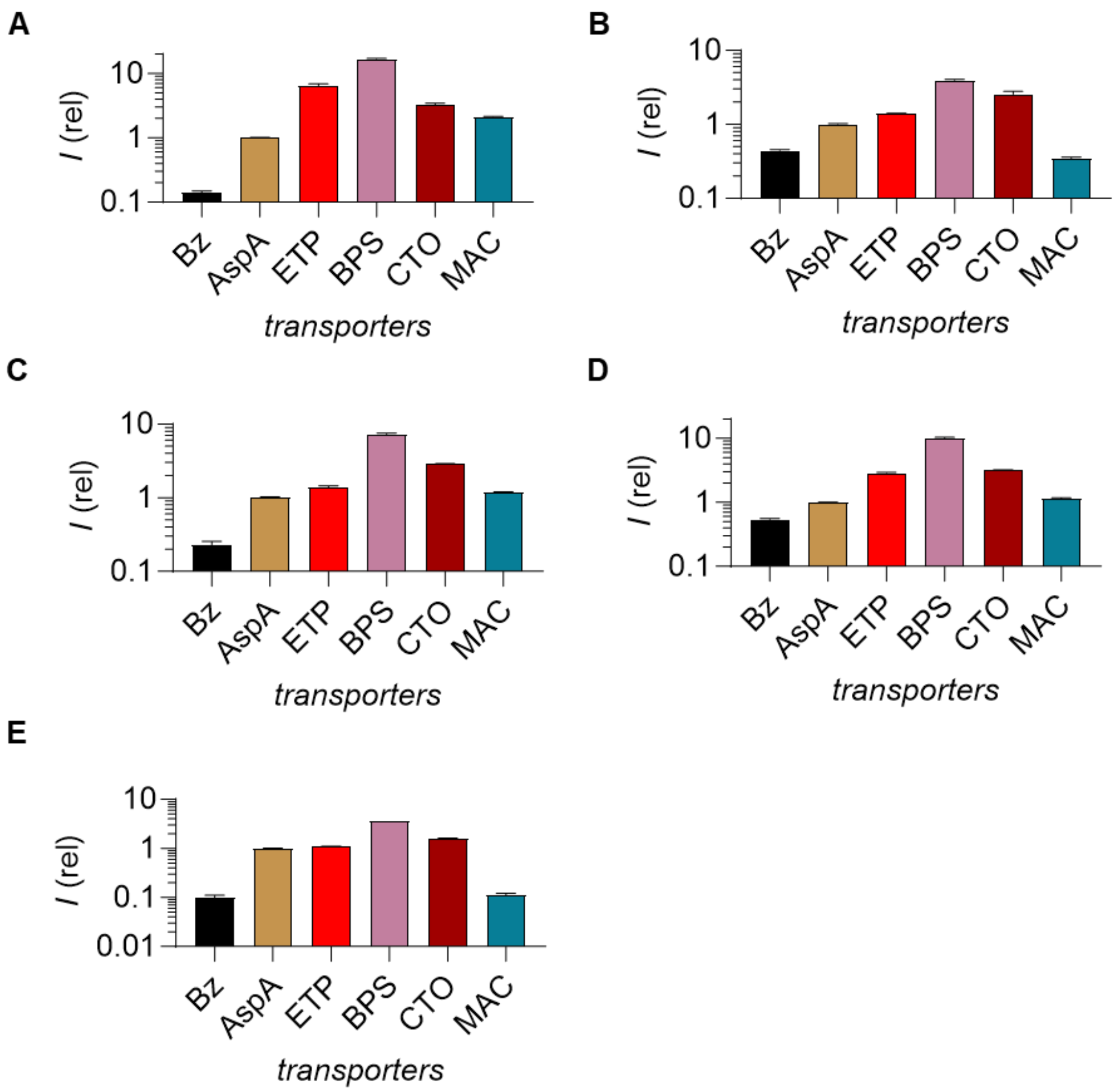

Figure S13. Relative fluorescence intensity *I* (rel) ± SEM of CAX transporters in A) HK, B) MDA-MB-231, C) A-431, D) RPE-1 and E) MCF-7 cells.

6. AHCHT Inhibitor Screening

6.1. General Experimental Procedure

Procedures were modified from references S1 and S8: **Pre-incubation method.** The cells were prepared in a 96-well plate as described in section 4, then medium was removed, and cells were washed with PBS (3 × 3 mL/well) followed by fresh FDMEM (4 × 100 µL/well) using a plate washer (Biotek EL406®), and kept in 100 µL/well of the latter medium. Stock solutions of the inhibitors (3× final concentration), transporters (3× in FDMEM), a mixture of Hoechst 33342 (50 µg/mL) and PI (5 µg/mL) in PBS were prepared freshly in a 96-well V-bottom plate before adding to the cells. The inhibitor solutions from the V-bottom plate were added using an electronic multichannel pipette to the cells (50 µL/well) keeping a final volume of 150 µL/well and cells were incubated for 1 h under 5% CO_2 humidified atmosphere at 37 °C. Then, cells were washed with PBS (3 × 3 mL/well) and FDMEM (4 × 100 µL/well) using the plate washer (as described above), and the corresponding transporter from the V-bottom plate was added (50 µL/well) to the cells, giving a final volume of 150 µL/well and a final transporter concentration of 10 µM. The cells were incubated for an additional 30 minutes under 5% CO_2 humidified atmosphere at 37 °C. After that, the cells were washed with PBS (3 × 3 mL/well) and FDMEM (4 × 100 µL/well) using the plate washer. A solution of Hoechst 33342 and PI from the V-bottom plate was added (50 µL/well) to the cells to a final volume of 150 µL/well. After 10 min of incubation under 5% CO_2 humidified atmosphere at 37 °C, the cells were washed with PBS (9 × 3 mL/well) and kept in FDMEM for imaging. During live cell imaging, samples were kept at under 5% CO_2 atmosphere at 37 °C. The distribution of fluorescent signals was captured on a IXM-C automated wide-field fluorescence microscope acquiring 4 images per well using a 10× objective lens with 3 channels, blue for Hoechst 33342 (377/50 nm excitation filter; 477/60 nm emission filter), green for FITC transporter (475/34 nm excitation filter; 536/40 nm emission filter) and red for PI (531/40 nm excitation filter; 593/40 nm emission filter). **Co-Incubation Method.** The overall process is the same as in pre-incubation method, except for the washing step after incubating

with inhibitors, the transporter was added to the cells without washing (resulting in 165 μL/well). Duplicates were performed for each condition.

6.2. Data Analysis

Fluorescence intensities per cell were extracted for each of conditions from the microscope images by the procedure described in section 5.2. The obtained values were normalized against those obtained with only the transporter ($I_{rel} = 1$) and without transporter ($I_{rel} = 0$). The resulting dependence of the relative fluorescent intensity values (I_{rel}) to the concentration of inhibitors ($c_{inhibitor}$) was plotted and fitted with Equation (S1) to retrieve the half maximal inhibitory concentration (IC_{50}) and the Hill coefficient (n). MIC values were estimated from the fit curve as the concentration at which 15% of inhibition was observed.

$$I_{rel} = 1 / (1 + (IC_{50} / c_{inhibitor})^{-n}) \qquad (S1)$$

Relative cell viability (RV) for each condition in the presence of inhibitors was calculated as the count of Hoechst 33342 stained cells minus the count of PI stained cells divided by the count of Hoechst 33342 stained cells in absence of inhibitor. The resulting dependence of the relative cell viability (RV_{rel}) to the concentration of inhibitors ($c_{inhibitor}$) was plotted and fitted with Equation (S2) to retrieve the concentration causing 50% cell growth inhibition (RV_{50}) value and the Hill coefficient (n).

$$RV_{rel} = 1 / (1 + (RV_{50} / c_{inhibitor})^{-n}) \qquad (S2)$$

6.3. Inhibitor Screening in HK Cells

6.3.1. Fl-BPS Transporter

Co-Incubation

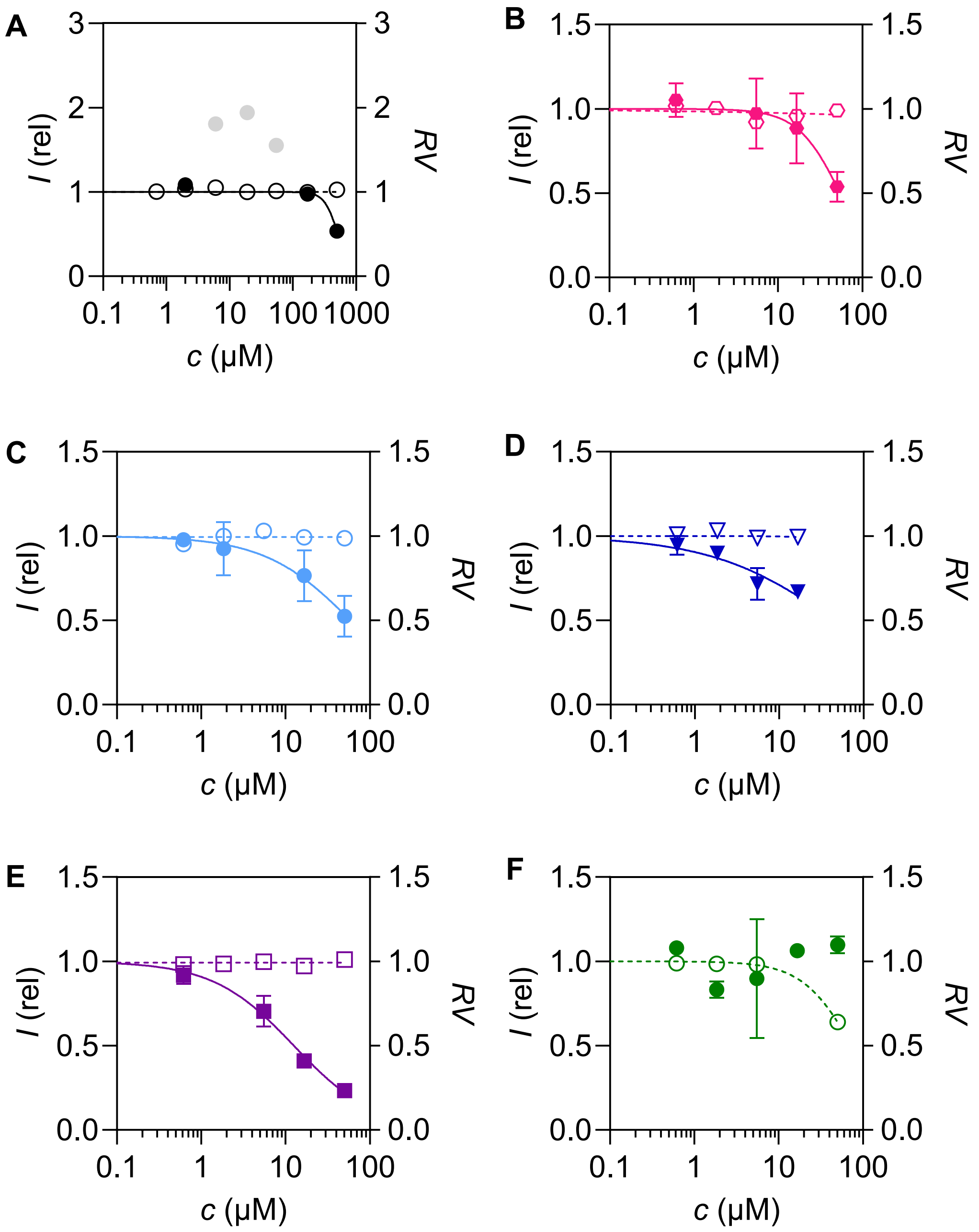

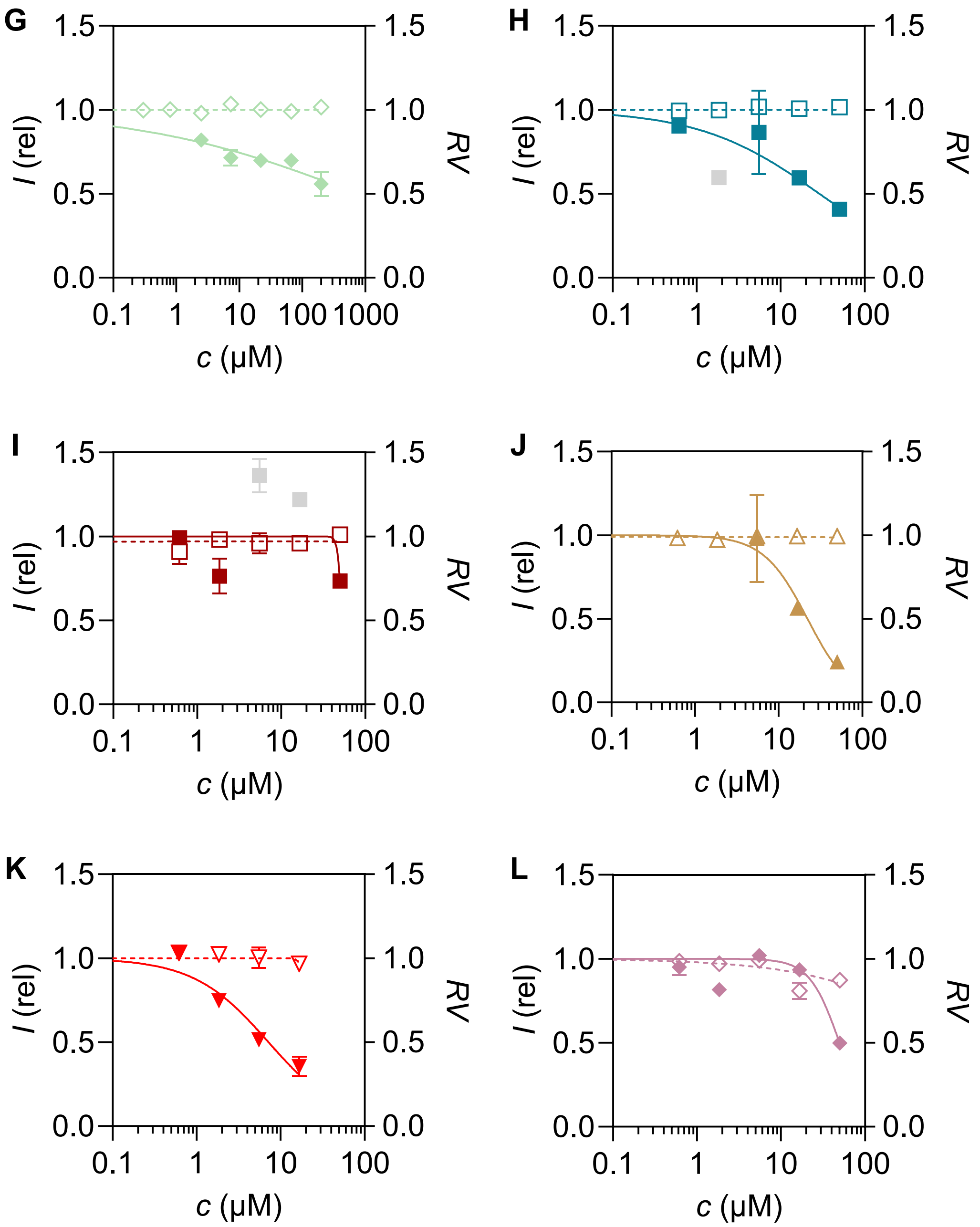

Figure S14. Relative fluorescence intensity *I* (rel) ± SEM (filled symbols) of **Fl-BPS** (10 µM) in HK cells and relative viability *RV* ± SEM (empty symbols) as a function of the concentration of A) **DTNB**, B) **EBX**, C) **EBS**, D) **BiC**, E) **AsC**, F) **SS**, G) **dMAC**, H) **MAC**, I) **CTO**, J) **AspA**, K) **ETP** and L) **BPS**. Grey symbols represent the data points excluded from the curve fit.

Table S2. Dependence of cellular uptake of **Fl-BPS** and cell viability in HK cells on the concentration of CAX inhibitors under co-incubation condition.[a]

Entry	Graph[b]	I[c]	MIC (μM)[d]	IC_{50} (μM)[e]	*n* (IC_{50})[f]	RV_{50} (μM)[g]	*n* (RV_{50})[h]
1	A	**DTNB**	350	>500	-	>500	-
2	B	**EBX**	20	(60 ± 20)	2 ± 1	>50	-
3	C	**EBS**	8	(60 ± 30)	0.9 ± 0.3	>50	-
4	D	**BiC**	2.4	(50 ± 20)	0.6 ± 0.3	>10	-
5	E	**AsC**	2	12 ± 2	0.9 ± 0.1	>50	-
6	F	**SS**	-	-	-	75 ± 5	1.5 ± 0.2
7	G	**dMAC**	0.6	>200	0.3 ± 0.1	>200	-
8	H	**MAC**	1.7	30 ± 3	0.6 ± 0.1	>50	-
9	I	**CTO**	50	(>50)	-	>50	-
10	J	**AspA**	8	20 ± 5	1.7 ± 0.6	>50	-
11	K	**ETP**	1.2	7 ± 1	1.0 ± 0.2	>20	
12	L	**BPS**	25	(>40)	3 ± 1	>30	

[a]Results from dose-response curves in Figure S14. [b]Corresponding graphs. [c]Inhibitor. [d]Concentration needed to reach 15% inhibition. [e]Concentration needed to reach 50% inhibition. [f]Hill coefficient for inhibition of cellular uptake. [g]Concentration needed to lower relative viability (RV) by 50%. [h]Hill coefficient for cell viability.

Pre-Incubation

The dose response curves under the pre-incubation condition for transporter **Fl-BPS** against inhibitors **CTO**, **ETP**, **BPS** were reported in reference S8.

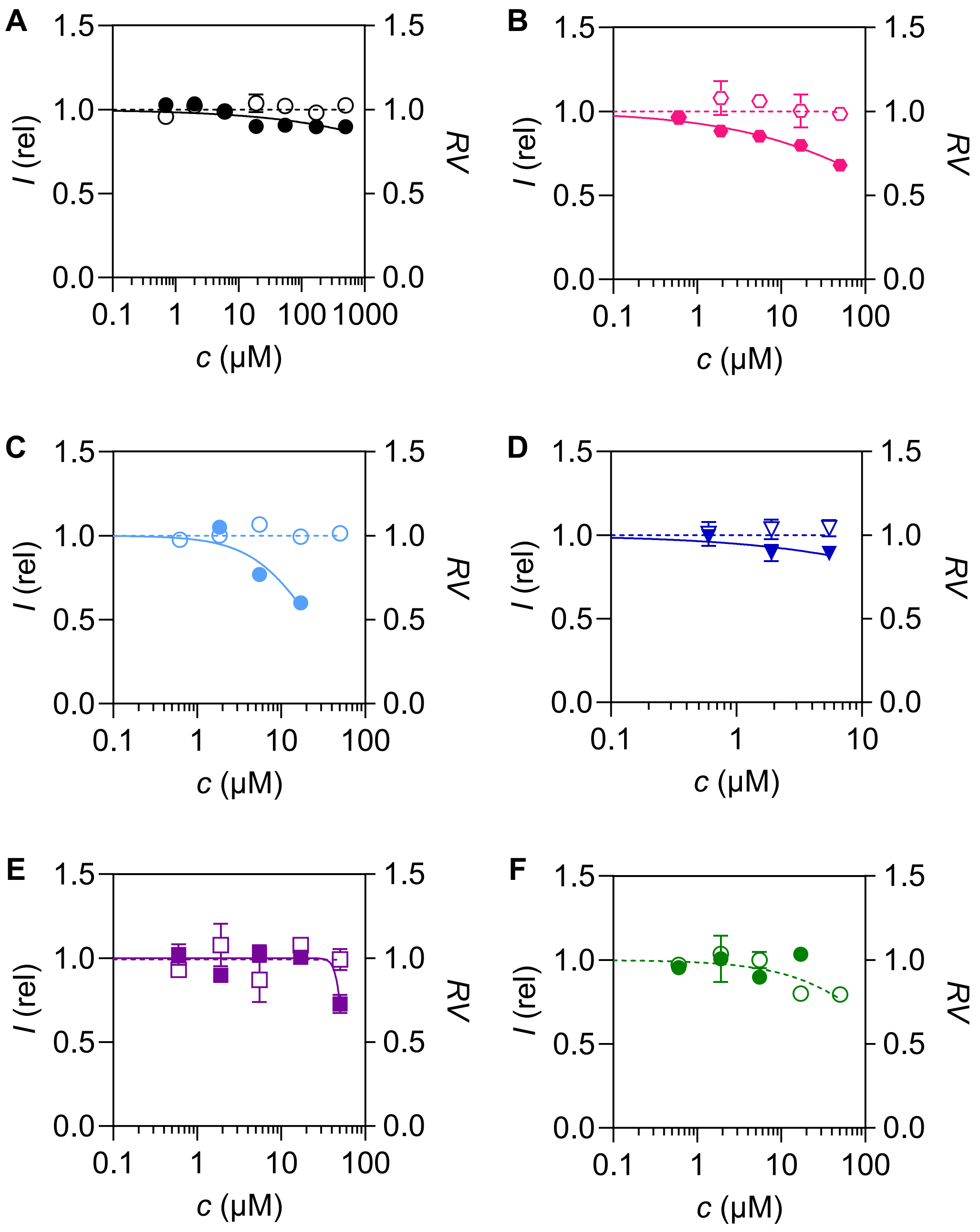

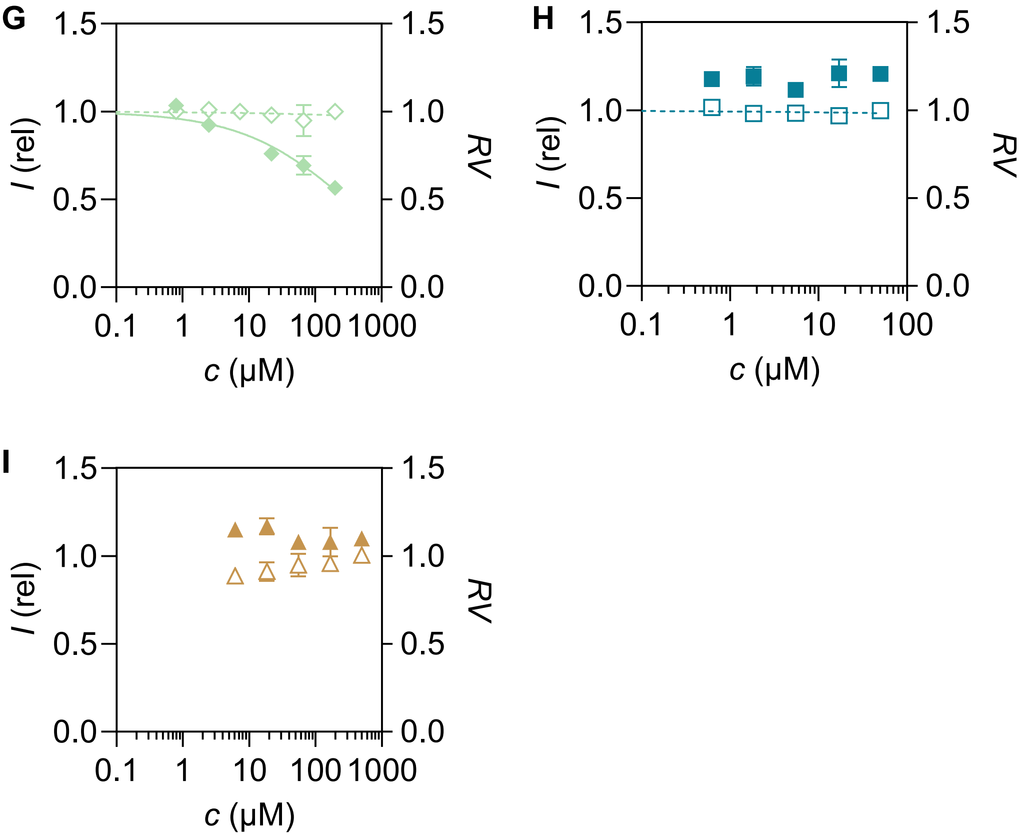

Figure S15. Relative fluorescence intensity *I* (rel) ± SEM (filled symbols) of **Fl-BPS** (10 µM) in HK cells and relative viability *RV* ± SEM (empty symbols) as a function of the concentration of A) **DTNB**, B) **EBX**, C) **EBS**, D) **BiC**, E) **AsC**, F) **SS**, G) **dMAC**, H) **MAC** and I) **AspA**.

Table S3. Dependence of cellular uptake of **Fl-BPS** and cell viability in HK cells on the concentration of CAX inhibitors under pre-incubation condition.[a]

Entry	Graph[b]	I[c]	MIC (µM)[d]	IC_{50} (µM)[e]	n (IC_{50})[f]	RV_{50} (µM)[g]	n (RV_{50})[h]
1	A	**DTNB**	-	-	-	>500	-
2	B	**EBX**	6	(300 ± 100)	0.5 ± 0.1	>50	-
3	C	**EBS**	6	20 ± 10	1.4 ± 0.5	>50	-
4	D	**BiC**	-	>5	-	>5	-
5	E	**AsC**	50	>50	-	>50	-
6	F	**SS**	-	-	-	>50	-
7	G	**dMAC**	15	(260 ± 80)	0.6 ± 0.1	>200	-
8	H	**MAC**	-	-	-	>50	-
9	I	**AspA**	-	-	-	>500	-

[a]Results from dose-response curves in Figure S15. [b]Results corresponding to the graphs above. [c]Inhibitor. [d]Concentration needed to reach 15% inhibition. [e]Concentration needed to reach 50% inhibition. [f]Hill coefficient for inhibition of cellular uptake. [g]Concentration needed to lower relative viability (RV) by 50%. [h]Hill coefficient for cell viability.

6.3.2. Fl-ETP Transporter

Co-Incubation

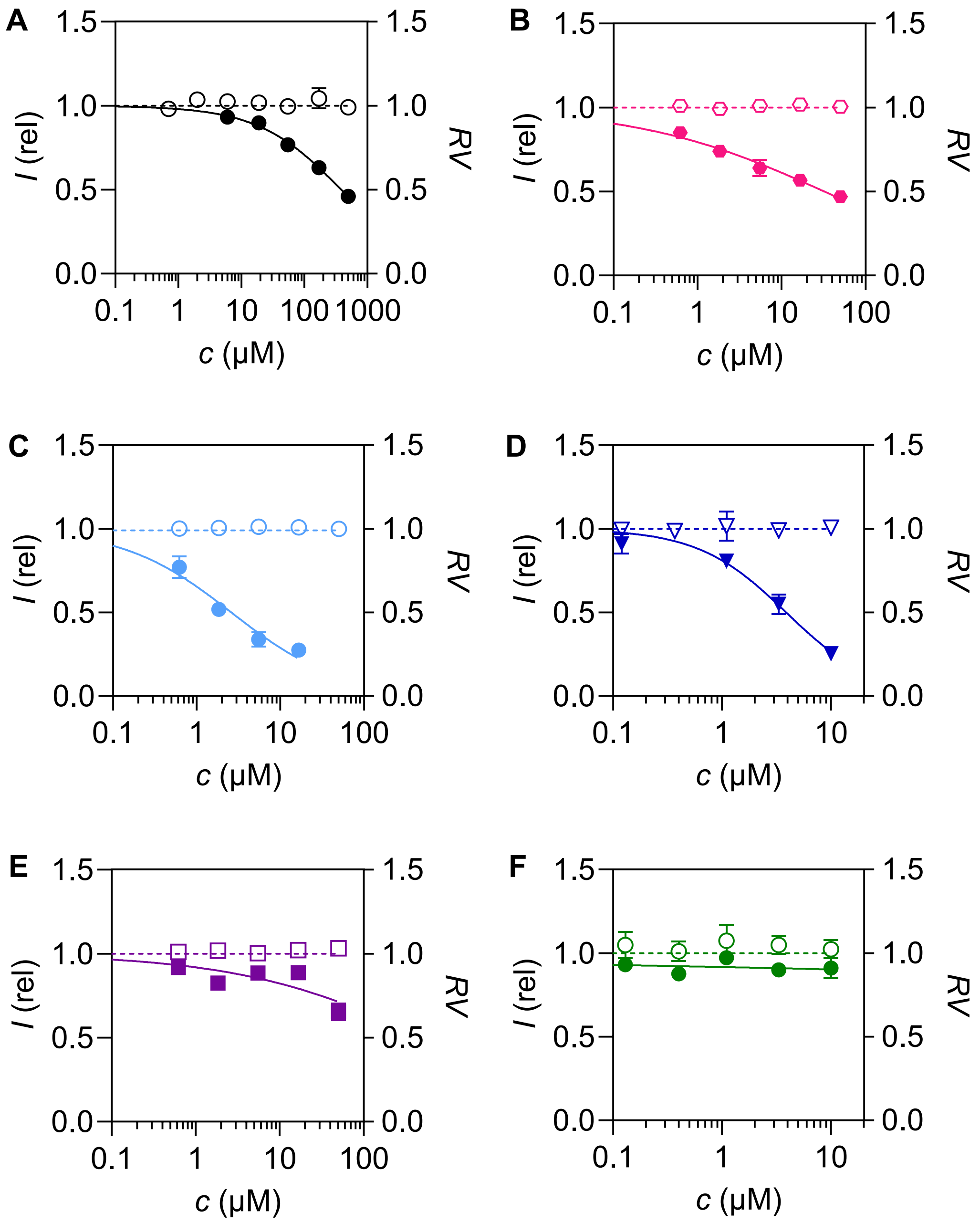

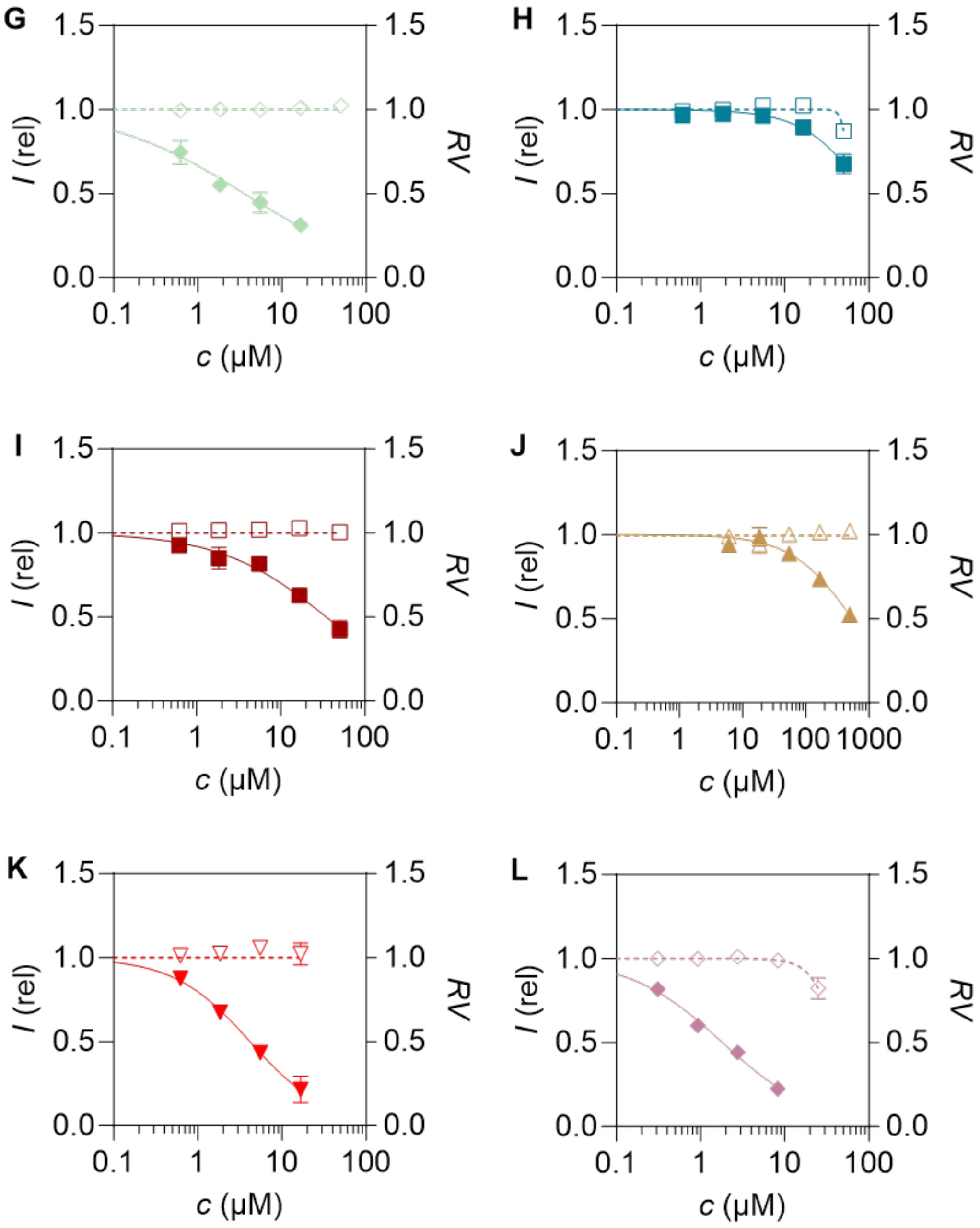

Figure S16. Relative fluorescence intensity *I* (rel) ± SEM (filled symbols) of **Fl-ETP** (10 µM) in HK cells and relative viability *RV* ± SEM (empty symbols) as a function of the concentration of A) **DTNB**, B) **EBX**, C) **EBS**, D) **BiC**, E) **AsC**, F) **SS**, G) **dMAC**, H) **MAC**, I) **CTO**, J) **AspA**, K) **ETP** and L) **BPS**.

Table S4. Dependence of cellular uptake of **Fl-ETP** and cell viability in HK cells on the concentration of CAX inhibitors under co-incubation condition.[a]

Entry	Graph[b]	I[c]	MIC (μM)[d]	IC_{50} (μM)[e]	*n* (IC_{50})[f]	RV_{50} (μM)[g]	*n* (RV_{50})[h]
1	A	**DTNB**	30	390 ± 30	2.6 ± 0.1	>500	-
2	B	**EBX**	<0.6	35 ± 5	0.4 ± 0.1	>50	-
3	C	**EBS**	<0.6	2.6 ± 0.3	0.7 ± 0.1	>50	-
4	D	**BiC**	0.7	3.2 ± 0.4	0.7 ± 0.1	>10	-
5	E	**AsC**	4	>50	-	>50	-
6	F	**SS**	-	-	-	>10	-
7	G	**dMAC**	<0.6	3.6 ± 0.5	1.5 ± 0.1	>50	-
8	H	**MAC**	20	(95 ± 15)	1.2 ± 0.2	>50	-
9	I	**CTO**	3	35 ± 5	0.4 ± 0.1	>50	-
10	J	**AspA**	80	(550 ± 70)	0.9 ± 0.1	>500	-
11	K	**ETP**	0.6	4.2 ± 0.3	1.0 ± 0.1	>40	
12	L	**BPS**	<0.3	1.8 ± 0.1	0.8 ± 0.1	(44)	3 ± 1

[a]Results from dose-response curves in Figure S16. [b]Results corresponding to the graphs above. [c]Inhibitor. [d]Concentration needed to reach 15% inhibition. [e]Concentration needed to reach 50% inhibition. [f]Hill coefficient for inhibition of cellular uptake. [g]Concentration needed to lower relative viability (RV) by 50%. [h]Hill coefficient for cell viability.

Pre-Incubation

The inhibition curves using the pre-incubation condition for transporter **Fl-ETP** against inhibitors **EBX**, **EBS**, **BiC**, **SS**, **ETP**, **BPS** were reported in reference S8.

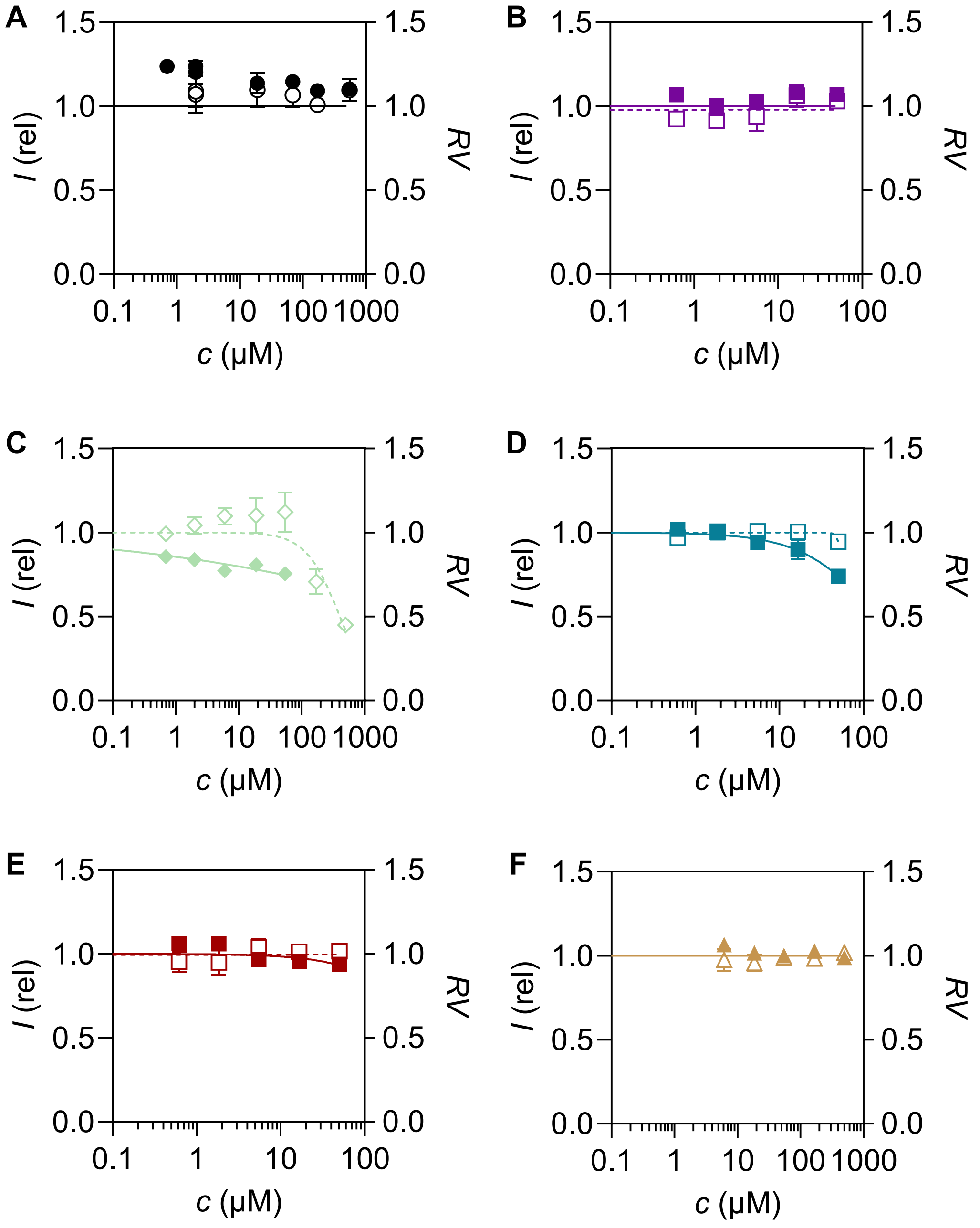

Figure S17. Relative fluorescence intensity *I* (rel) ± SEM (filled symbols) of **Fl-ETP** (10 μM) in HK cells and relative viability *RV* ± SEM (empty symbols) as a function of the concentration of A) **DTNB**, B) **AsC**, C) **dMAC**, D) **MAC**, E) **CTO** and F) **AspA**.

Table S5. Dependence of cellular uptake of **Fl-ETP** and cell viability in HK cells on the concentration of CAX inhibitors under pre-incubation condition.[a]

Entry	Graph[b]	I[c]	MIC (μM)[d]	IC_{50} (μM)[e]	*n* (IC_{50})[f]	RV_{50} (μM)[g]	*n* (RV_{50})[h]
1	A	**DTNB**	-	-	-	>500	-
2	B	**AsC**	-	-	-	>50	-
3	C	**dMAC**	1	>>200	-	400 ± 70	1.5 ± 0.5
4	D	**MAC**	25	(150 ± 100)	1.0 ± 0.5	>50	-
5	E	**CTO**	-	-	-	>50	-
6	F	**AspA**	-	-	-	>500	-

[a]Results from dose-response curves in Figure S17. [b]Results corresponding to the graphs above. [c]Inhibitor. [d]Concentration needed to reach 15% inhibition. [e]Concentration needed to reach 50% inhibition. [f]Hill coefficient for inhibition of cellular uptake. [g]Concentration needed to lower relative viability (RV) by 50%. [h]Hill coefficient for cell viability.

6.3.3. Fl-AspA Transporter

Co-Incubation

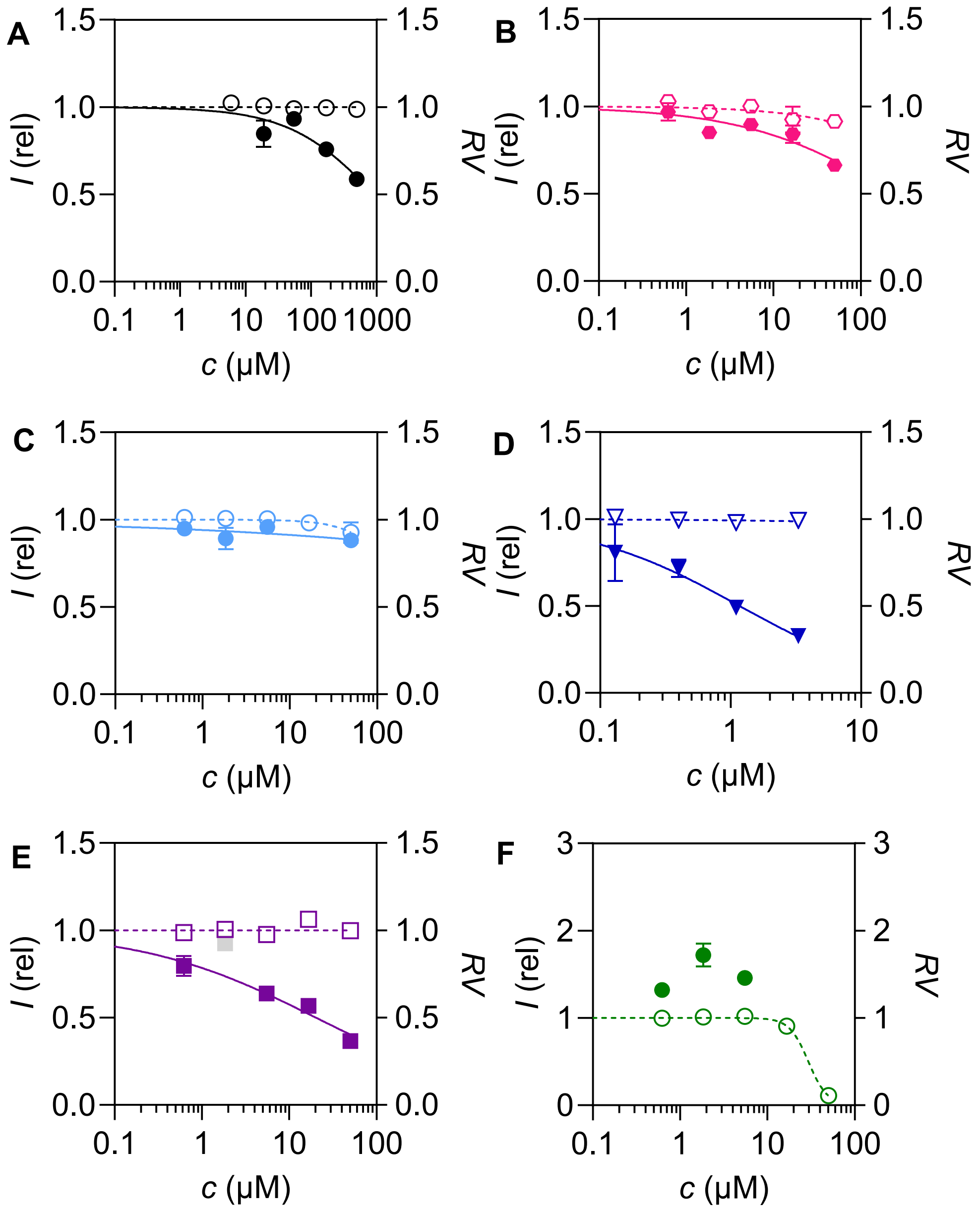

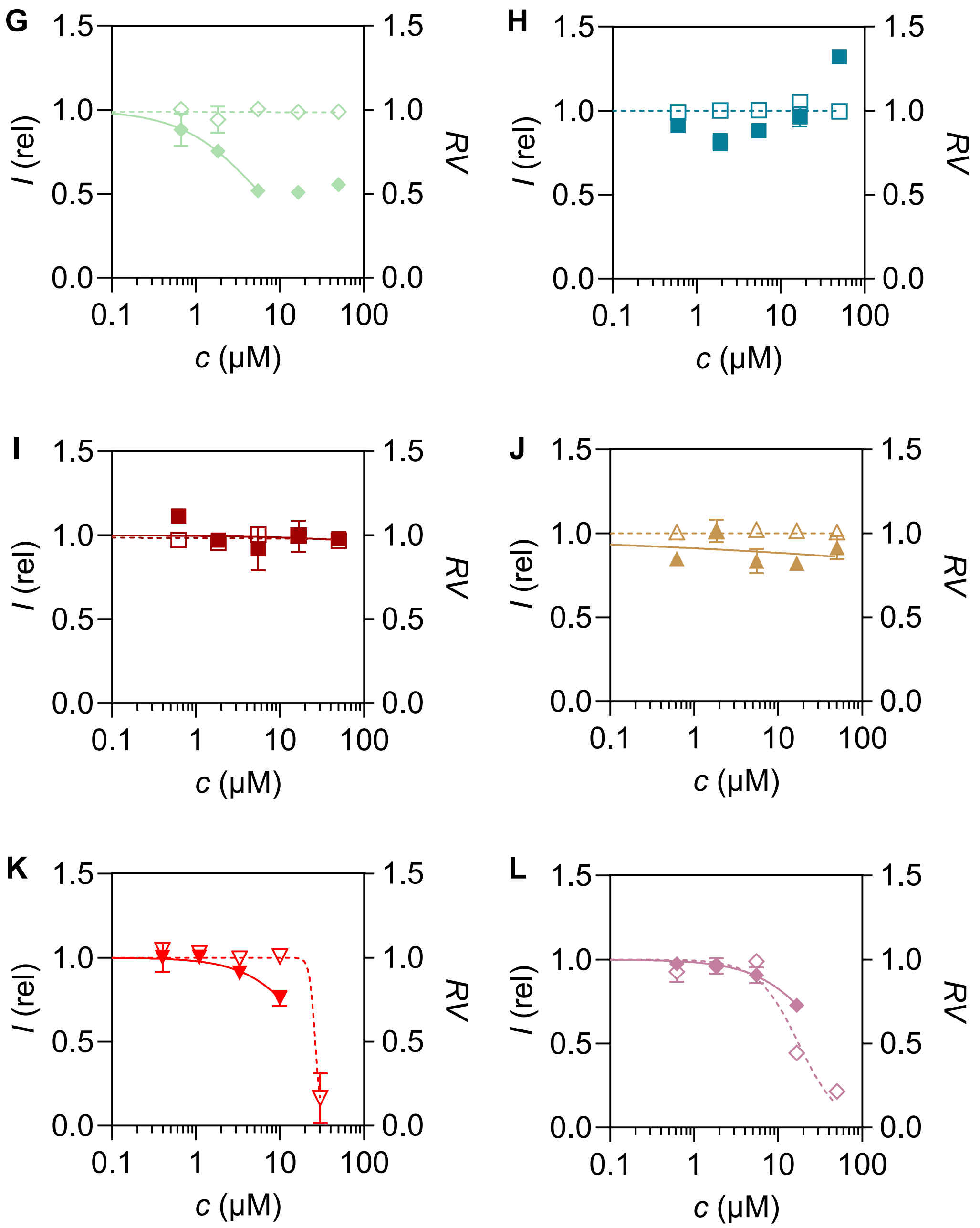

Figure S18. Relative fluorescence intensity *I* (rel) ± SEM (filled symbols) of **Fl-AspA** (10 µM) in HK cells and relative viability *RV* ± SEM (empty symbols) as a function of the concentration of A) **DTNB**, B) **EBX**, C) **EBS**, D) **BiC**, E) **AsC**, F) **SS**, G) **dMAC**, H) **MAC**, I) **CTO**, J) **AspA**, K) **ETP** and L) **BPS**. Grey symbols represent the data points excluded from the curve fit.

Table S6. Dependence of cellular uptake of **Fl-AspA** and cell viability in HK cells on the concentration of CAX inhibitors under co-incubation condition.[a]

Entry	Graph[b]	I[c]	MIC (μM)[d]	IC_{50} (μM)[e]	*n* (IC_{50})[f]	RV_{50} (μM)[g]	*n* (RV_{50})[h]
1	A	**DTNB**	70	(900 ± 300)	0.7 ± 0.1	>500	-
2	B	**EBX**	10	(250 ± 150)	0.5 ± 0.1	>50	-
3	C	**EBS**	-	-	-	>50	-
4	D	**BiC**	<0.1	1.2 ± 0.3	0.7 ± 0.1	>4	-
5	E	**AsC**	<0.6	20 ± 3	0.7 ± 0.1	>50	-
6	F	**SS**	-	-	-	30 ± 1	4 ± 0.2
7	G	**dMAC**	1	6 ± 1	0.9 ± 0.1	>50	-
8	H	**MAC**	-	-	-	>50	-
9	I	**CTO**	-	-	-	>50	-
10	J	**AspA**	-	-	-	>50	-
11	K	**ETP**	6	(25 ± 10)	1.2 ± 0.5	~20	
12	L	**BPS**	14	(40 ± 10)	1.0 ± 0.2	20 ± 2	1.8 ± 0.3

[a]Results from dose-response curves in Figure S18. [b]Results corresponding to the graphs above. [c]Inhibitor. [d]Concentration needed to reach 15% inhibition. [e]Concentration needed to reach 50% inhibition. [f]Hill coefficient for inhibition of cellular uptake. [g]Concentration needed to lower relative viability (RV) by 50%. [h]Hill coefficient for cell viability.

Pre-Incubation

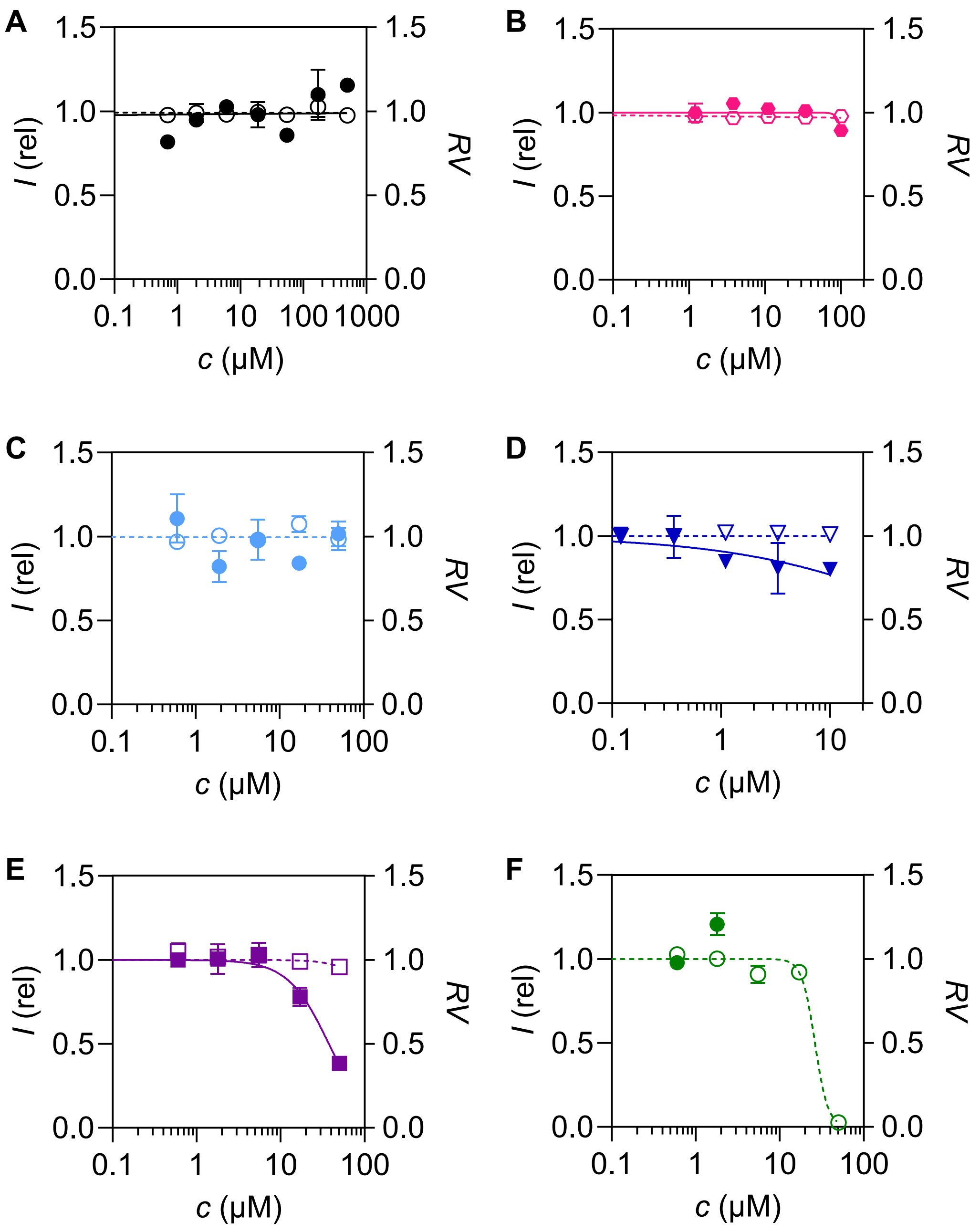

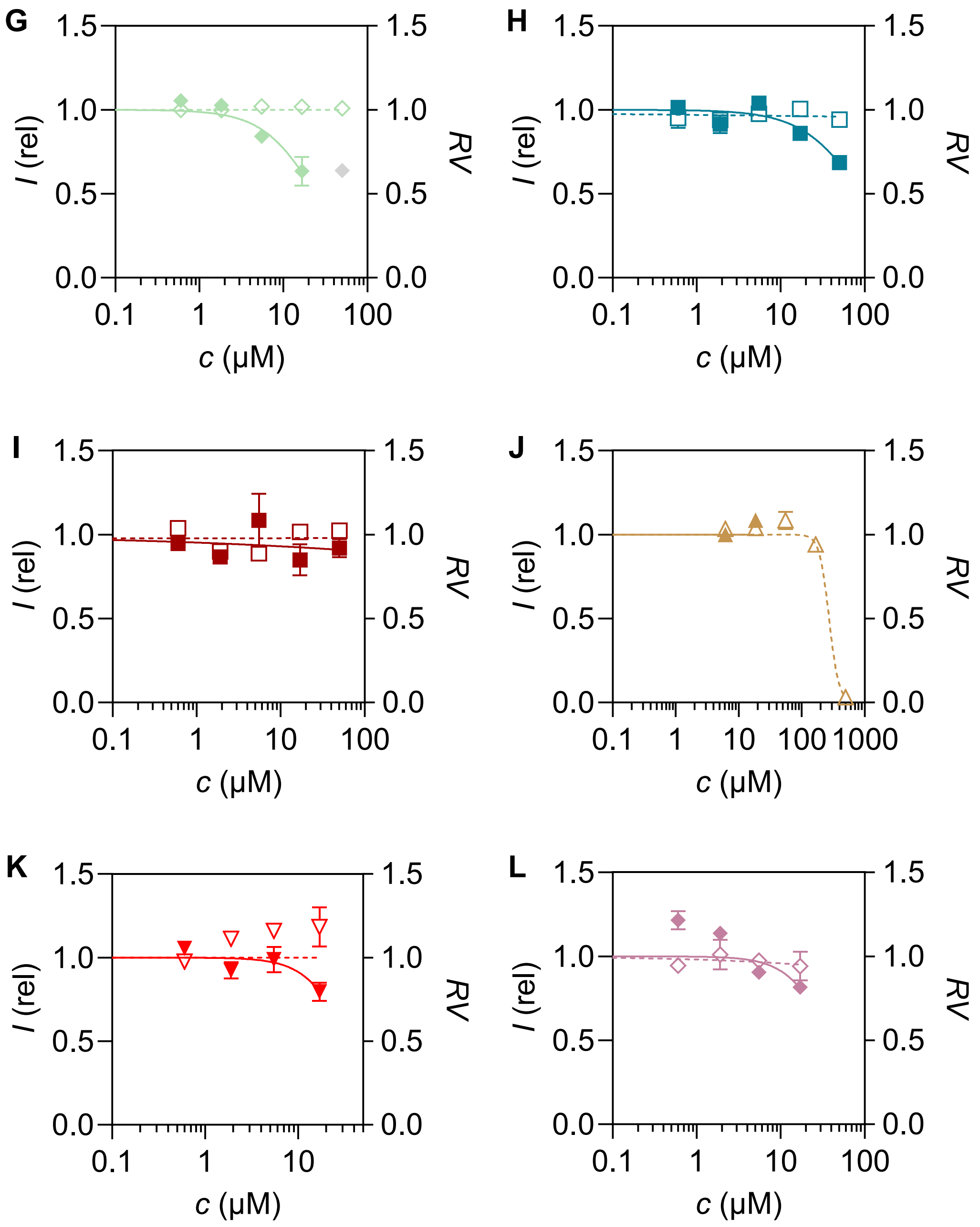

Figure S19. Relative fluorescence intensity *I* (rel) ± SEM (filled symbols) of **Fl-AspA** (10 µM) in HK cells and relative viability *RV* ± SEM (empty symbols) as a function of the concentration of A) **DTNB**, B) **EBX**, C) **EBS**, D) **BiC**, E) **AsC**, F) **SS**, G) **dMAC**, H) **MAC**, I) **CTO**, J) **AspA**, K) **ETP** and L) **BPS**. Grey symbols represent the data points excluded from the curve fit.

Table S7. Dependence of cellular uptake of **Fl-AspA** and cell viability in HK cells on the concentration of CAX inhibitors under pre-incubation condition.[a]

Entry	Graph[b]	I[c]	MIC (μM)[d]	IC_{50} (μM)[e]	n (IC_{50})[f]	RV_{50} (μM)[g]	n (RV_{50})[h]
1	A	**DTNB**	-	-	-	>500	-
2	B	**EBX**	-	-	-	>50	-
3	C	**EBS**	-	-	-	>50	-
4	D	**BiC**	3	(125 ± 15)	0.5 ± 0.1	>10	-
5	E	**AsC**	14	40 ± 3	1.8 ± 0.3	>50	-
6	F	**SS**	-	-	-	~25	-
7	G	**dMAC**	7	(25 ± 15)	1.4 ± 0.8	>50	-
8	H	**MAC**	20	(100 ± 25)	1.2 ± 0.3	>50	-
9	I	**CTO**	-	-	-	>50	-
10	J	**AspA**	-	-	-	~250	-
11	K	**ETP**	14	(30 ± 3)	2.0 ± 0.3	>25	-
12	L	**BPS**	14	(40 ± 10)	1.5 ± 0.5	>30	-

[a]Results from dose-response curves in Figure S19. [b]Results corresponding to the graphs above. [c]Inhibitor. [d]Concentration needed to reach 15% inhibition. [e]Concentration needed to reach 50% inhibition. [f]Hill coefficient for inhibition of cellular uptake. [g]Concentration needed to lower relative viability (RV) by 50%. [h]Hill coefficient for cell viability.

6.3.4. Fl-CTO Transporter

Co-Incubation

The inhibition curves using the co-incubation condition for transporter **Fl-CTO** against inhibitors **EBS**, **AsC**, **BiC**, **CTO**, **ETP**, **BPS** were reported in reference S6.

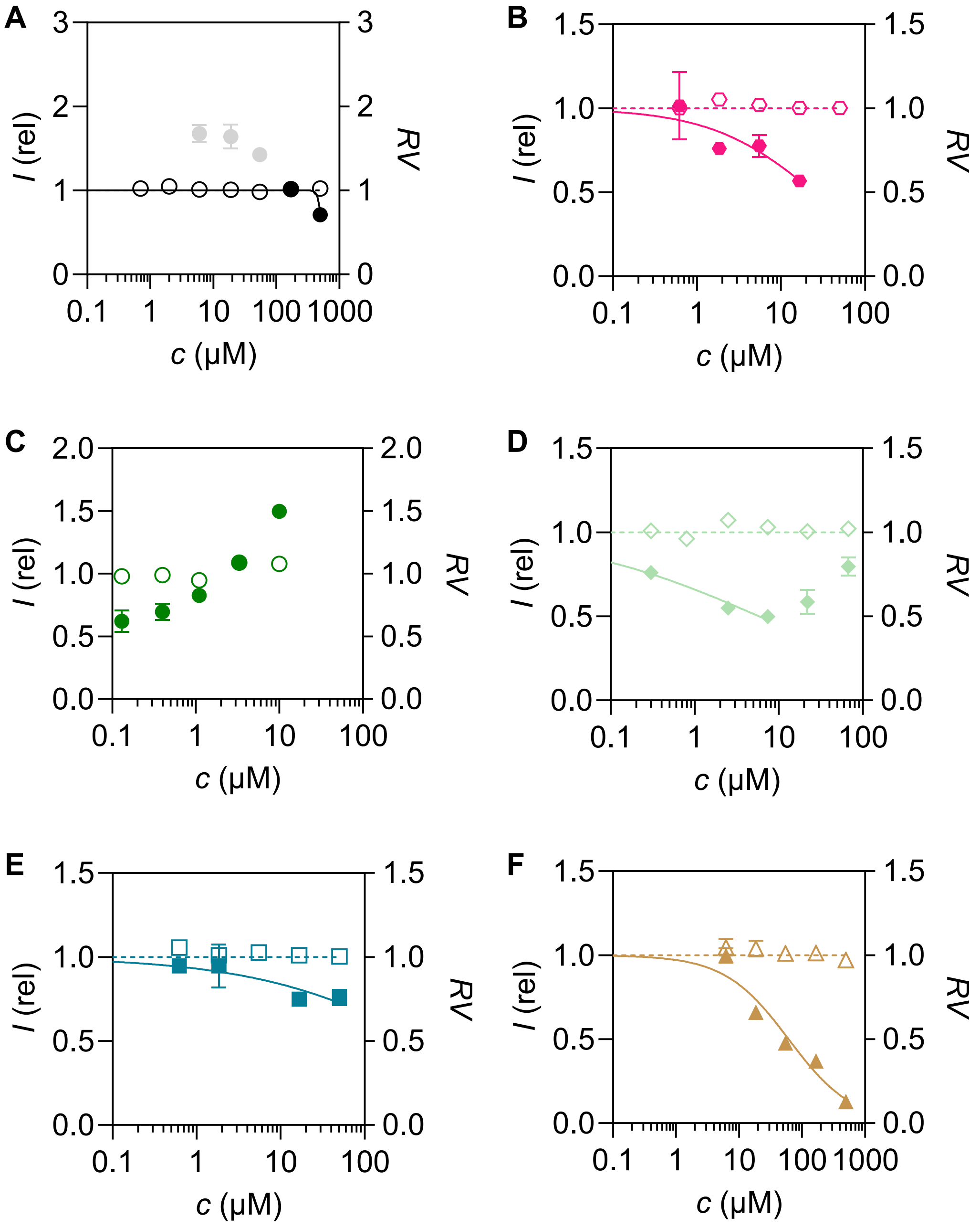

Figure S20. Relative fluorescence intensity *I* (rel) ± SEM (filled symbols) of **Fl-CTO** (10 µM) in HK cells and relative viability *RV* ± SEM (empty symbols) as a function of the concentration of A)

DTNB, B) **EBX**, C) **SS**, D) **dMAC**, E) **MAC** and F) **AspA**. Grey symbols represent the data points excluded from the curve fit.

Table S8. Dependence of cellular uptake of **Fl-CTO** and cell viability in HK cells on the concentration of CAX inhibitors under co-incubation condition.[a]

Entry	Graph[b]	I[c]	MIC (μM)[d]	IC_{50} (μM)[e]	n (IC_{50})[f]	RV_{50} (μM)[g]	n (RV_{50})[h]
1	A	**DTNB**	-	-	-	>500	-
2	B	**EBX**	2	25 ± 15	0.7 ± 0.3	>50	-
3	C	**SS**	<0.1	-	-	>10	-
4	D	**dMAC**	<0.3	6 ± 1	0.4 ± 0.1	>50	-
5	E	**MAC**	2	>>50	-	>50	-
6	F	**AspA**	8	60 ± 10	0.9 ± 0.1	>500	-

[a]Results from dose-response curves in Figure S20. [b]Results corresponding to the graphs above. [c]Inhibitor. [d]Concentration needed to reach 15% inhibition. [e]Concentration needed to reach 50% inhibition. [f]Hill coefficient for inhibition of cellular uptake. [g]Concentration needed to lower relative viability (RV) by 50%. [h]Hill coefficient for cell viability.

Pre-Incubation

The inhibition curves using the pre-incubation condition for transporter **Fl-CTO** against inhibitors **EBX**, **EBS**, **CTO**, were reported in reference S6.

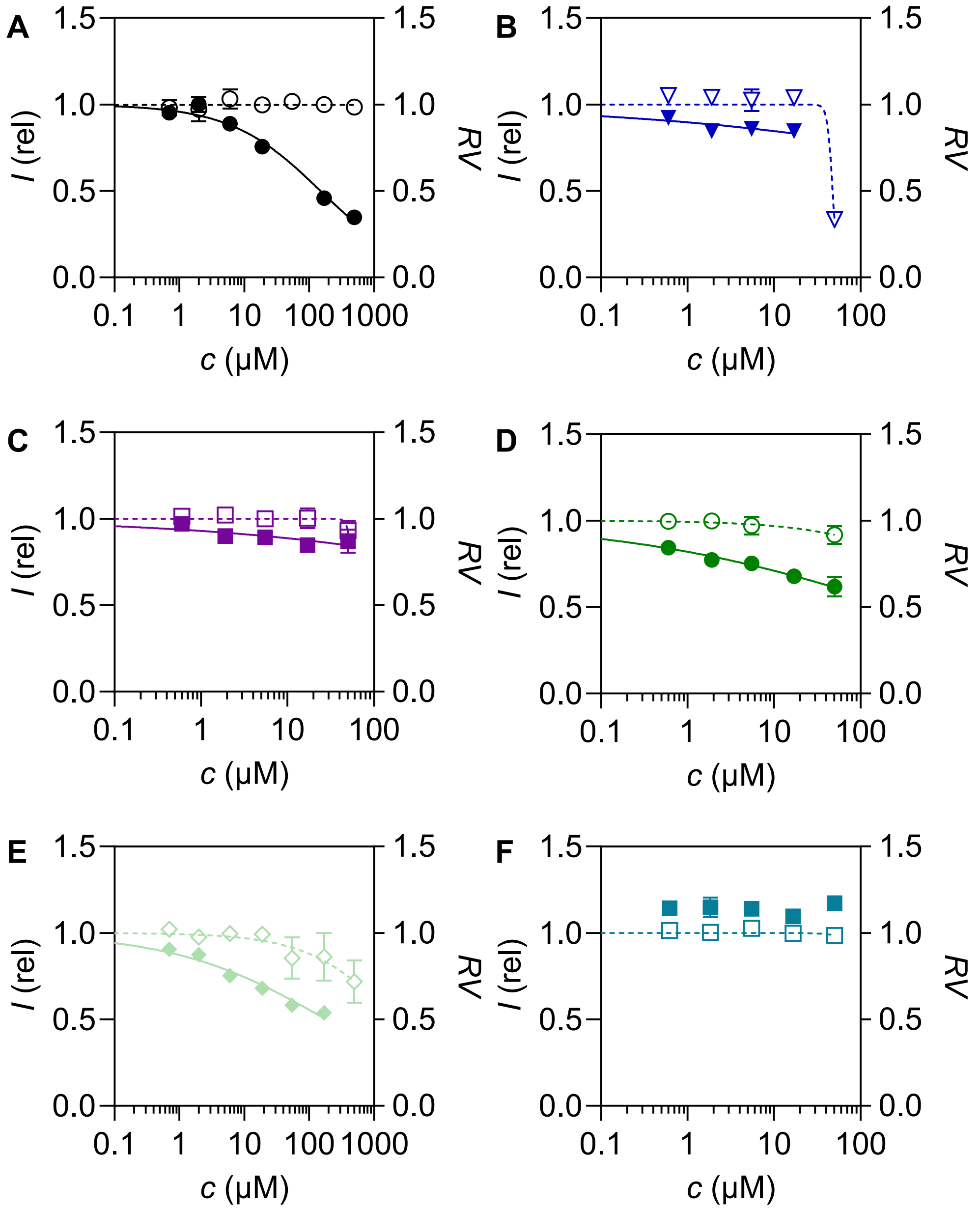

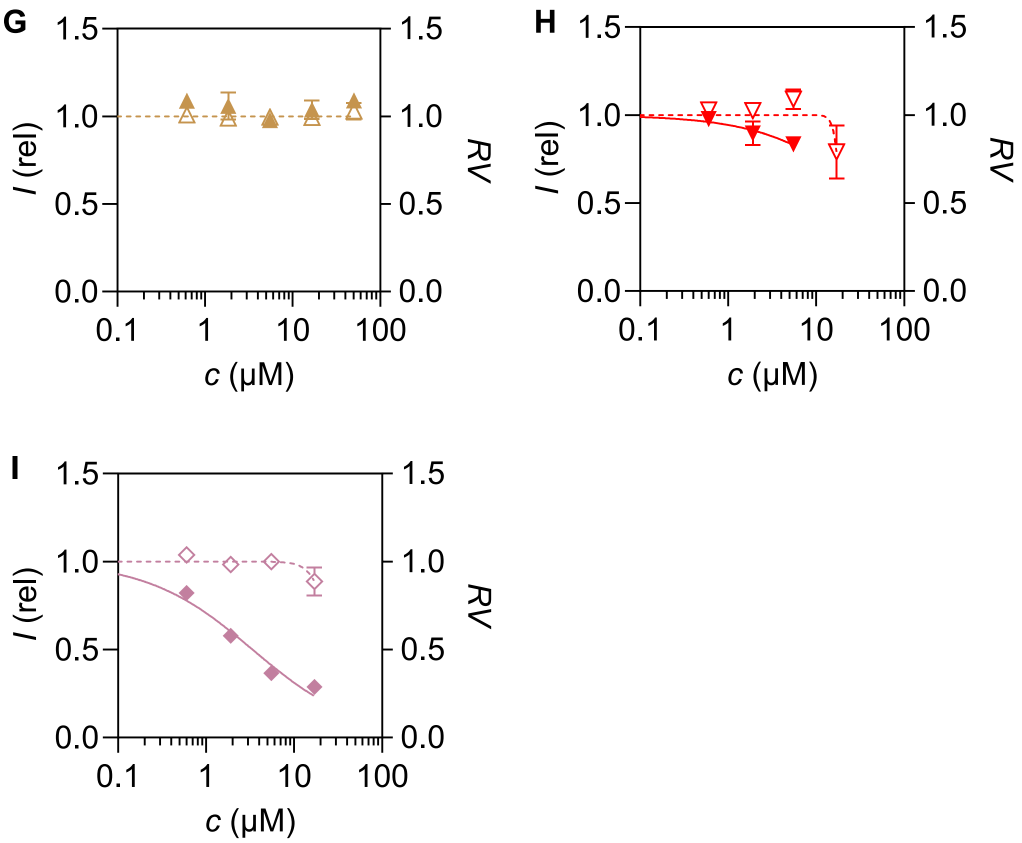

Figure S21. Relative fluorescence intensity *I* (rel) ± SEM (filled symbols) of **Fl-CTO** (10 µM) in HK cells and relative viability *RV* ± SEM (empty symbols) as a function of the concentration of A) **DTNB**, B) **BiC**, C) **AsC**, D) **SS**, E) **dMAC**, F) **MAC**, G) **AspA**, H) **ETP** and I) **BPS**. Grey symbols represent the data points excluded from the curve fit.

Table S9. Dependence of cellular uptake of **Fl-CTO** and cell viability in HK cells on the concentration of CAX inhibitors under pre-incubation condition.[a]

Entry	Graph[b]	I[c]	MIC (μM)[d]	IC_{50} (μM)[e]	n (IC_{50})[f]	RV_{50} (μM)[g]	n (RV_{50})[h]
1	A	**DTNB**	10	150 ± 15	0.6 ± 0.1	>500	-
2	B	**BiC**	-	-	-	~40	-
3	C	**AsC**	-	-	-	>50	-
4	D	**SS**	<0.6	(300 ± 100)	-	>50	-
5	E	**dMAC**	2	180 ± 40	0.4 ± 0.1	>200	-
6	F	**MAC**	-	-	-	>50	-
7	G	**AspA**	-	-	-	>50	-
8	H	**ETP**	4	(20 ± 3)	1.0 ± 0.1	~20	-
9	I	**BPS**	<0.6	3.4 ± 0.4	0.7 ± 0.1	>20	-

[a]Results from dose-response curves in Figure S21. [b]Results corresponding to the graphs above. [c]Inhibitor. [d]Concentration needed to reach 15% inhibition. [e]Concentration needed to reach 50% inhibition. [f]Hill coefficient for inhibition of cellular uptake. [g]Concentration needed to lower relative viability (RV) by 50%. [h]Hill coefficient for cell viability.

6.3.5. Fl-MAC Transporter

The inhibition curves using the co-incubation and pre-incubation conditions for transporter **Fl-MAC** against inhibitors **EBX**, **EBS**, **BiC**, **AsC**, **SS**, **dMAC**, **MAC**, **CTO**, **AspA**, **ETP** and **BPS** were reported in reference S1.

6.3.6. OPS-Cy5 Transporter

The inhibition curves using the pre-incubation condition for transporter **OPS-Cy5** against inhibitors **EBX**, **EBS**, **BiC**, **AsC**, **SS**, **dMAC**, **MAC**, **CTO**, **AspA**, **ETP** and **BPS** were reported in reference S11.

6.4. Inhibitor Screening in Various Cell Lines

The co-incubation conditions were used following the procedures described in sections 6.1 and 6.2. Duplicates were performed for each condition.

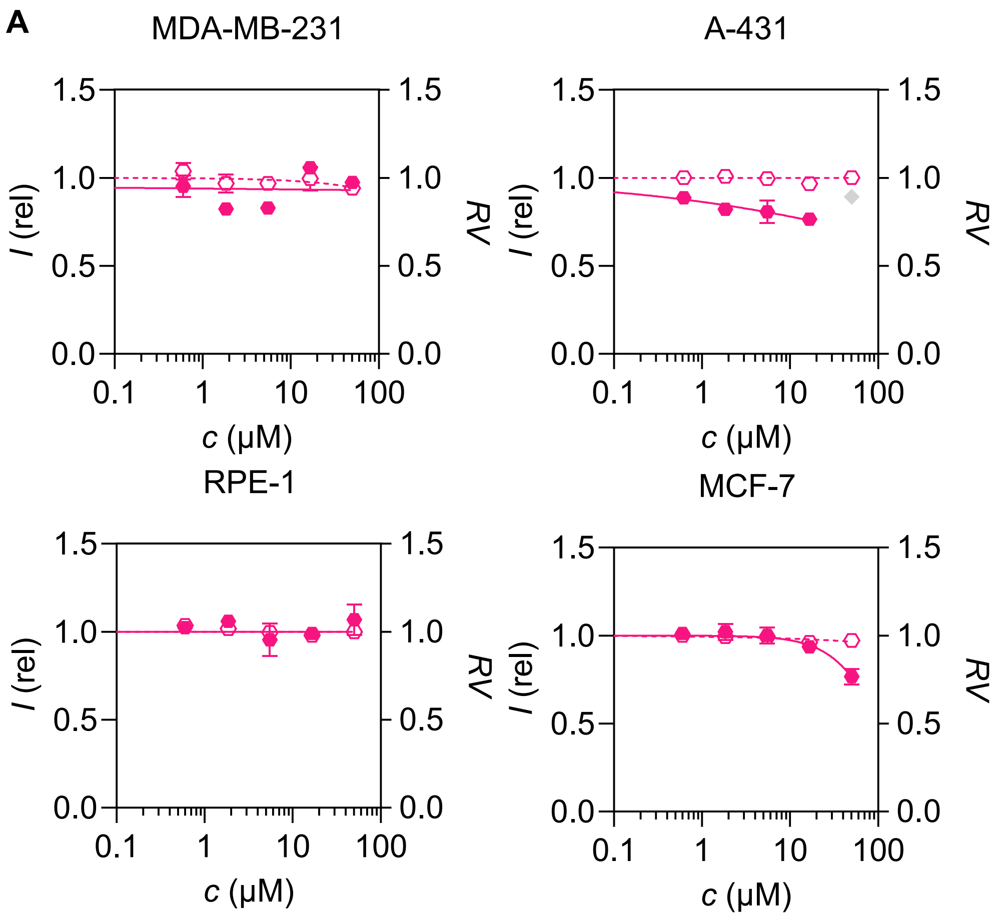

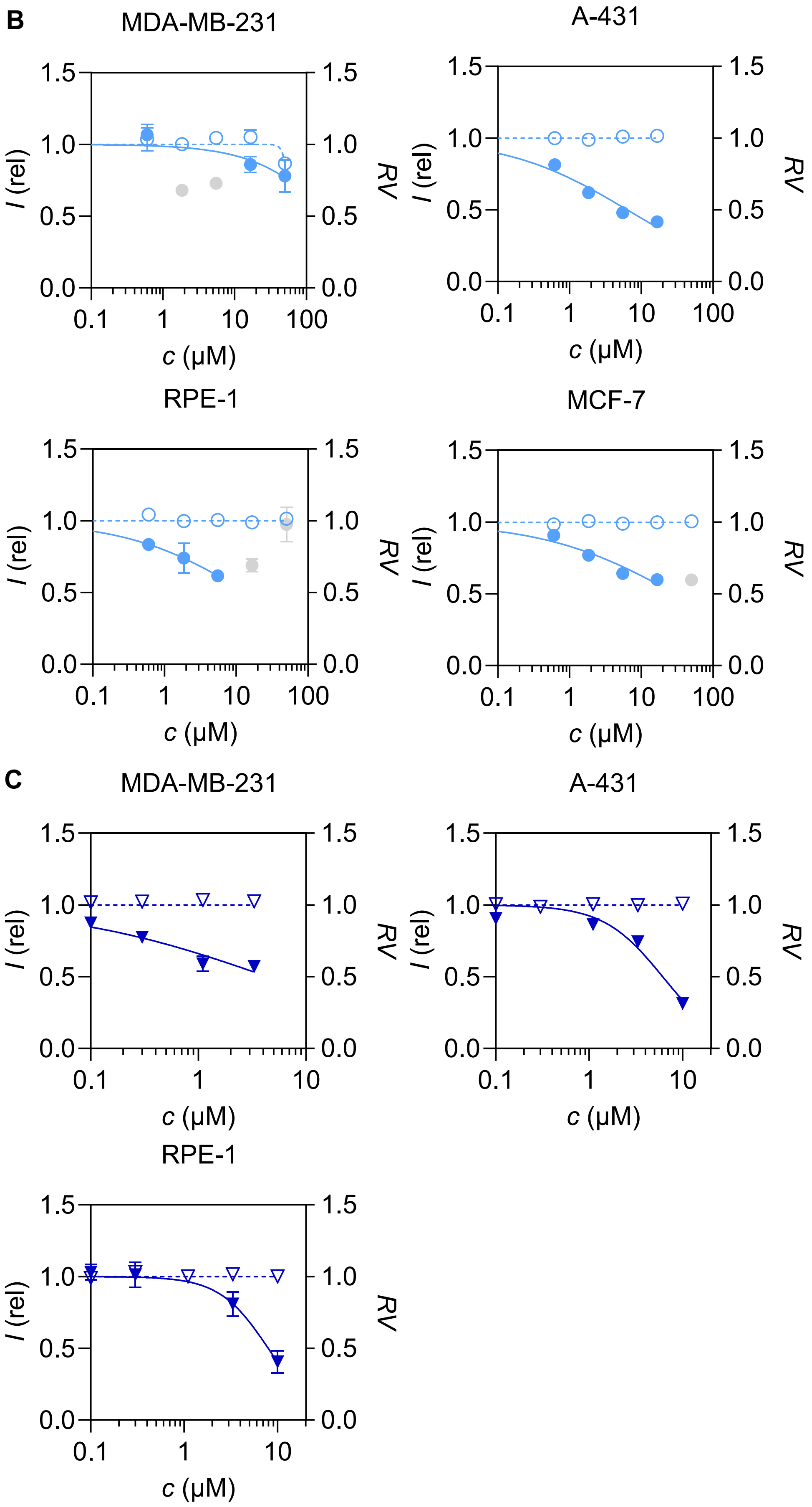
B
MDA-MB-231
A-431
RPE-1
MCF-7
C
MDA-MB-231
A-431
RPE-1
I (rel)
RV
c (μM)
1.5
1.0
0.5
0.0
0.1
1
10
100

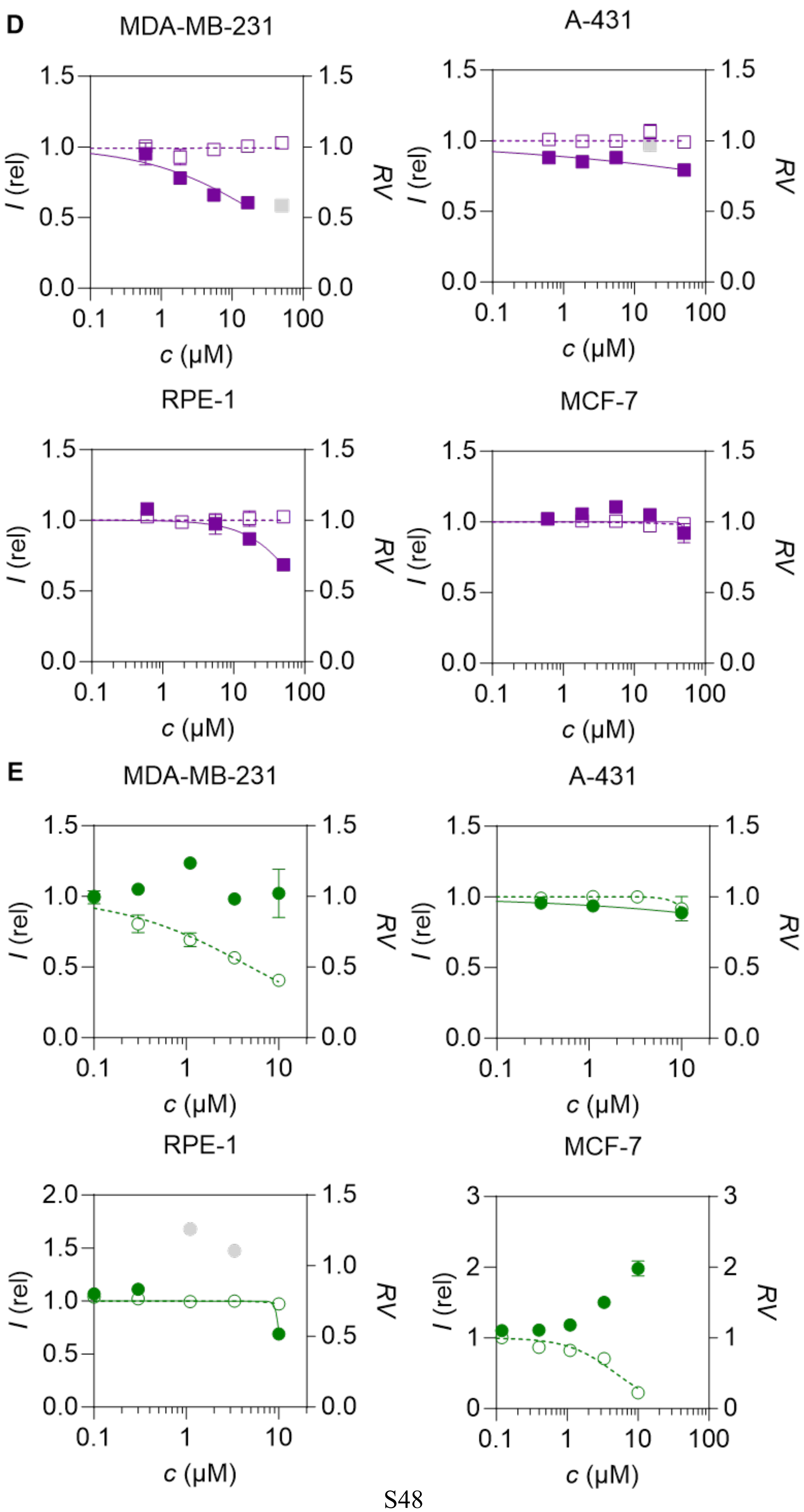

D
MDA-MB-231
A-431
RPE-1
MCF-7
E
MDA-MB-231
A-431
RPE-1
MCF-7
I (rel)
RV
c (μM)

F

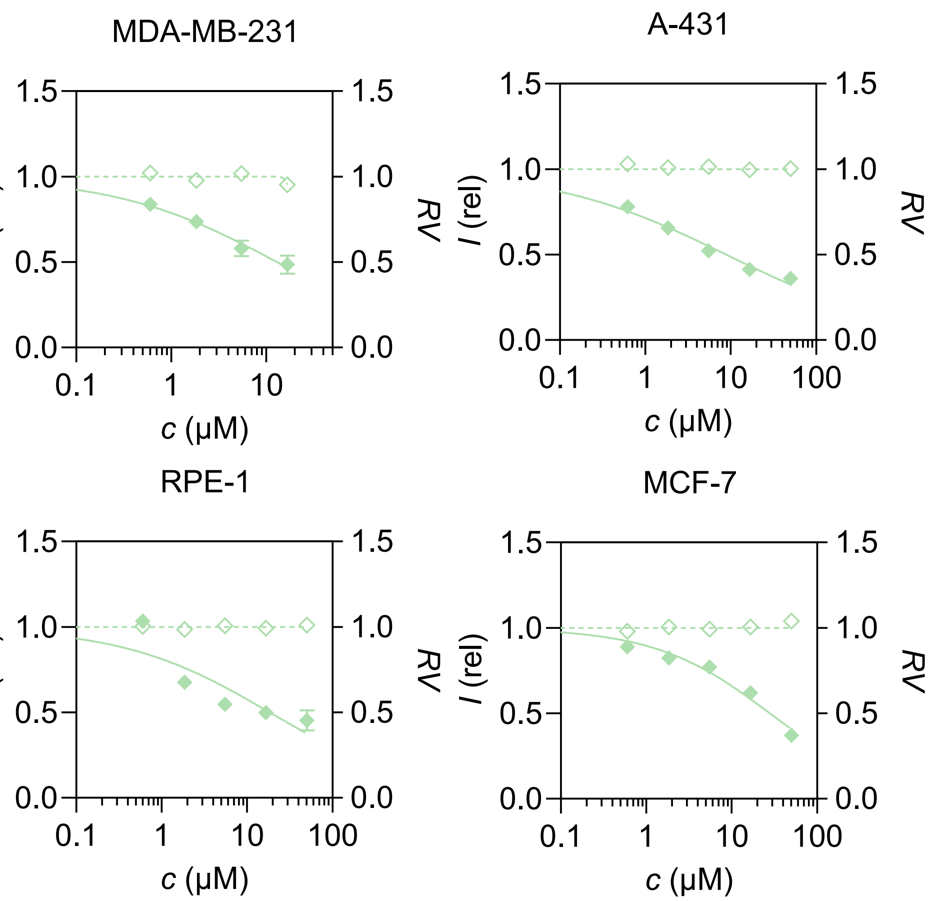

MDA-MB-231
A-431
RPE-1
MCF-7
I (rel)
RV
c (μM)
1.5
1.0
0.5
0.0
0.1
1
10
100

G

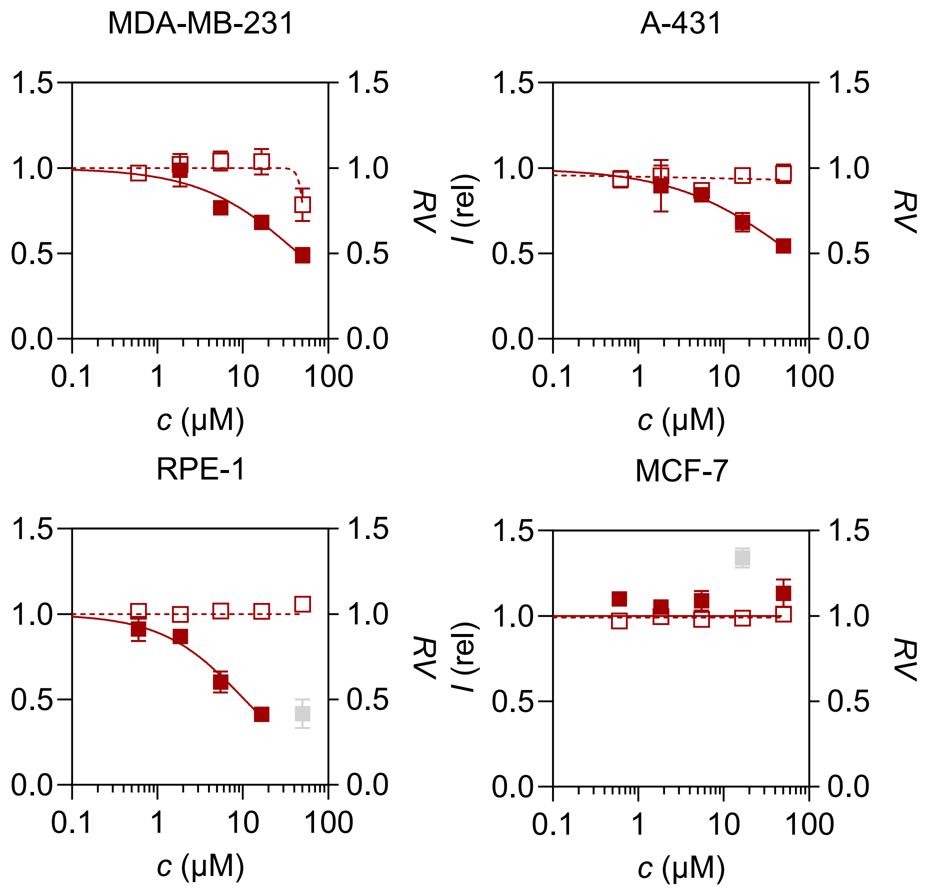

MDA-MB-231
A-431
RPE-1
MCF-7
I (rel)
RV
c (μM)
1.5
1.0
0.5
0.0
0.1
1
10
100

H

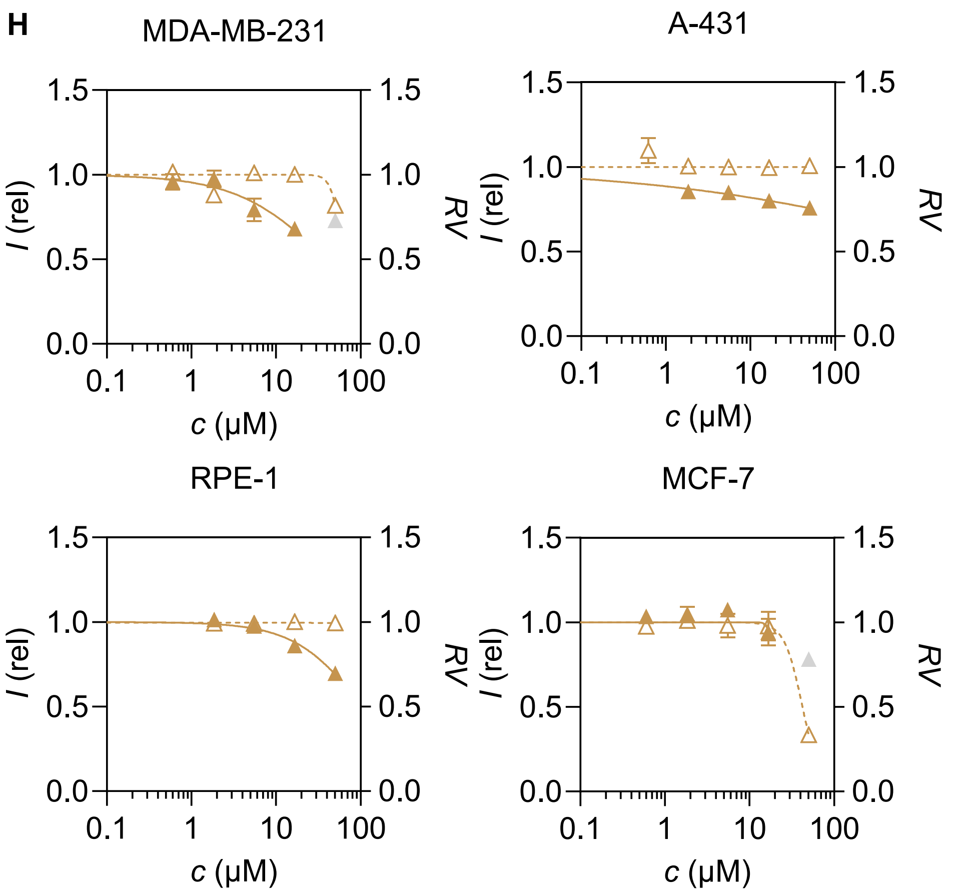
MDA-MB-231
A-431
RPE-1
MCF-7
I (rel)
RV
c (μM)
0.0
0.5
1.0
1.5
0.1
1
10
100

I

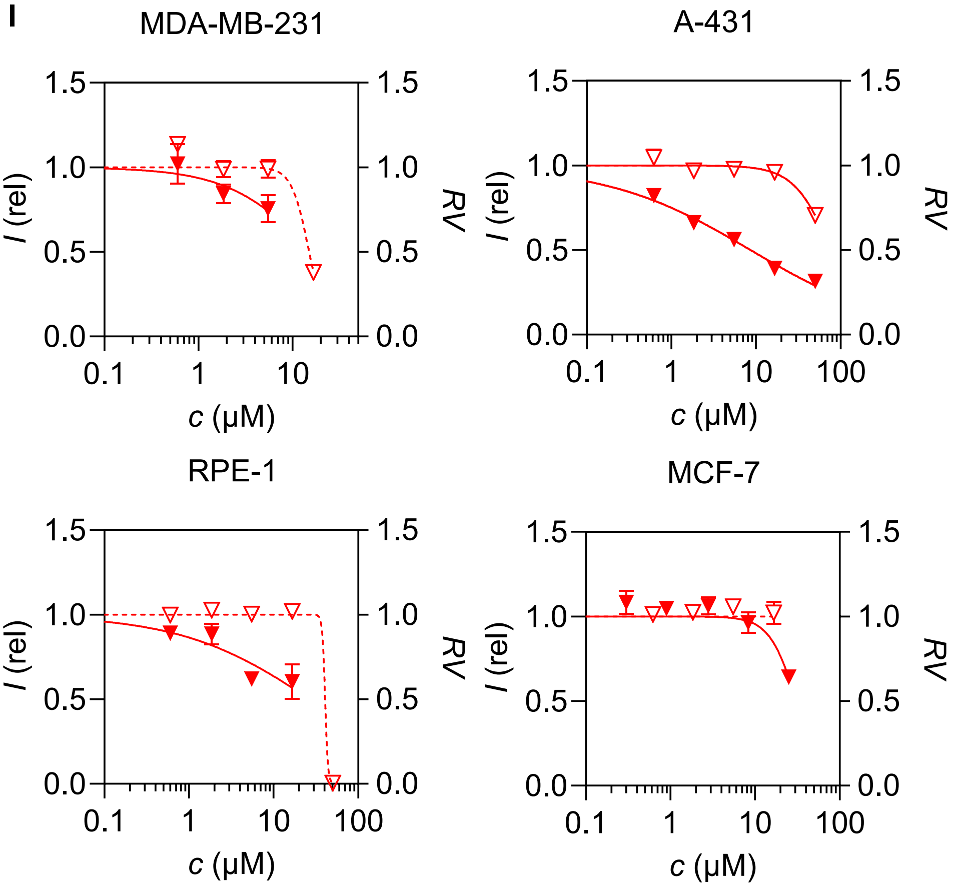
MDA-MB-231
A-431
RPE-1
MCF-7
I (rel)
RV
c (μM)
0.0
0.5
1.0
1.5
0.1
1
10
100

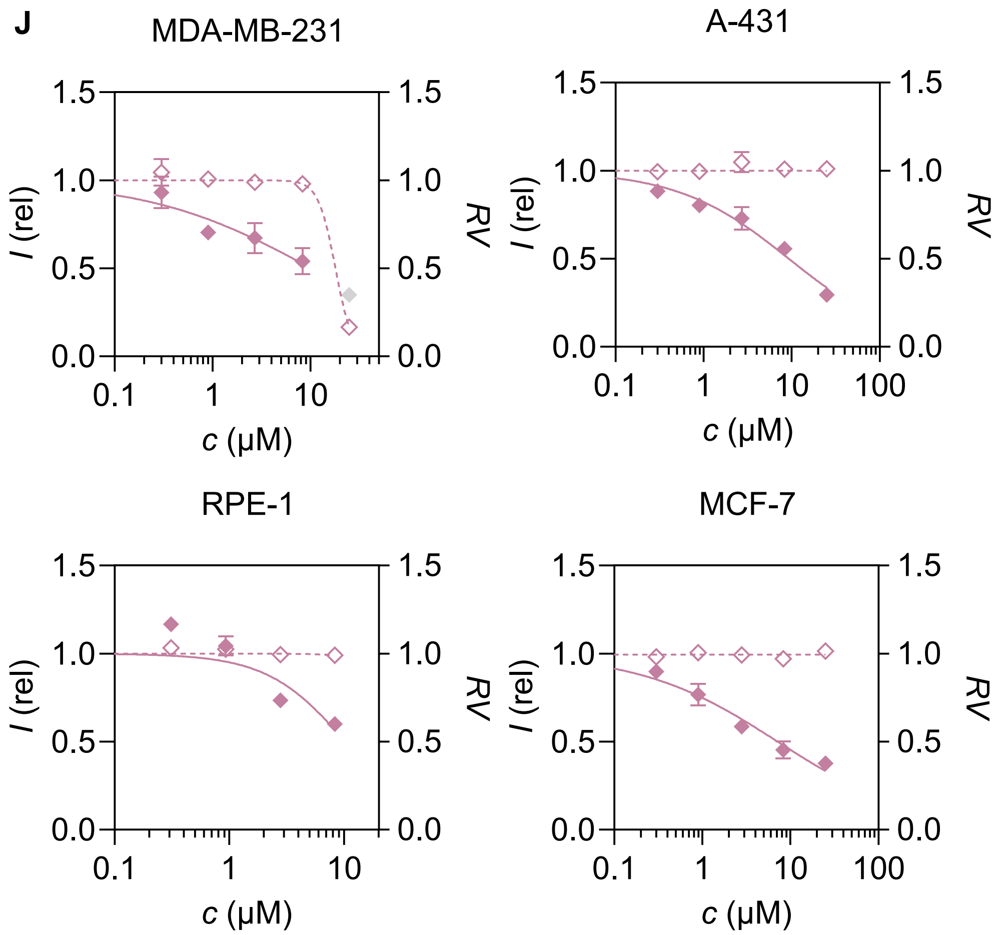

Figure S22. Relative fluorescence intensity *I* (rel) ± SEM (filled symbols) of **Fl-ETP** (10 µM) in different cells and relative viability *RV* ± SEM (empty symbols) as a function of the concentration of A) **EBX**, B) **EBS**, C) **BiC**, D) **AsC**, E) **SS**, F) **dMAC**, G) **CTO**, H) **AspA**, I) **ETP** and J) **BPS**. Grey symbols represent the data points excluded from the curve fit.

Table S10. Dependence of cellular uptake of **Fl-ETP** and cell viability in different cells on the concentration of CAX inhibitors under co-incubation condition.[a]

Entry	Graph[b]	I[c]	Cell[d]	MIC (μM)[e]	IC_{50} (μM)[f]	n (IC_{50})[g]	RV_{50} (μM)[h]	n (RV_{50})[i]
1	A	**EBX**	1	-	-	-	>50	-
2			2	1.5	>50	-	>50	-
3			3	-	>50	-	>50	-
4			4	35	(110 ± 30)	1.5 ± 0.5	>50	-
5	B	**EBS**	1	25	>50	-	>50	-
6			2	<0.6	6 ± 1	0.5 ± 0.1	>50	-
7			3	<0.6	(15 ± 5)	0.5 ± 0.1	>50	-
8			4	<0.6	(30 ± 10)	0.5 ± 0.1	>50	-
9	C	**BiC**	1	<0.1	5 ± 1	0.5 ± 0.1	>10	-
10			2	2	6.0 ± 0.1	1.4 ± 0.2	>10	-
11			3	3	8 ± 1	1.7 ± 0.4	>10	-
12	D	**AsC**	1	1	(30 ± 10)	0.5 ± 0.1	>50	-
13			2	-	-	-	>50	-
14			3	20	(95 ± 25)	1.2 ± 0.3	>50	-
15			4	-	-	-	>50	-
16	E	**SS**	1	-	-	-	>10	-
17			2	-	-	-	>10	-
18			3	10	(>10)	-	>10	-
19			4	-	-	-	5.0 ± 0.5	1.2 ± 0.2
20	F	**dMAC**	1	<0.6	13 ± 2	0.5 ± 0.1	>50	-
21			2	<0.6	9 ± 1	0.4 ± 0.1	>50	-

22			3	1	11 ± 4	0.7 ± 0.2	>50	-
23			4	2	28 ± 3	0.6 ± 0.1	>50	-
24	G	**CTO**	1	5	40 ± 20	0.8 ± 0.2	>50	-
25			2	4	(60 ± 20)	0.6 ± 0.2	>50	-
26			3	2	10 ± 1	0.9 ± 0.1	>50	-
27			4	-	-	-	>50	-
28	H	**AspA**	1	5	(40 ± 15)	0.8 ± 0.2	>50	-
29			2	3	>50	-	>50	-
30			3	20	(100 ± 25)	1.0 ± 0.2	>50	-
31			4	-	>50	-	>50	-
32	I	**ETP**	1	3	>10	-	15 ± 2	5 ± 2
33			2	<0.6	9.0 ± 0.5	0.5 ± 0.1	(80 ± 10)	2.0 ± 0.5
34			3	1.2	(30 ± 10)	0.6 ± 0.2	~40	-
35			4	1.2	(30 ± 10)	3.0 ± 1.5	>25	-
36	J	**BPS**	1	0.4	9 ± 2	0.6 ± 0.1	18 ± 2	5 ± 2
37			2	0.7	9 ± 1	0.7 ± 0.1	>25	-
38			3	3	(10 ± 3)	1.3 ± 0.5	>10	-
39			4	0.3	7 ± 1	0.6 ± 0.1	>25	-

[a]Results from dose-response curves in Figure S22. [b]Results corresponding to the graphs above. [c]Inhibitor. [d]Cell line used 1: MDA-MB-231; 2: A-431; 3: RPE-1; 4: MCF-7. [e]Concentration needed to reach 15% inhibition. [f]Concentration needed to reach 50% inhibition. [g]Hill coefficient for inhibition of cellular uptake. [h]Concentration needed to lower relative viability (RV) by 50%. [i]Hill coefficient for cell viability.

7. AHCHT Screening for OPS-Cy5

7.1 General Experimental Procedure for OPS-Cy5 Uptake

As described in section 5.1. Cells were prepared in a 96 well plate as described in section 4, then medium was removed, and cells were washed with PBS (3 × 3 mL/well) followed by fresh serum free FDMEM medium (4 × 100 µL/well) using a plate washer (Biotek EL406®), and kept in a 100 µL of the latter medium. The solution of **OPS-Cy5** (100 µM, TRIS buffer, 10 mM, pH = 8.0 with 1 mM EDTA) was diluted in FDMEM to give a solution at 3x final concentration, of which 50 µL was added to the well resulting in a final volume of 150 µL per well. The cells were incubated under 5% CO_2 humidified atmosphere at 37 °C for the indicated time (30 – 240 minutes). Afterward, to remove the excess of fluorescent transporter, the cells were washed with PBS and the medium was exchanged with FDMEM keeping a final volume of 100 µL/well, and a solution of Hoechst 33342 (100 µg/mL) and PI (10 µg/mL) in PBS (15 µL/well) was added. After 10 min of incubation under 5% CO_2 humidified atmosphere at 37 °C, cells were washed with PBS (3 × 3 mL/well) and then fixed by treating with a solution of 5% PFA for 15 min at rt. The fixed cells were washed with PBS (9 × 3 mL/well) and imaged on a IXM-C automated microscope using a 20X WI lens with three channels, blue for Hoechst 33342 (377/50 nm excitation filter; 477/60 nm emission filter), red for PI (531/40 nm excitation filter; 593/40 nm emission filter) and far red for **OPS-Cy5** transporter (620/50 nm excitation filter; 690/50 nm emission filter). Duplicates were performed for each condition.

7.2 Data Analysis

Images were automatically analyzed and quantified using a protocol described in section 5.1 with modifications. Namely, after removing dead/dying cells, aggregates and border cells, the puncta in cells were detected in the red channel. To facilitate the detection, a *Top-hat* transformation of the Cy5 red channel was applied, then a round object module was used to segment all puncta. The final mask allowed to quantify the Cy5 signals in the cytoplasm excluding the puncta (magenta in Figure S23).

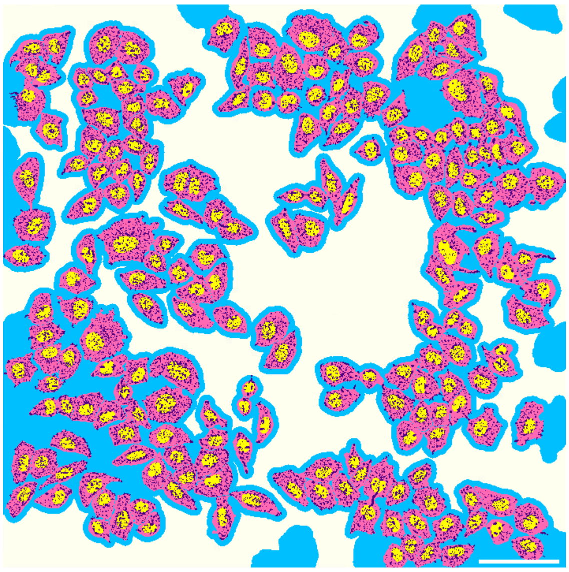

Figure S23. Final mask: yellow – nuclei; magenta – cytoplasm; cyan – inter-cellular space; blue – puncta; beige – background. Scale bar 100 μm.

The quantification was performed using the protocols described in section 5.2.

8. Uptake and Inhibition Studies in Integrin Knockdown HK Cells

8.1. Knockdown Procedure

For the knockdown of integrin β subunits, reverse transfection method was performed with a final concentration of 5 nM for siRNAs ITGB1, ITGB5 and non-target control, and 3 nM for siRNA ITGB3, based on the procedure of transfection provided by siTOOLs Biotech. Briefly, siRNA diluted in reduced serum medium Opti-MEM was pre-mixed with Lipofectamine RNAiMAX (Thermo Fisher Scientific) in Opti-MEM for 15 minutes at room temperature. The pre-mixed solution of siRNA was transferred to a 6-well sterile plate (500 µL/well). HeLa Kyoto cells were then seeded at 3×10^5 cells/well and incubated under 5% CO_2 humidified atmosphere at 37 °C for 48 h. Afterward, cells were detached by treatment with 500 µL of TrypLE Express at 37 °C for 5 min, followed by the addition of 1 mL of complete FDMEM (supplemented with FCS and PS). The cells were spun down at 1500 rpm for 3 min, re-suspended in complete FDMEM medium, and seeded at 12×10^3 cells/well in complete FDMEM in µ-Plate 96-well Black ibiTreat sterile and kept under 5% CO_2 humidified atmosphere at 37 °C overnight.

8.2. Protein Knockdown Quantification by Immunofluorescence

After knocking down the corresponding protein (section 8.1), the cells were washed with PBS (9 x 3 mL/well) and fixed with a solution of 5% PFA for 15 min at rt. The cells were washed with PBS (9 x 3 mL/well) and treated for 1 h at rt (or overnight at 4 °C for ITGB5) with mouse monoclonal anti-integrin β1, β5 antibodies in PBS containing 1% BSA and 0.05% saponin (70 µL per well, 1/200 of 0.5 mg/mL antibody). The cells were washed with PBS (9 × 3 mL/well) using the plate washer and treated with a solution of the secondary antibody with a fluorescent dye in PBS (80 µL per well, 1/400 of 1 mg/mL; Alexa Fluor® 647 AffiniPure Donkey Anti-Mouse IgG (H+L)). The cells were washed with PBS (9 × 3 mL/well) and then imaged on a IXM-C automated microscope acquiring 9 images per well using a 20X-WI objective lens with 2 channels, blue for Hoechst 33342 (377/50 nm excitation filter; 477/60 nm emission filter) and red for the secondary antibody (620/50 nm excitation

filter; 690/50 nm emission filter). The fluorescence intensity of the red channel was extracted and analyzed as described in section 5.2.

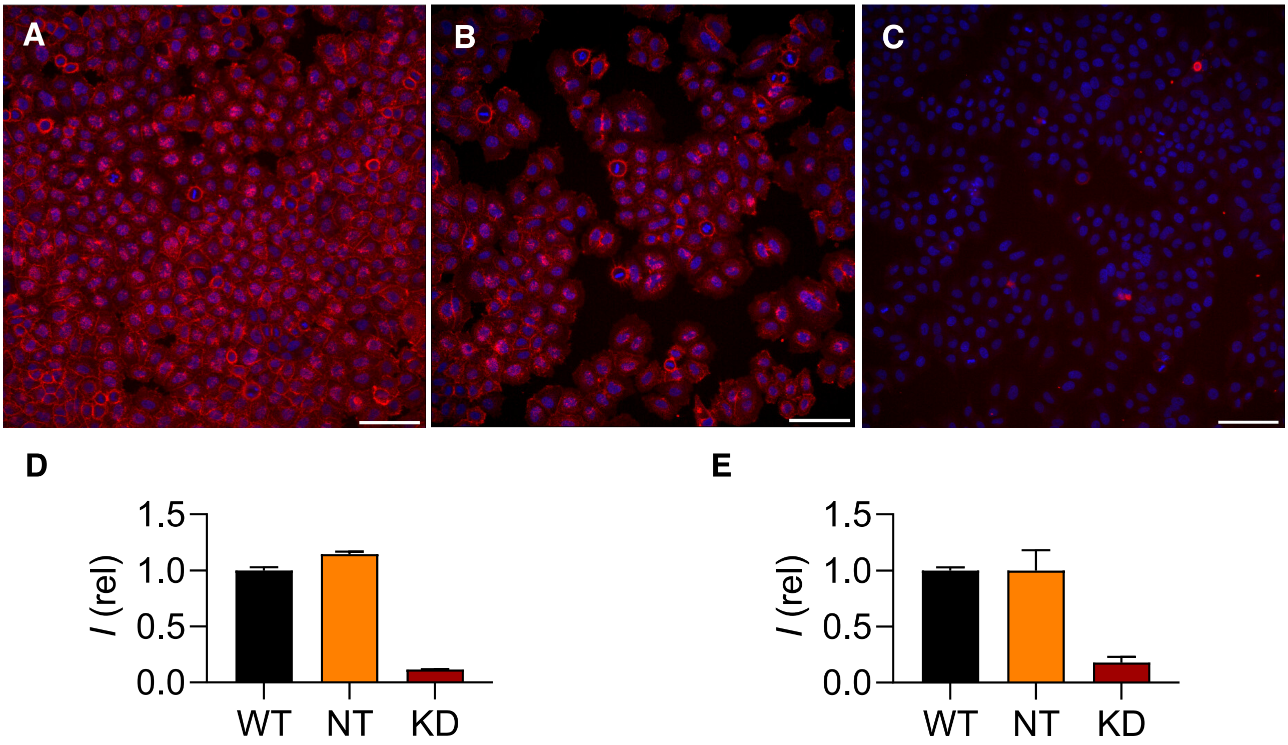

Figure S24. SDCM images (20X WI) for quantification of ITGB1 in A) wild-type, B) non-target, and C) ITGB1 knockdown HK cells by immunofluorescence (red; blue: Hoechst 33342, nuclei; scale bar 100 µm). Relative fluorescence intensity *I* (rel) ± SEM of KD HK cells for D) ITGB1 KD and E) ITGB5 KD (WT: wild-type, NT = non-target).

8.3. Cellular Uptake in Integrin Knocked-Down Cells

8.3.1. Fl-CAXs

As described in section 5 using integrin KD HK cells.

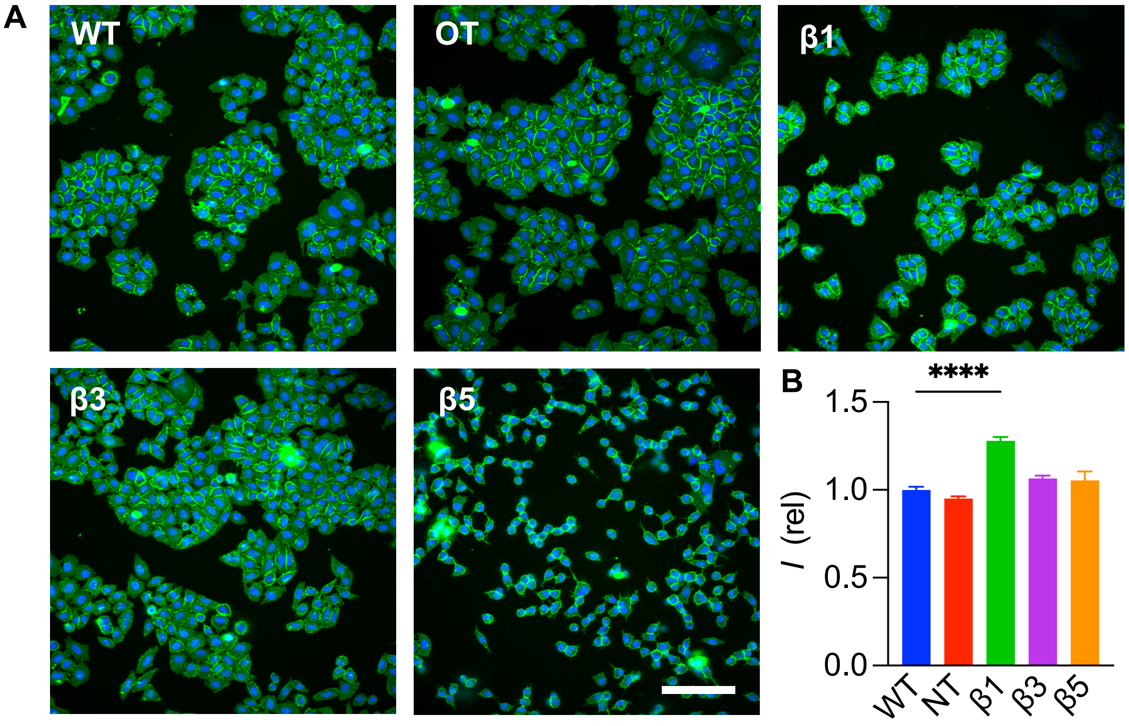

Figure S25. A) SDCM images (20X WI) showing fluorescence intensity of **FI-BPS** (5 µM, green) in KD HK cells after incubation for 1 h (blue: Hoechst 33342, nuclei; scale bar 150 µm). B) Resulting relative fluorescence intensity *I* (rel) ± SEM (WT: wild-type, NT: non-target). **** $P < 0.0001$ obtained with two-tailed unpaired t-test. Cell count average: 300–200 cells.

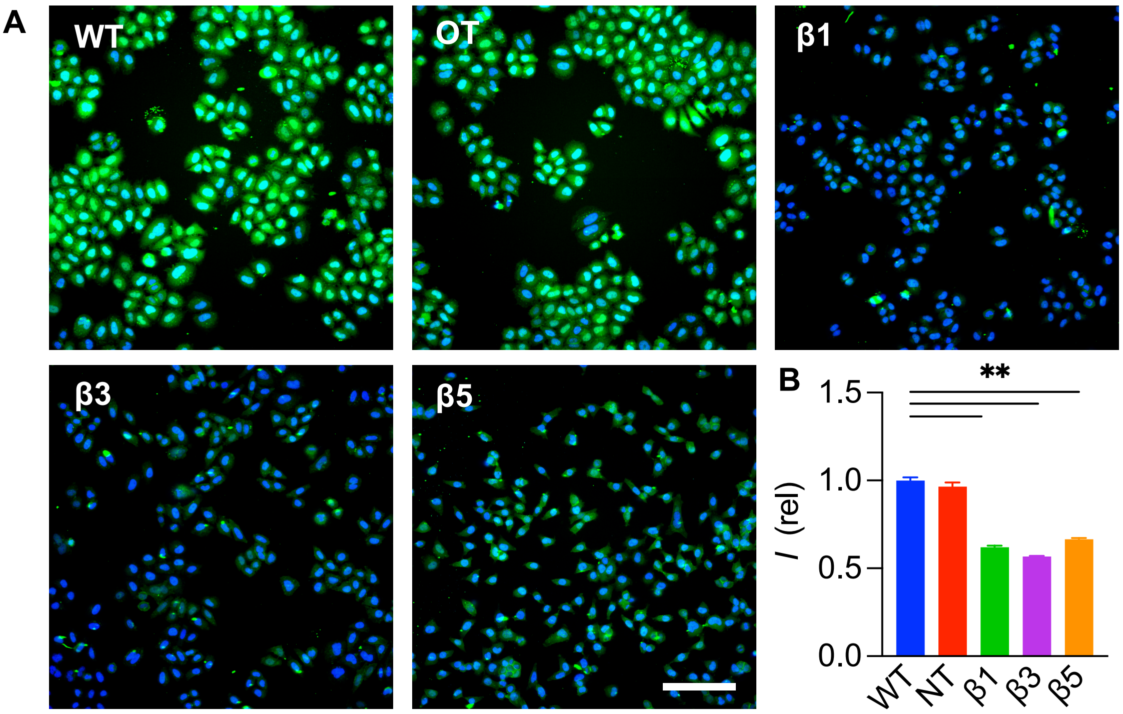

Figure S26. A) SDCM images (20X WI) showing fluorescence intensity of **FI-ETP** (5 µM, green) in KD HK cells after incubation for 1 h (blue: Hoechst 33342, nuclei; scale bar 150 µm). B) Resulting relative fluorescence intensity *I* (rel) ± SEM (WT: wild-type, NT: non-target). $^{**}P < 0.01$ obtained with two-tailed unpaired t test. Cell count average: 250 cells.

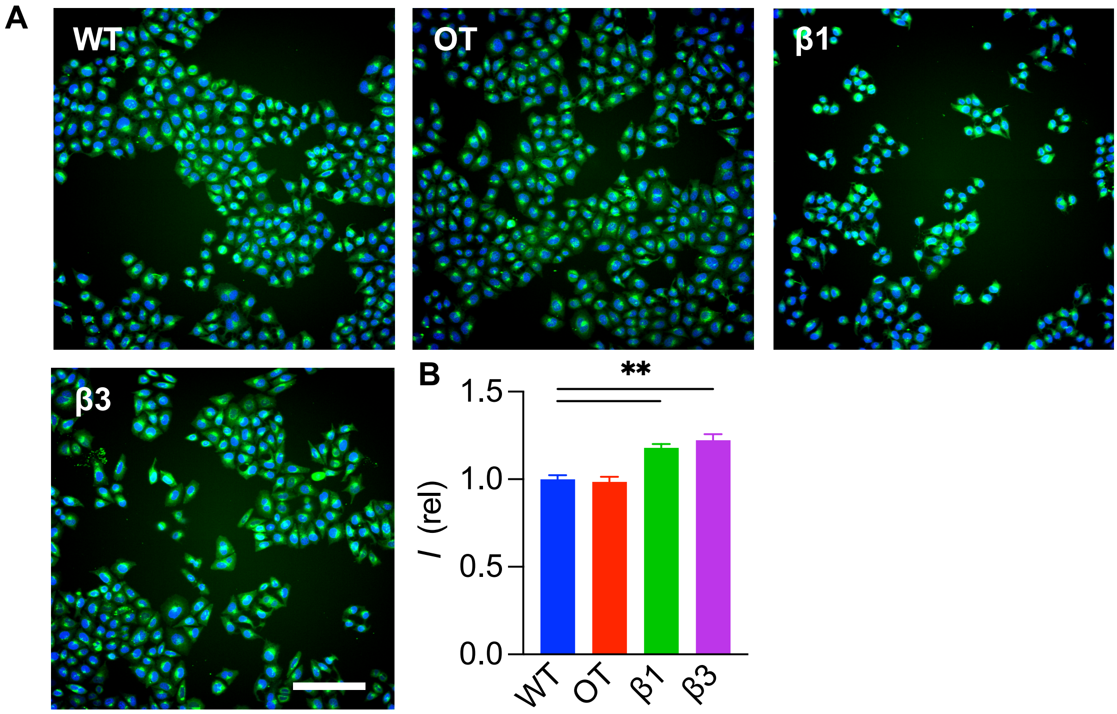

Figure S27. A) SDCM images (20X WI) showing fluorescence intensity of **FI-AspA** (10 μM, green) knockdown in HK cells after incubation with for 1 h (blue: Hoechst 33342, nuclei; scale bar 150 μm). B) Resulting relative fluorescence intensity *I* (rel) ± SEM (WT: wild-type, NT: non-target). ***P* < 0.01 obtained with two-tailed unpaired t test. Cell count average: 300-250 cells.

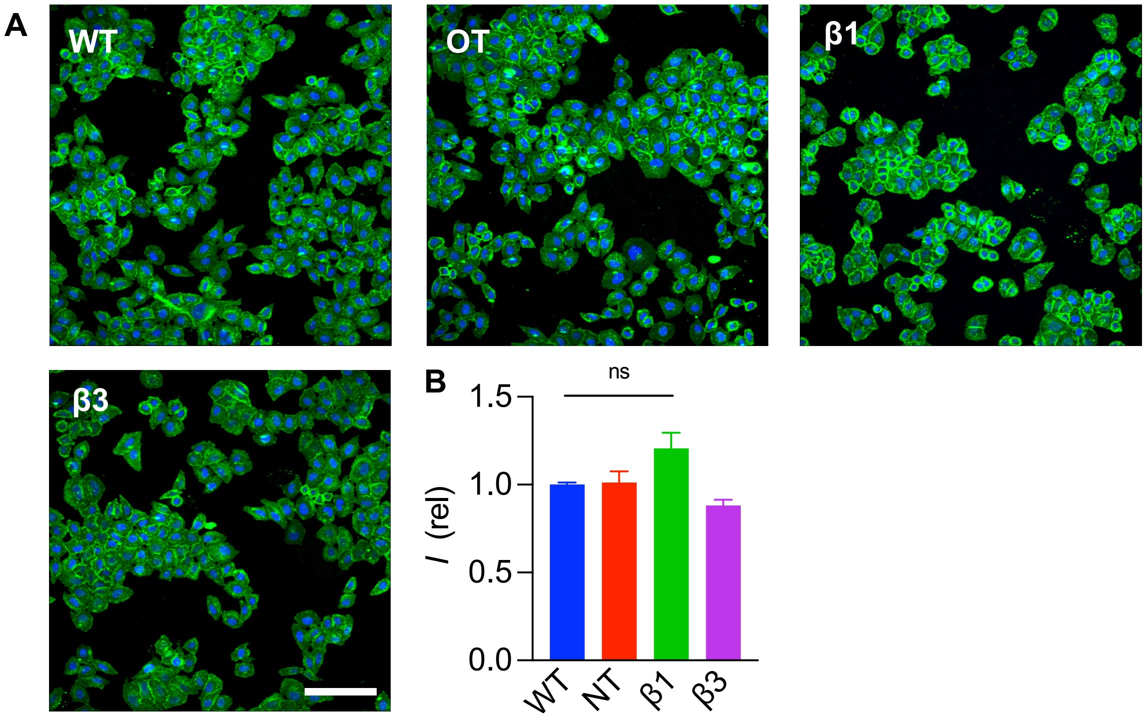

Figure S28. A) SDCM images (20X WI) showing fluorescence intensity of **FI-CTO** (5 µM, green) in KD HK cells after incubation for 1 h (blue: Hoechst 33342, nuclei; scale bar 150 µm). B) Resulting relative fluorescence intensity *I* (rel) ± SEM (WT: wild-type, NT: non-target). ns: not significant. Cell count average: 200-250 cells.

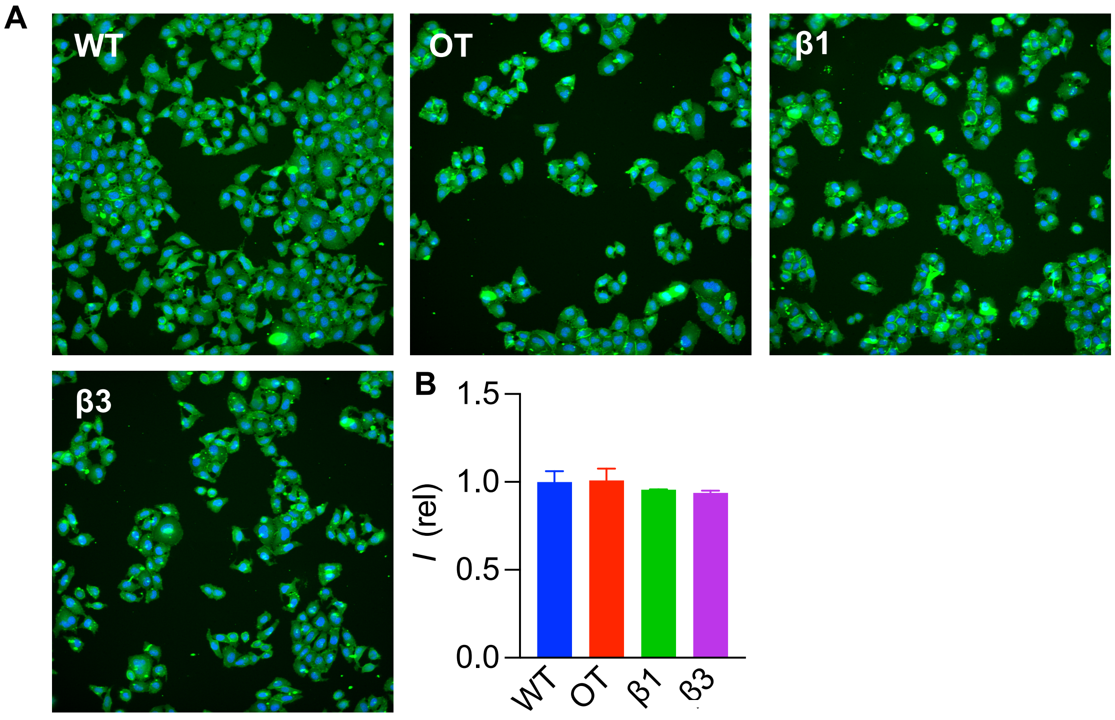

Figure S29. A) SDCM images (20X WI) showing fluorescence intensity of **FI-MAC** (10 µM, green) in KD HK cells after incubation for 1 h (blue: Hoechst 33342, nuclei; scale bar 150 µm). B) Resulting relative fluorescence intensity *I* (rel) ± SEM (WT: wild-type, NT: non-target). Cell count average: 200-160 cells.

8.3.2. OPS-Cy5

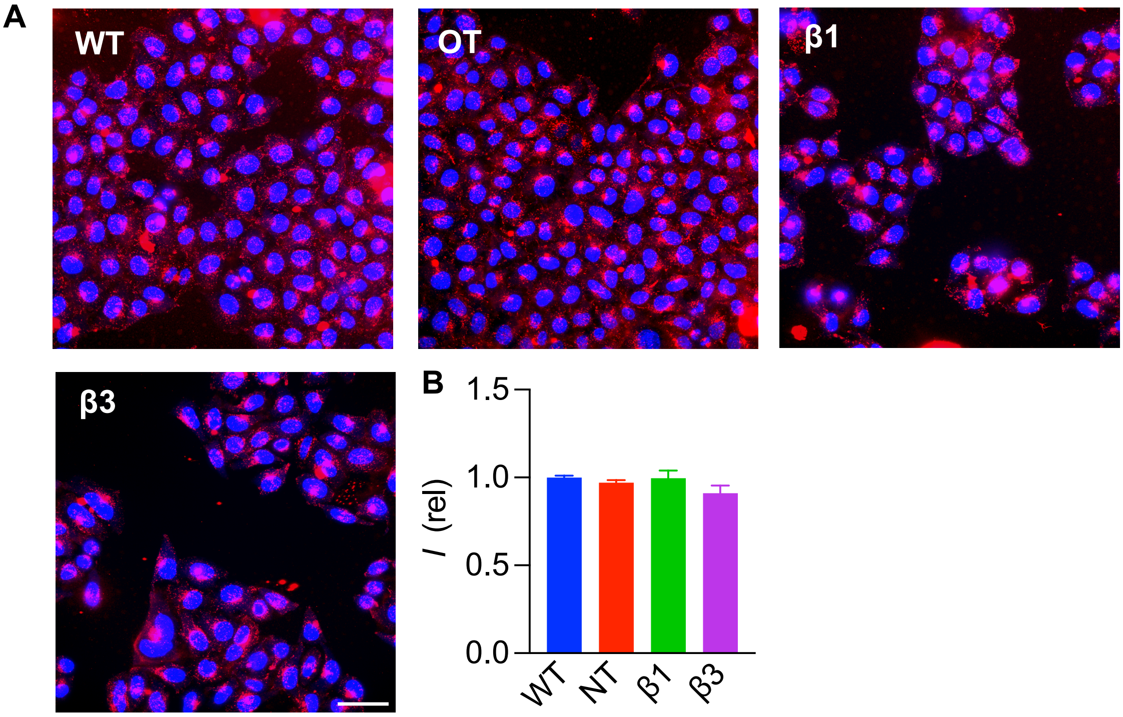

Figure S30. A) SDCM images (20X WI) showing fluorescence intensity of **OPS-Cy5** (0.5 µM, red) in KD HK cells after incubation with for 1 h (blue: Hoechst 33342, nuclei; scale bar 150 µm). B) Resulting relative fluorescence intensity *I* (rel) ± SEM (WT: wild-type, NT: non-target). Cell count average: 200-160 cells.

8.4. Inhibitor Screening in Knocked-down Cells

Inhibitor screening was performed using the co-incubation method with knocked-down cells following the procedure described in section 6 with modified incubation time of the transporter of 45 min.

8.4.1. Fl-BPS Transporter

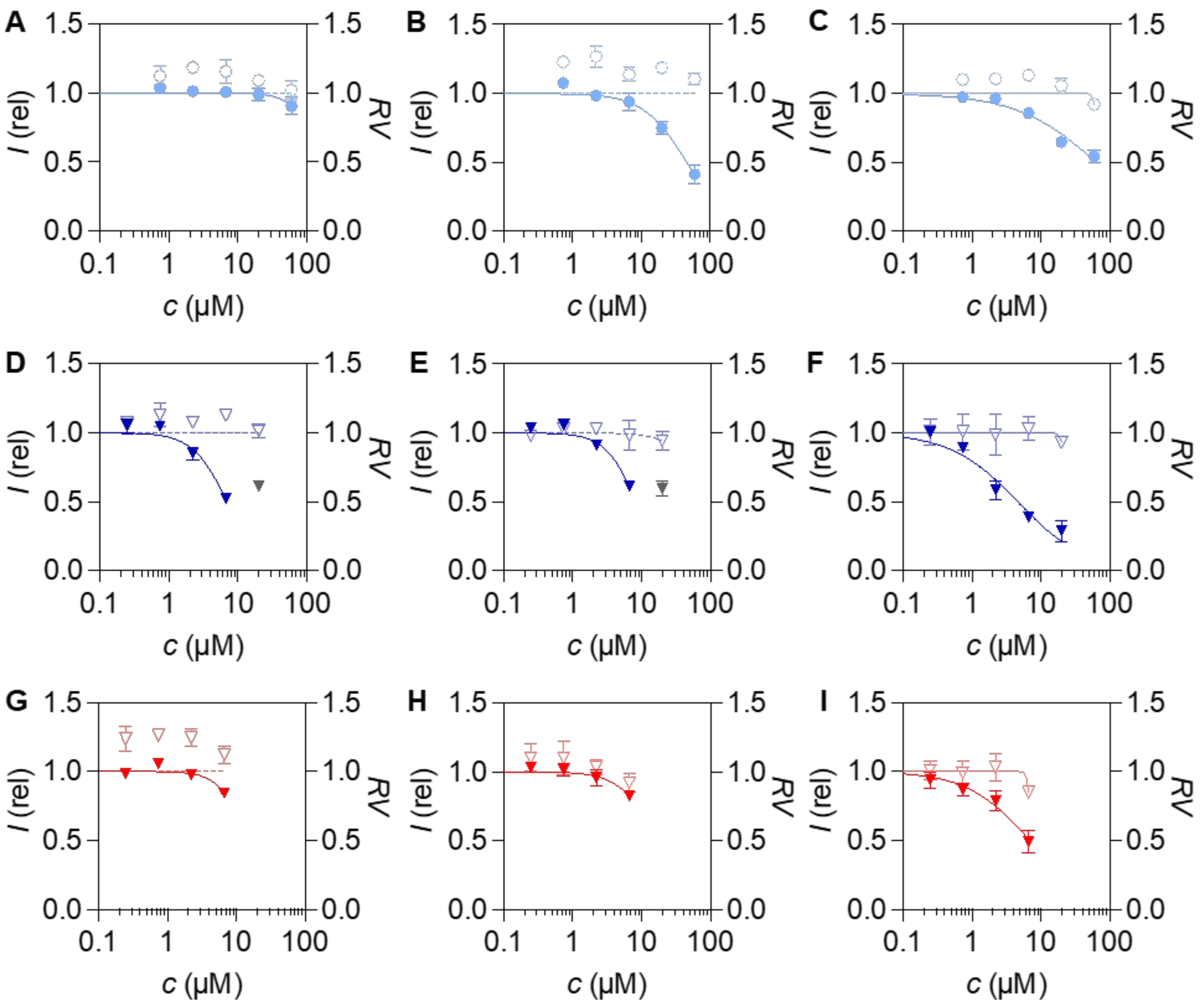

Figure S31. Relative fluorescence intensity I (rel) ± SEM (filled symbols) of **Fl-BPS** (5 μM) and relative viability RV ± SEM (empty symbols) as a function of the concentration of (A–C) **EBS,** (D–F) **BiC**, and (G–I) **ETP** in (A, D, G) OT, (B, E, H) ITGB1 and (C, F, I) ITGB3 siRNA treated HK cells. Grey symbols represent the data points excluded from the curve fit.

Table S11. Dependence of cellular uptake of **Fl-BPS** (5 μM) and cell viability in siRNA treated HK cells on the concentration of CAX inhibitors under co-incubation condition.[a]

Entry	I[c]	siRNA[d]	MIC (μM)[e]	IC_{50} (μM)[f]	*n* (IC_{50})[g]	RV_{50} (μM)[h]	*n* (RV_{50})[i]
1	**EBS**	OT	-	>>50	-	>75	-
2		ITGB1	15	45 ± 5	1.4 ± 0.2	>75	-
3		ITGB3	6	65 ± 10	0.7 ± 0.1	>75	-
4	**BiC**	OT	3	(10)	-	>20	-
5		ITGB1	3	(10)	-	>20	-
6		ITGB3	0.6	5 ± 1	0.9 ± 0.1	>20	-
7	**ETP**	OT	5	(15)	-	>7	-
8		ITGB1	5	(15)	-	>7	-
9		ITGB3	1	7 ± 2	0.9 ± 0.2	>7	-

[a]Results from dose-response curves in Figure S31. [b]Inhibitor. [c]siRNA used for the knockdown HK cells. [d]Concentration needed to reach 15% inhibition. [e]Concentration needed to reach 50% inhibition. [f]Hill coefficient for inhibition of cellular uptake. [g]Concentration needed to lower relative viability (RV) by 50%. [h]Hill coefficient for cell viability.

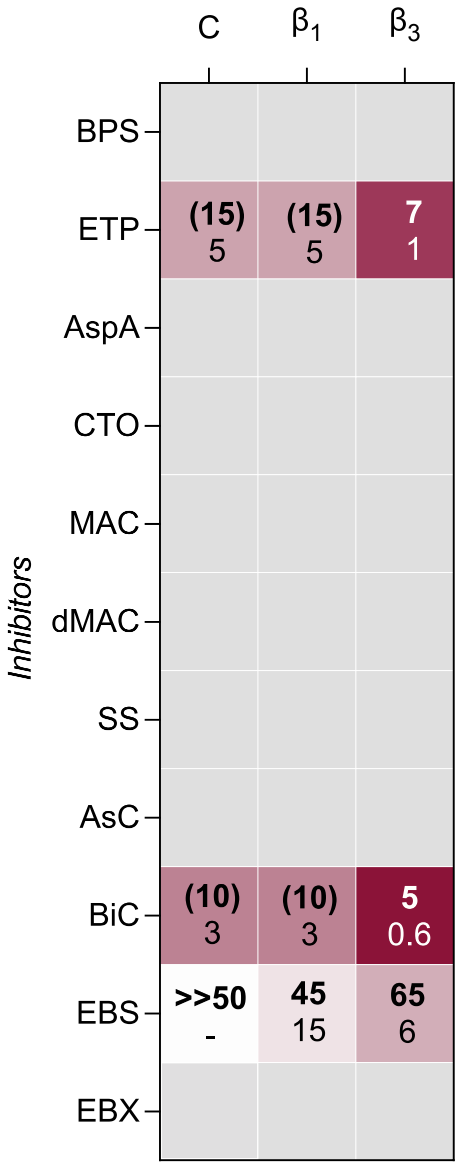

Figure S32. Heatmap for the MIC of candidates to inhibit cellular uptake of **Fl-BPS** in non-target, siITGB1 and siITGB3 treated HK cells.

8.4.2. Fl-ETP Transporter

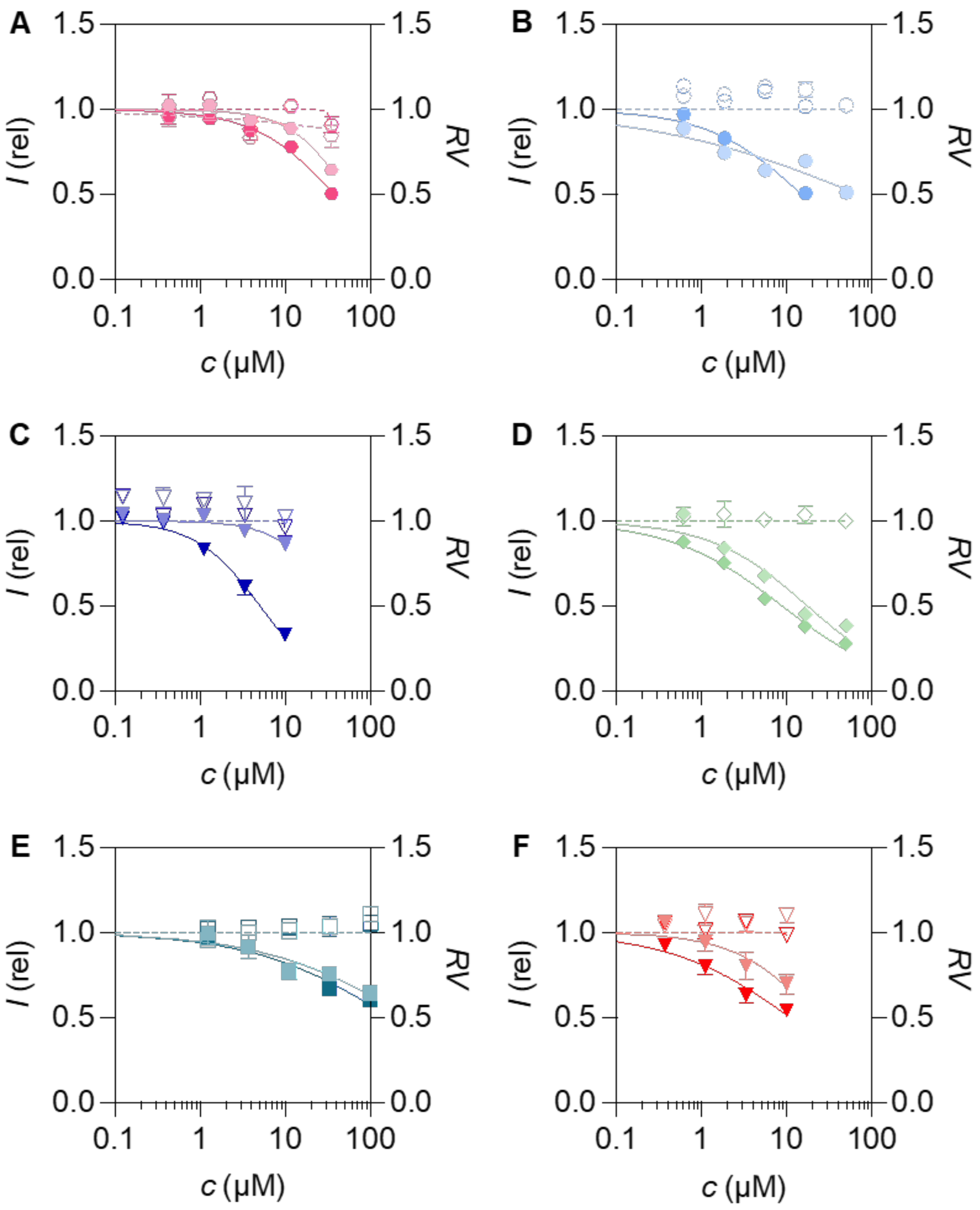

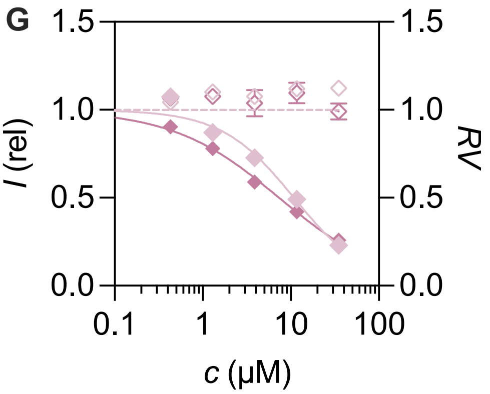

Figure S33. Relative fluorescence intensity *I* (rel) ± SEM (filled symbols) of **FI-ETP** (5 µM) and relative viability *RV* ± SEM (empty symbols) in siRNA treated HK cells as a function of the concentration of A) **EBX**, B) **EBS**, C) **BiC**, D) **dMAC**, E) **MAC**, F) **CTO**, G) **ETP** and H) **BPS**. Dark color: non-target, lighter color: siITGB1.

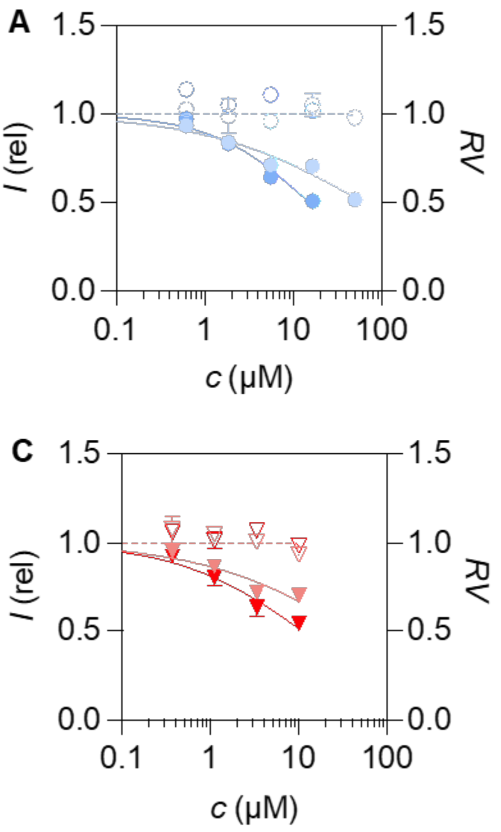

Figure S34. Relative fluorescence intensity *I* (rel) ± SEM (filled symbols) of **Fl-ETP** (5 µM) and relative viability *RV* ± SEM (empty symbols) in siRNA treated HK cells as a function of the concentration of A) **EBS**, B) **BiC** and C) **ETP**. Dark color: non-target, lighter color: siITGB3.

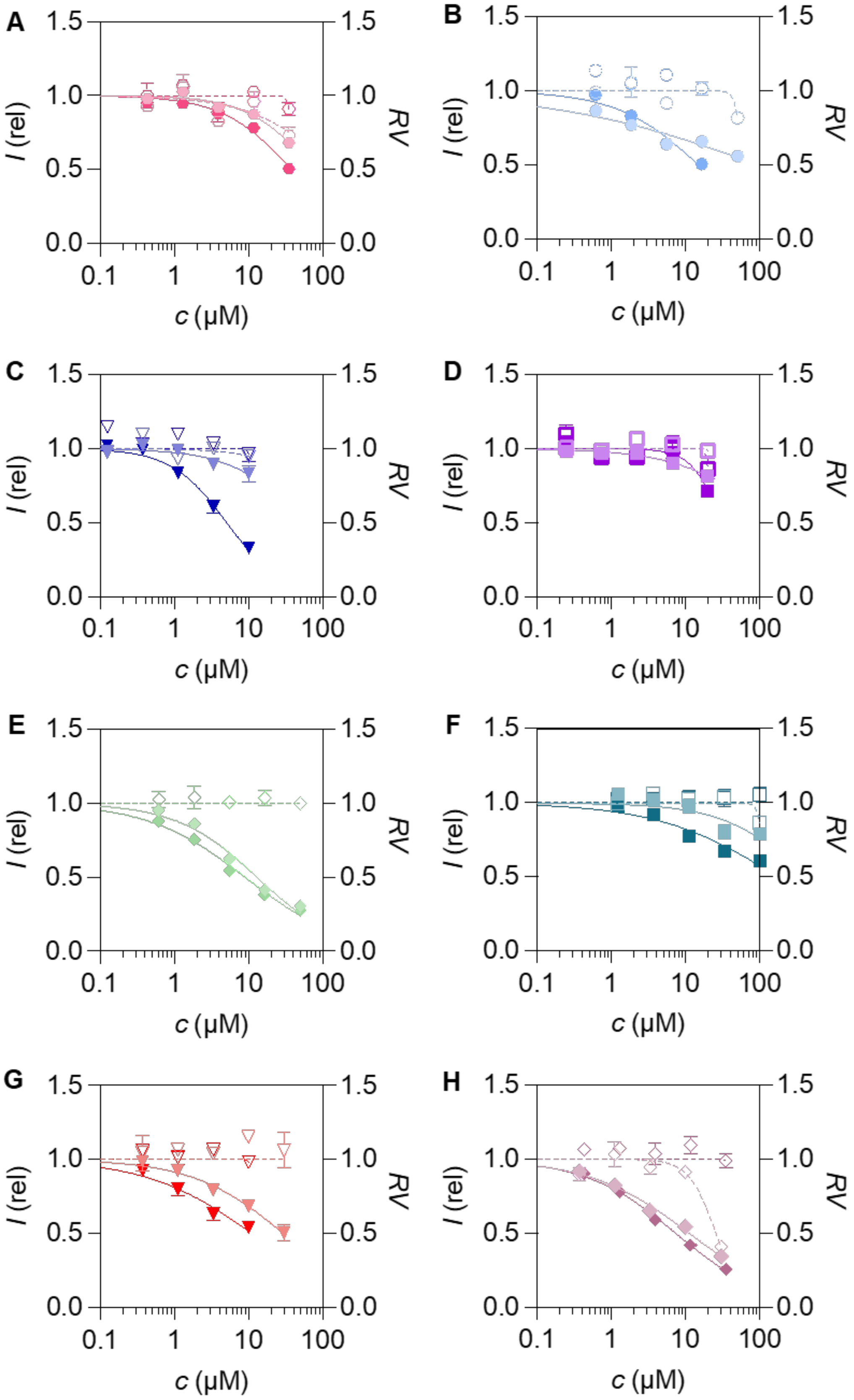
A
B
C
D
E
F
G
H
1.5
1.0
0.5
0.0
I (rel)
RV
0.1
1
10
100
c (μM)

Figure S35. Relative fluorescence intensity *I* (rel) ± SEM (filled symbols) of **Fl-ETP** (5 μM) and relative viability *RV* ± SEM (empty symbols) in siRNA treated HK cells as a function of the concentration of A) **EBX**, B) **EBS**, C) **BiC**, D) **AsC**, E) **dMAC**, F) **MAC** G) **ETP** and H) **BPS**. Dark color: non-target, lighter color: siITGB5.

Table S12. Dependence of cellular uptake of **Fl-ETP** (5 μM) and cell viability in siRNA treated HK cells on the concentration of CAX inhibitors under co-incubation condition. (Part I)[a]

Entry	I[c]	siRNA[d]	MIC (μM)[e]	IC_{50} (μM)[f]	n (IC_{50})[g]	RV_{50} (μM)[h]	n (RV_{50})[i]
1	**EBX**	OT	6	(40)	-	>35	-
2		ITGB1	15	(55)	-	>35	-
3		ITGB5	10	(75)	-	>35	-
4	**EBS**	OT	2	15 ± 2	1.0 ± 0.1	>50	-
5		ITGB1	<0.6	(70)	-	>50	-
6		ITGB3	1	(60)	-	>50	-
7		ITGB5	<0.6	(100)	-	>50	-
8	**BiC**	OT	1	5.0 ± 0.5	1.0 ± 0.1	>10	-
9		ITGB1	-	(40)	-	>10	-
10		ITGB3	4	(20)	-	>10	-
11		ITGB5	6	(60)	-	>10	-
12	**AsC**	OT	15	(30)	-	>20	-
13		ITGB5	12	(150)	-	>20	-

[a]Results from dose-response curves in Figures S33, S34 and S35. [b]Inhibitor. [c]siRNA used for the knockdown HK cells. [d]Concentration needed to reach 15% inhibition. [e]Concentration needed to reach 50% inhibition. [f]Hill coefficient for inhibition of cellular uptake. [g]Concentration needed to lower relative viability (RV) by 50%. [h]Hill coefficient for cell viability.

Table S13. Dependence of cellular uptake of **Fl-ETP** and cell viability in siRNA treated HK cells on the concentration of CAX inhibitors under co-incubation condition. (Part II)[a]

Entry	I[b]	siRNA[c]	MIC (μM)[d]	IC_{50} (μM)[e]	*n* (IC_{50})[f]	RV_{50}(μM)[g]	*n* (RV_{50})[h]
1	**dMac**	OT	1	10 ± 1	1.0 ± 0.1	>50	-
2		ITGB1	2	20 ± 3	1.0 ± 0.3	>50	-
3		ITGB5	1	15	-	>50	-
4	**Mac**	OT	7	(170)	-	>100	-
5		ITGB1	10	(280)	-	>100	-
6		ITGB5	50	(420)	-	>100	-
7	**ETP**	OT	1	8 ± 1	1 ± 0.1	>10	-
8		ITGB1	5	(30)	-	>15	-
9		ITGB3	1	(40)	-	>10	-
10		ITGB5	2	(30)	-	>10	-
11	**BPS**	OT	1	7 ± 1	1.0 ± 0.1	>35	-
12		ITGB1	2	10 ± 1	1.0 ± 0.1	>40	-
13		ITGB5	1	10 ± 1	0.60 ± 0.04	25 ± 2	2.0 ± 0.5

[a]Results from dose-response curves in Figures S33, S34 and S35. [b]Inhibitor. [c]siRNA used for the knockdown HK cells. [d]Concentration needed to reach 15% inhibition. [e]Concentration needed to reach 50% inhibition. [f]Hill coefficient for inhibition of cellular uptake. [g]Concentration needed to lower relative viability (RV) by 50%. [h]Hill coefficient for cell viability.

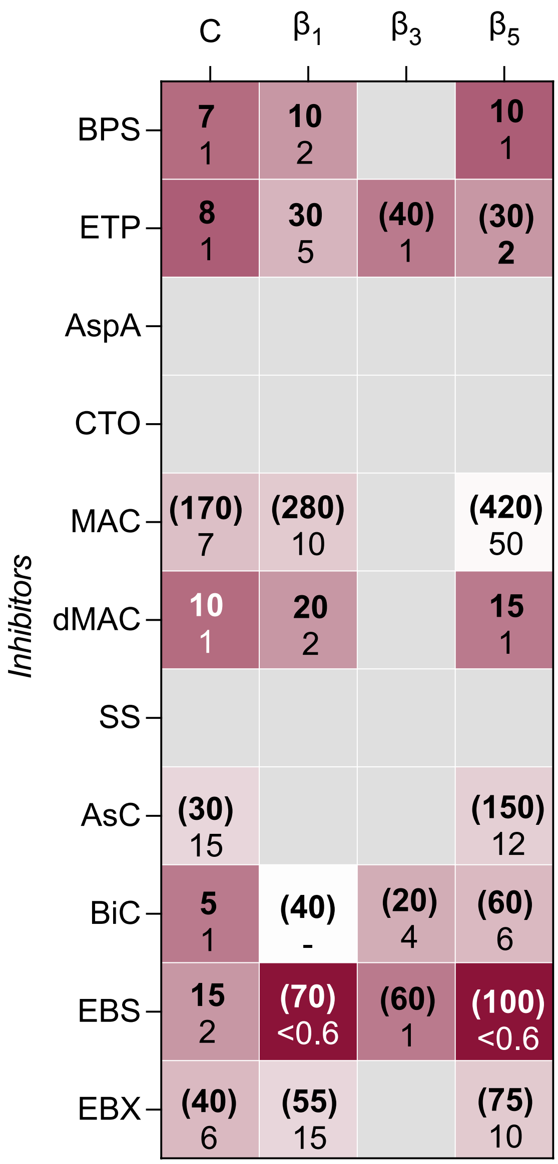

Figure S36. Heatmap for the MIC of candidates to inhibit cellular uptake of **Fl-ETP** in non-target, siITGB1, siITGB3 and siITGB5 treated HK cells.

9. Effects of PDI Inhibitors

9.1. LC-MS Analysis of Transporters in Presence of PDI Inhibitors

The **Fl-ETP** or **Fl-BPS** transporter (5 µL, 100 µM) was diluted in FDMEM (100 µL) in presence or absence of **16F16** (10 µL, 500 µM) or **LOC14** (10 µL, 500 µM). The mixture was shaken at 800 rpm at rt for 30 min. The mixture was analyzed by LC-MS. Conditions: column Hypersil Gold Vanquish 1.9 µm 2.1 x 50 mm, gradient: 5-95% CH_3CN in H_2O + 0.1% Formic acid in 4 min, 0.5 mL/min. Detection wavelength 220 nm.

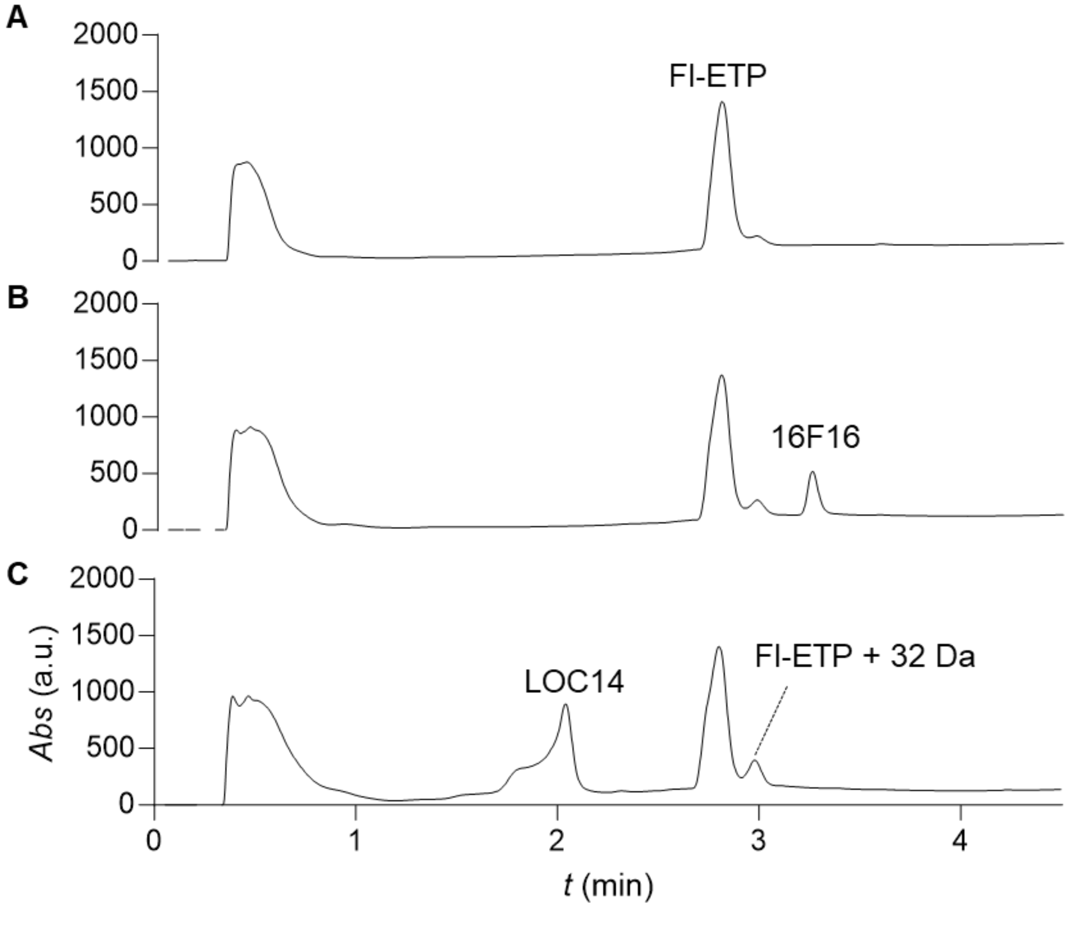

Figure S37. HPLC profiles of **Fl-ETP** transporter after 30 minutes of incubation in FDMEM A) without PDI inhibitors, B) with **16F16** and C) **LOC14**.

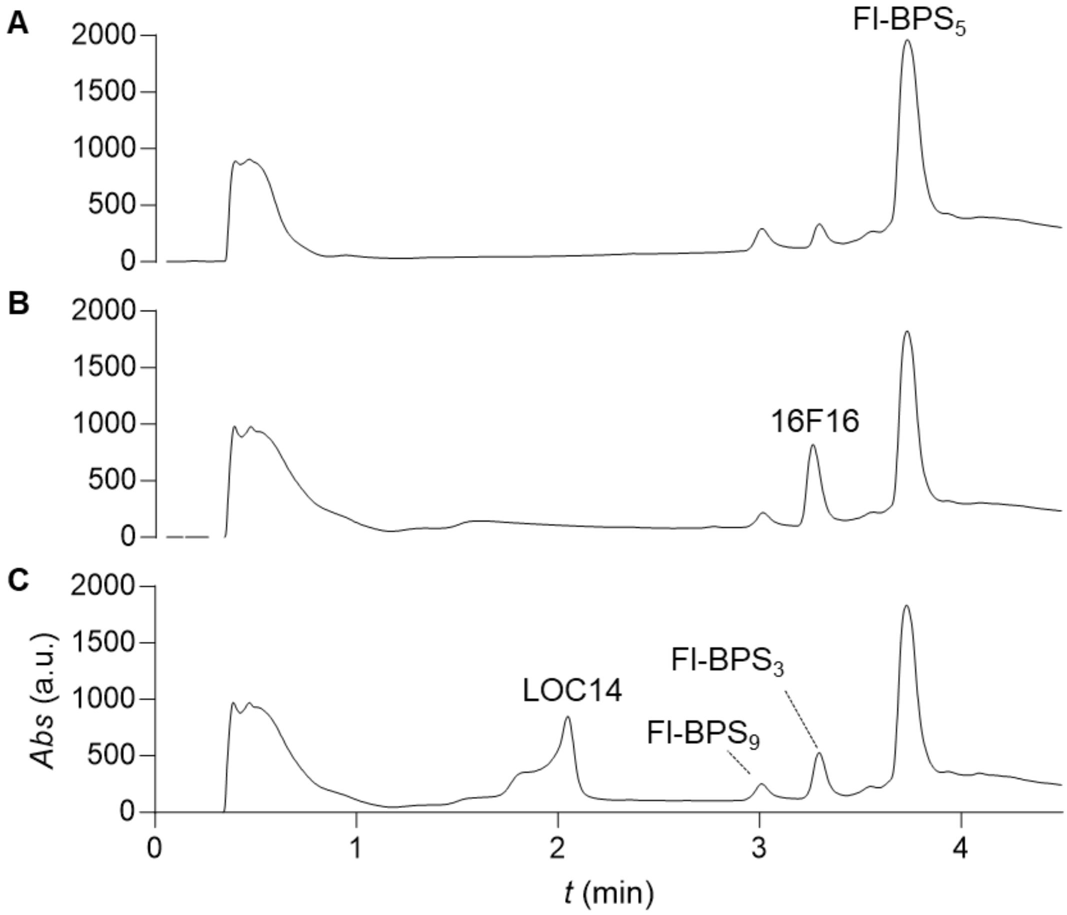

Figure S38. HPLC spectra of **Fl-BPS** transporter after 30 minutes of incubation in FDMEM A) without PDI inhibitors, B) with **16F16** and C) **LOC14**.

9.2. Cellular Uptake in the Presence of PDI Inhibitors

The procedure was as described for co-incubation method in section 6 and 7 using PDI inhibitors instead of TMU inhibitors, with longer incubation time of the transporter for 60 min.

9.2.1. Effects on Fl-BPS Uptake in HK Cells

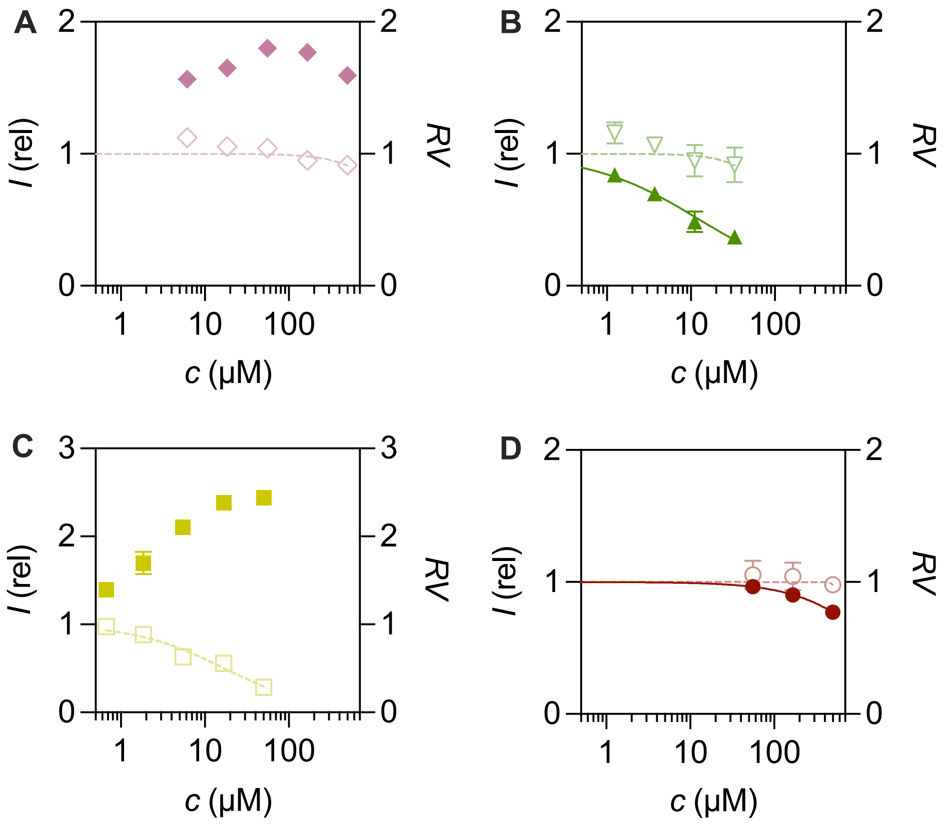

Figure S39. Relative fluorescence intensity I (rel) ± SEM (filled symbols) of **Fl-BPS** (5 µM) and relative viability RV ± SEM (empty symbols) in HK cells as a function of the concentration of A) **16F16**, B) **LOC14**, C) **PACMA** and D) **Rutin**.

Table S14. Dependence of cellular uptake of **Fl-BPS** and cell viability in HK cells on the concentration of PDI inhibitors under co-incubation condition.[a]

Entry	I[b]	MIC (μM)[c]	IC_{50} (μM)[d]	*n* (IC_{50})[e]	RV_{50} (μM)[f]	*n* (RV_{50})[g]
1	**16F16**	-[g]	-	-	>500	-
2	**LOC14**	1	15 ± 1	0.6 ± 0.1	>50	-
3	**PACMA-31**	-	-	-	20 ± 2	0.8 ± 0.1
4	**Rutin**	285	>>500	-	>500	-

[a]Results from dose-response curves in Figure S39. [b]Inhibitor. [c]Concentration needed to reach 15% inhibition. [d]Concentration needed to reach 50% inhibition. [e]Hill coefficient for inhibition of cellular uptake. [f]Concentration needed to lower relative viability (RV) by 50%. [g]Hill coefficient for cell viability.

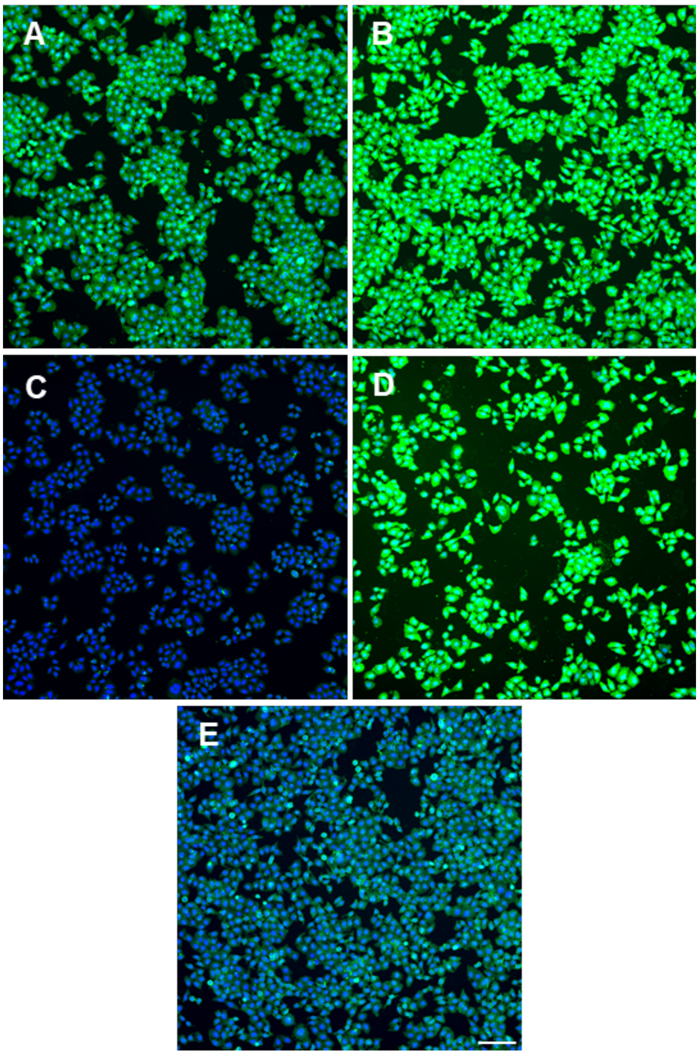

Figure S40. SDCM images (10X, widefield) showing fluorescence intensity of **FI-BPS** (5 µM, green) A) without any inhibitor, or with B) **16F16** (50 µM), C) **LOC14** (50 µM), D) **PACMA-31** (16 µM) and E) **Rutin** (500 µM) (blue: Hoechst 33342, nuclei; scale bar 150 µm). Cell count: 500 cells for PACMA, 1000 cells for all other conditions.

9.2.2. Effects on Fl-ETP Uptake in HK Cells

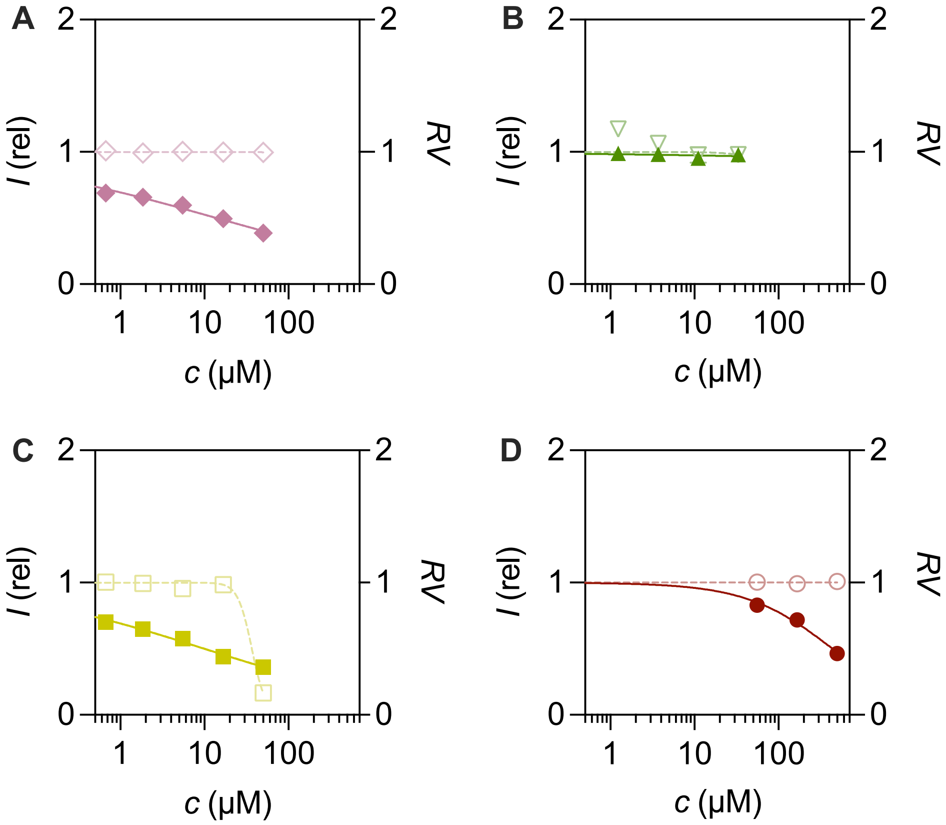

Figure S41. Relative fluorescence intensity *I* (rel) ± SEM (filled symbols) of **Fl-ETP** (10 µM) and relative viability *RV* ± SEM (empty symbols) in HK cells as a function of the concentration of A) **16F16**, B) **LOC14**, C) **PACMA** and D) **Rutin**.

Table S15. Dependence of cellular uptake of **Fl-ETP** and cell viability in HK cells on the concentration of PDI inhibitors under co-incubation condition.[a]

Entry	I[b]	MIC (μM)[c]	IC_{50} (μM)[d]	*n* (IC_{50})[e]	RV_{50} (μM)[f]	*n* (RV_{50})[g]
1	**16F16**	<0.6	15 ± 2	0.30 ± 0.03	>50	-
2	**LOC14**	-	-	-	>50	-
3	**PACMA-31**	<0.6	10 ± 1	0.3 ± 0.1	35 ± 3	5 ± 1
4	**Rutin**	55	440 ± 40	0.8 ± 0.1	>500	-

[a]Results from dose-response curves in Figure S41. [b]Inhibitor. [c]Concentration needed to reach 15% inhibition. [d]Concentration needed to reach 50% inhibition. [e]Hill coefficient for inhibition of cellular uptake. [f]Concentration needed to lower relative viability (RV) by 50%. [g]Hill coefficient for cell viability.

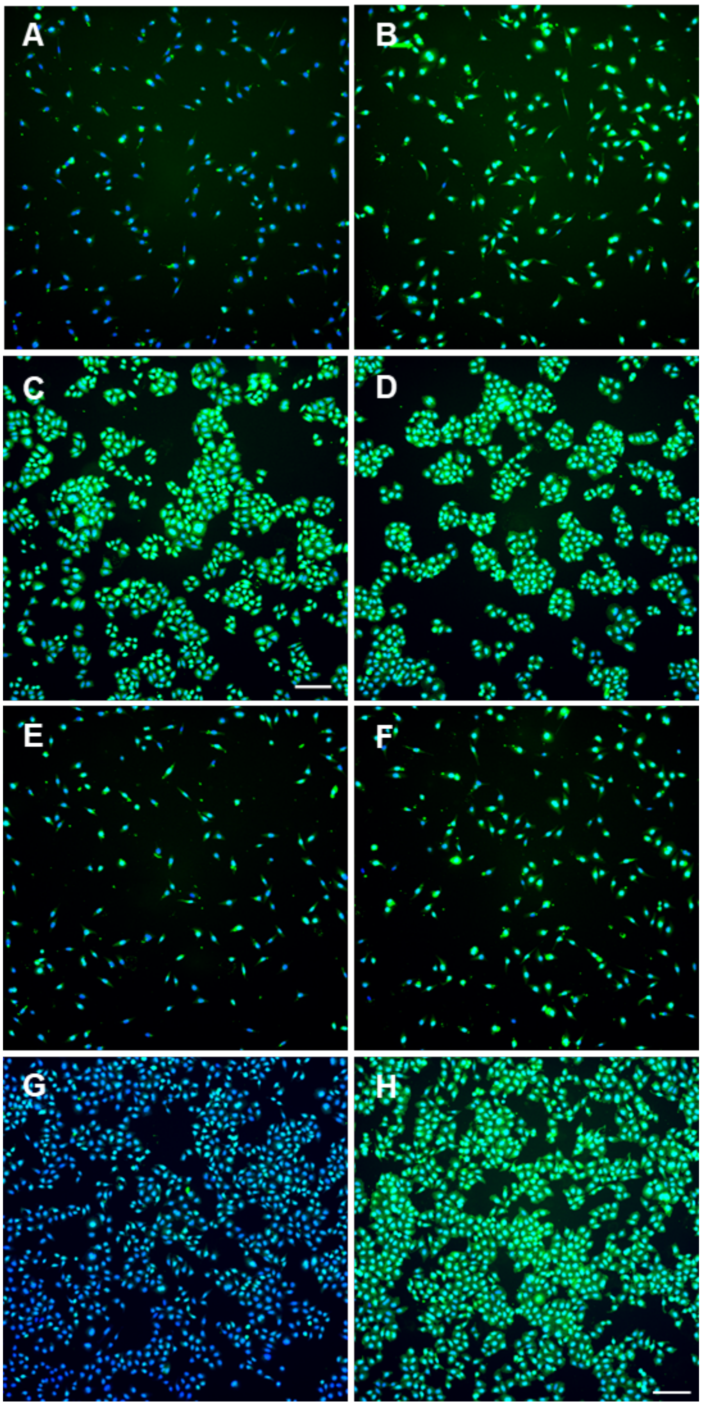

Figure S42. SDCM images (10X, widefield) showing fluorescence intensity (green) of **FI-AspA** (10 µM) A) with or B) without **16F16** (50 µM), C) with or D) without **LOC14** (50 µM), E) with or D) without **PACMA-31** (16 µM) and G) with and H) without **Rutin** (500 µM) (blue: Hoechst 33342, nuclei; scale bar 150 µm). Cell count: 300 cells for 16F16 and PACMA, 1000 cells for all other conditions.

9.2.3. Effects on Fl-AspA Uptake in HK Cells

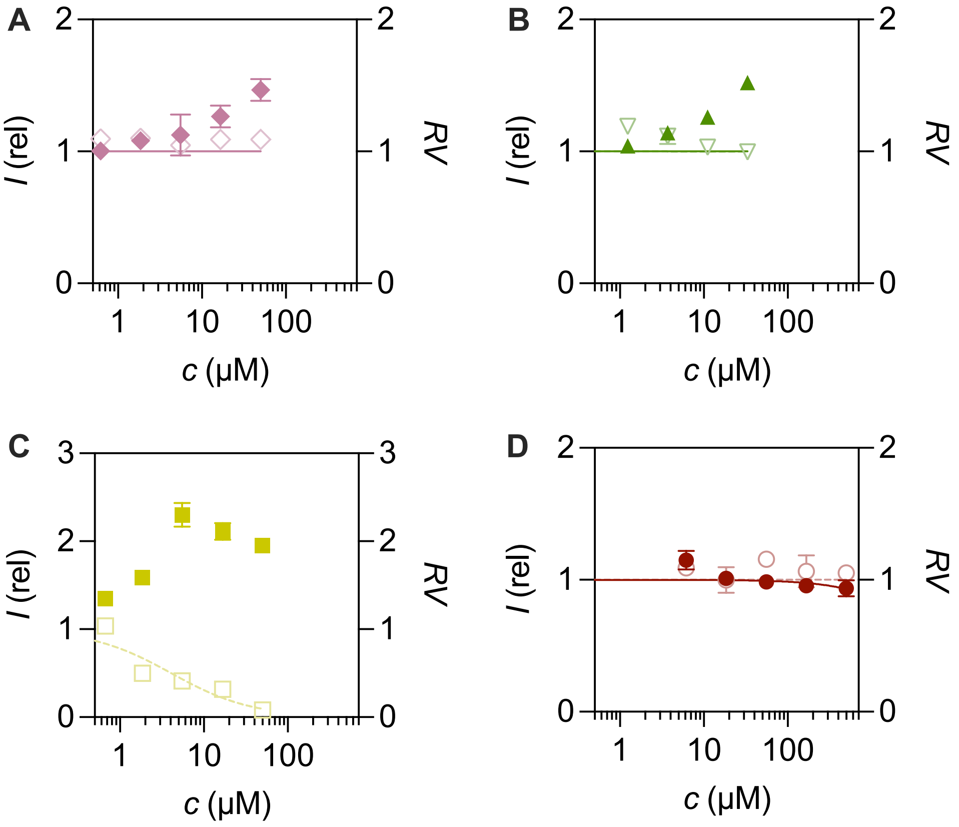

Figure S43. Relative fluorescence intensity *I* (rel) ± SEM (filled symbols) of **Fl-AspA** (10 µM) and relative viability *RV* ± SEM (empty symbols) in HK cells as a function of the concentration of A) **16F16**, B) **LOC14**, C) **PACMA** and D) **Rutin**.

Table S16. Dependence of cellular uptake of **Fl-AspA** and cell viability in HK cells on the concentration of PDI inhibitors under co-incubation condition.[a]

Entry	I[b]	MIC (μM)[c]	IC_{50} (μM)[d]	*n* (IC_{50})[e]	RV_{50} (μM)[f]	*n* (RV_{50})[g]
1	**16F16**	-	-	-	>50	-
2	**LOC14**	-	-	-	>20	-
3	**PACMA-31**	-	-	-	4 ± 1	1.0 ± 0.2
4	**Rutin**	-	-	-	>500	-

[a]Results from dose-response curves in Figure S43. [b]Inhibitor. [c]Concentration needed to reach 15% inhibition. [d]Concentration needed to reach 50% inhibition. [e]Hill coefficient for inhibition of cellular uptake. [f]Concentration needed to lower relative viability (RV) by 50%. [g]Hill coefficient for cell viability.

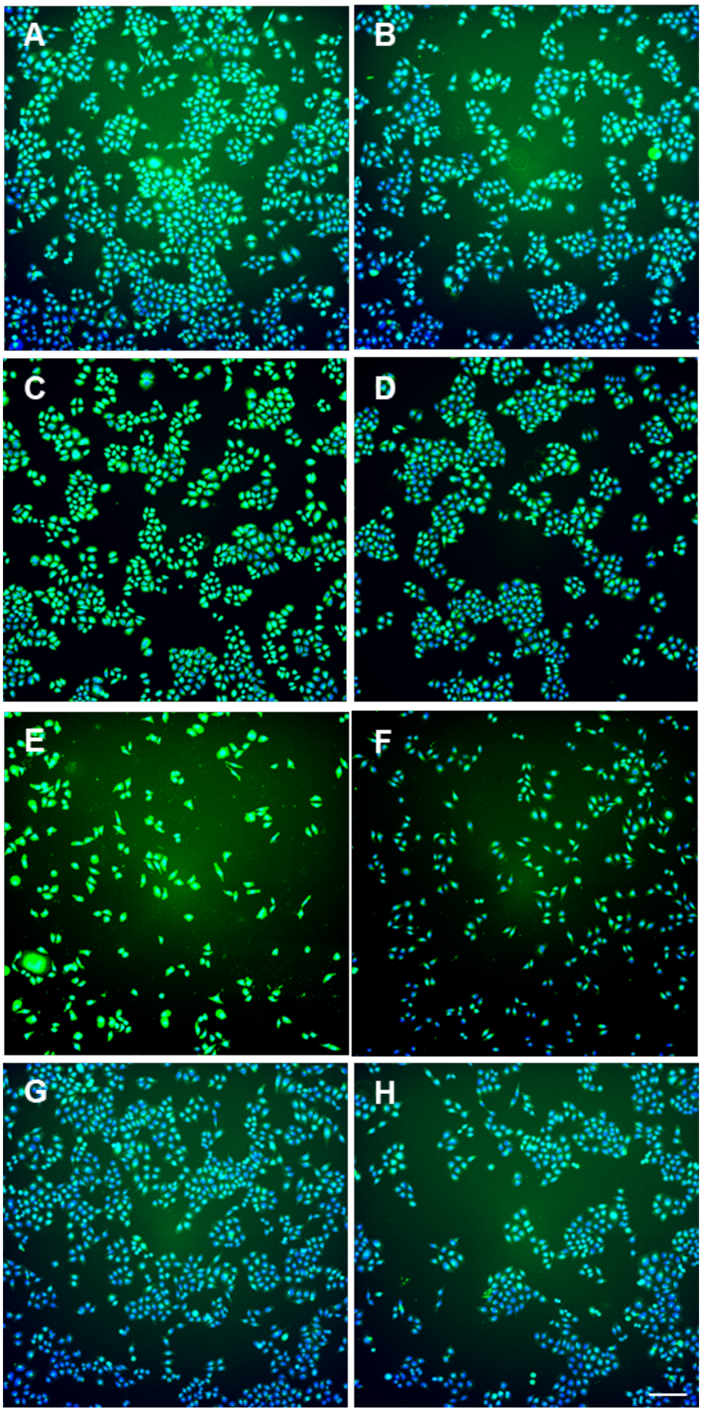

Figure S44. SDCM images (10X, widefield) showing fluorescence intensity (green) of **FI-AspA** (10 µM) A) with or B) without **16F16** (50 µM), C) with or D) without **LOC14** (50 µM), E) with or D) without **PACMA-31** (16 µM) and G) with and H) without **Rutin** (500 µM) (blue: Hoechst 33342, nuclei; scale bar 150 µm). Cell count: 300 cells for 16F16 and PACMA, 1000 cells for all other conditions.

9.2.4. Effects on Fl-CTO Uptake in HK Cells

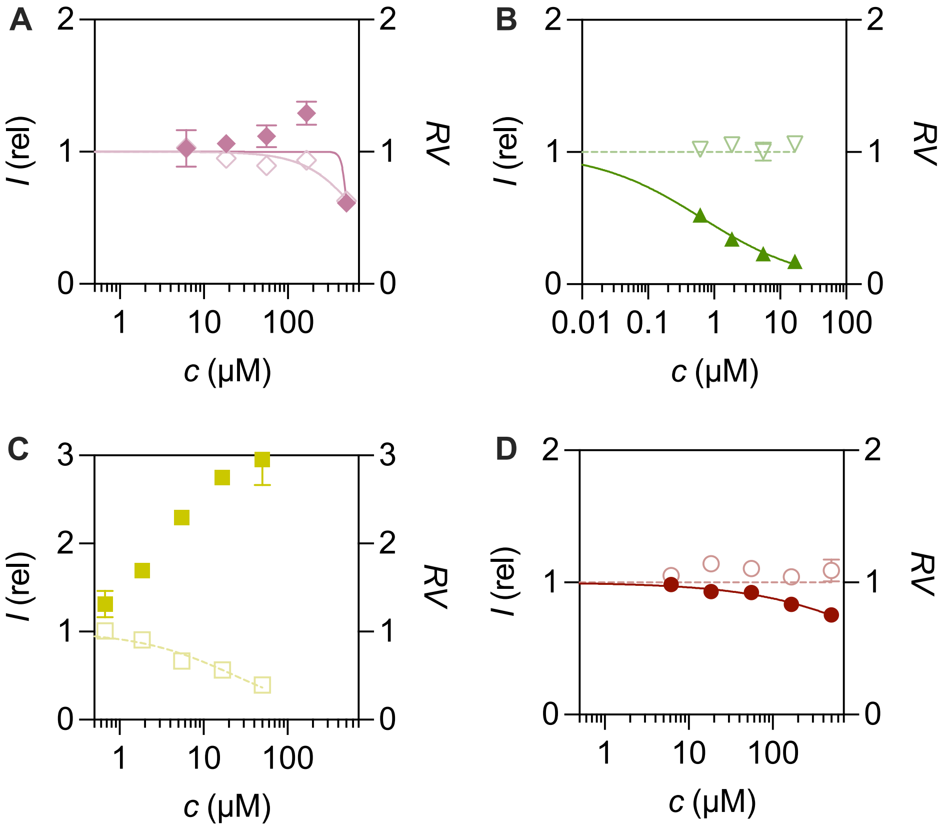

Figure S45. Relative fluorescence intensity *I* (rel) ± SEM (filled symbols) of **Fl-CTO** (5 µM) and relative viability *RV* ± SEM (empty symbols) in HK cells as a function of the concentration of A) **16F16**, B) **LOC14**, C) **PACMA** and D) **Rutin**.

Table S17. Dependence of cellular uptake of **Fl-CTO** and cell viability in HK cells on the concentration of PDI inhibitors under co-incubation condition.[a]

Entry	I[b]	MIC (μM)[c]	IC_{50} (μM)[d]	*n* (IC_{50})[e]	RV_{50} (μM)[f]	*n* (RV_{50})[g]
1	**16F16**	430	(550)	-	>500	-
2	**LOC14**	<0.6	0.7 ± 0.1	0.5 ± 0.1	>50	-
3	**PACMA-31**	-	-	-	25 ± 4	0.7 ± 0.1
4	**Rutin**	150	>>500	-	>500	-

[a]Results from dose-response curves in Figure S45. [b]Inhibitor. [c]Concentration needed to reach 15% inhibition. [d]Concentration needed to reach 50% inhibition. [e]Hill coefficient for inhibition of cellular uptake. [f]Concentration needed to lower relative viability (RV) by 50%. [g]Hill coefficient for cell viability.

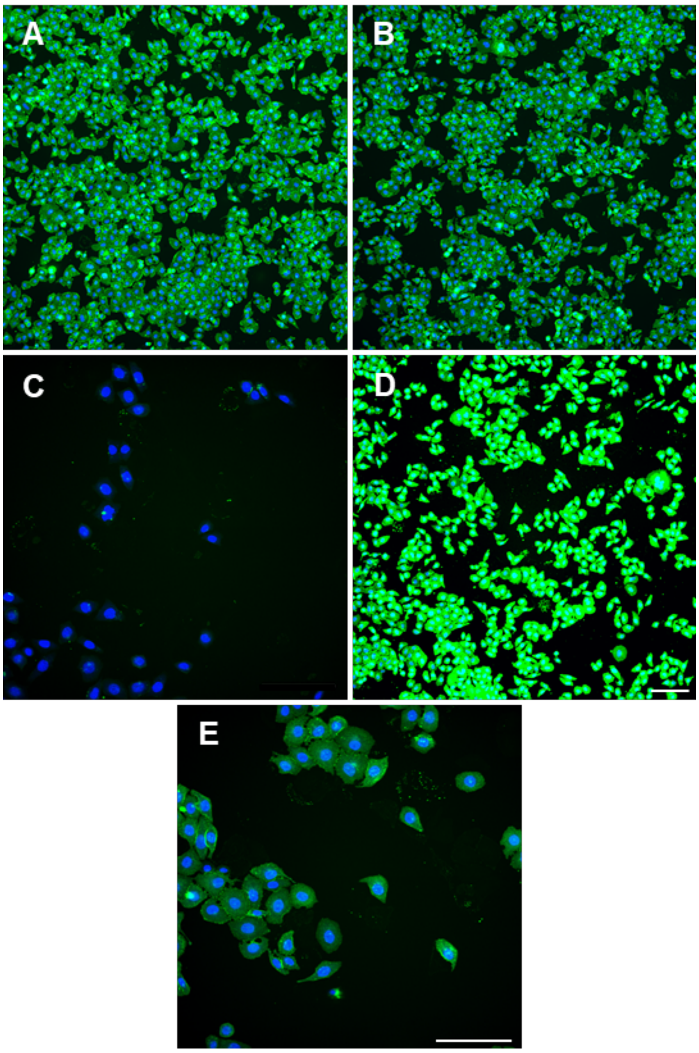

Figure S46. SDCM images (10X, widefield) showing fluorescent intensity (green) of **FI-CTO** (5 µM) A) without any inhibitor, or with B) **16F16** (50 µM), C) **LOC14** (50 µM), D) **PACMA-31** (16 µM) and D) **Rutin** (500 µM) (blue: Hoechst 33342, nuclei; scale bar 150 µm). Cell count: 1000 cells for all conditions.

9.2.5. Effects on Fl-MAC Uptake in HK Cells

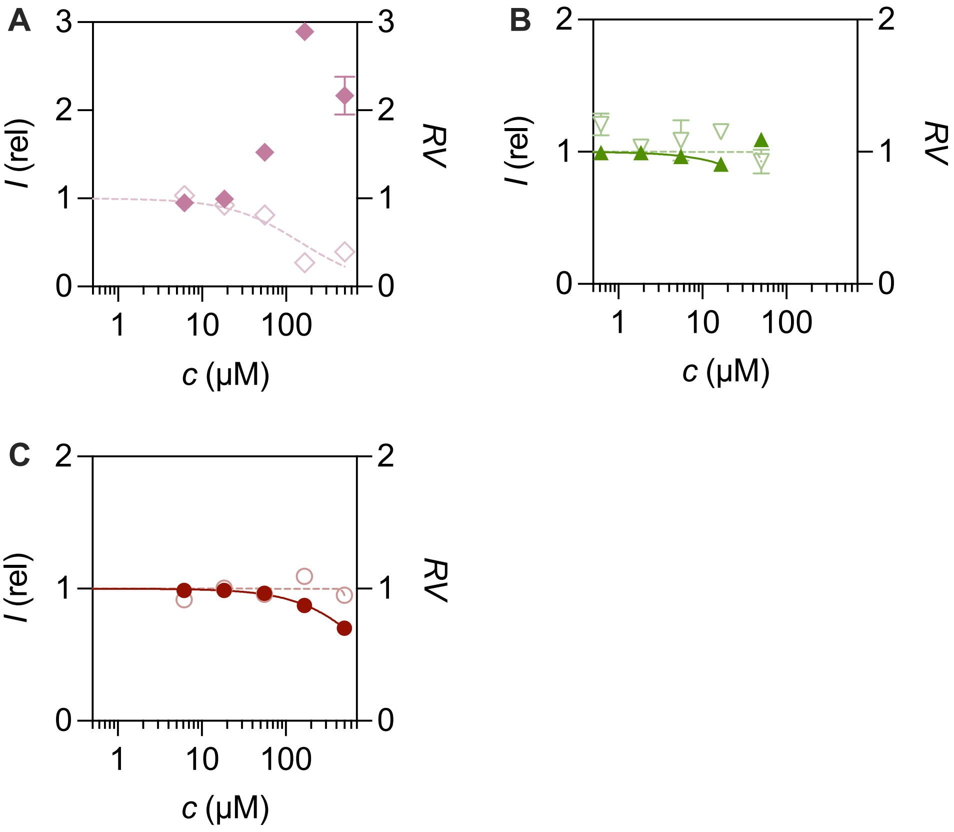

Figure S47. Relative fluorescence intensity I (rel) ± SEM (filled symbols) of **Fl-MAC** (10 μM) and relative viability RV ± SEM (empty symbols) in HK cells as a function of the concentration of A) **16F16**, B) **LOC14** and C) **Rutin**.

Table S18. Dependence of cellular uptake of **Fl-MAC** and cell viability in HK cells on the concentration of PDI inhibitors under co-incubation condition.[a]

Entry	I[b]	MIC (μM)[c]	IC_{50} (μM)[d]	n (IC_{50})[e]	RV_{50} (μM)[f]	n (RV_{50})[g]
1	**16F16**	-	-	-	150 ± 30	1.0 ± 0.3
2	**LOC14**	-	(140)	-	>50	-
3	**Rutin**	210	(1100)	-	>500	-

[a]Results from dose-response curves in Figure S47. [b]Inhibitor. [c]Concentration needed to reach 15% inhibition. [d]Concentration needed to reach 50% inhibition. [e]Hill coefficient for inhibition of cellular uptake. [f]Concentration needed to lower relative viability (RV) by 50%. [g]Hill coefficient for cell viability.

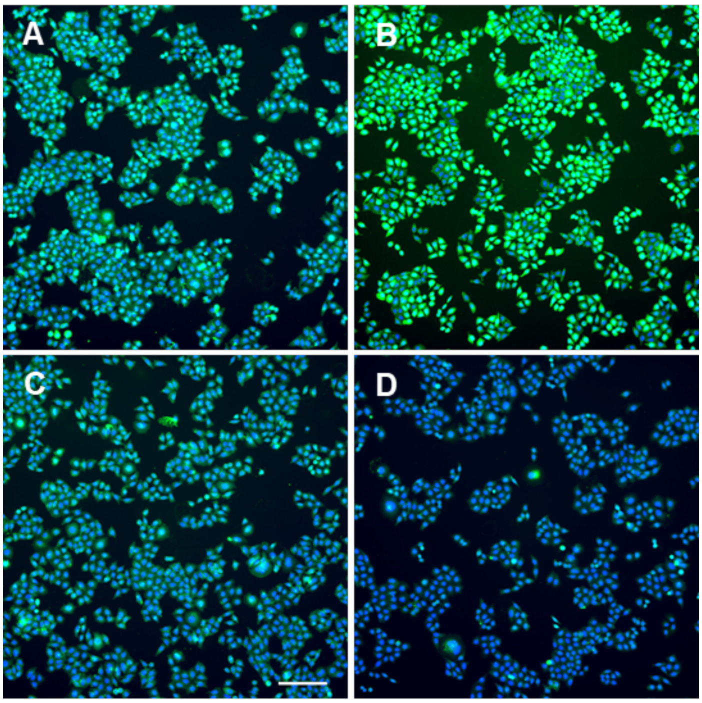

Figure S48. SDCM images (10X, widefield) showing fluorescence intensity (green) of **FI-MAC** (10 µM) A) without any inhibitor, or with B) **16F16** (50 µM), C) **LOC14** (50 µM) and D) **Rutin** (500 µM) (blue: Hoechst 33342, nuclei; scale bar 150 µm). Cell count: 1000 cells for all conditions.

9.2.6. Effects on OPS-Cy5 Uptake in HK Cells

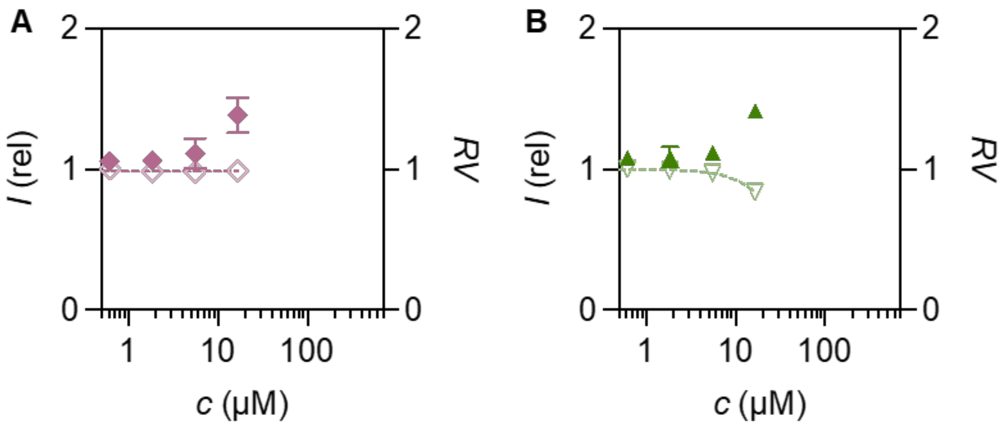

Figure S49. Relative fluorescence intensity *I* (rel) ± SEM (filled symbols) of **OPS-Cy5** (0.5 µM) and relative viability *RV* ± SEM (empty symbols) in HK cells as a function of the concentration of A) **16F16** and B) **LOC14**.

Table S19. Dependence of cellular uptake of **OPS-Cy5** and cell viability in HK cells on the concentration of PDI inhibitors under co-incubation condition.[a]

Entry	I[b]	MIC (µM)[c]	IC_{50} (µM)[d]	*n* (IC_{50})[e]	RV_{50} (µM)[f]	*n* (RV_{50})[g]
1	**16F16**	-	-	-	>16	-
2	**LOC14**	-	-	-	>16	-

[a]Results from dose-response curves in Figure S49. [b]Inhibitor. [c]Concentration needed to reach 15% inhibition. [d]Concentration needed to reach 50% inhibition. [e]Hill coefficient for inhibition of cellular uptake. [f]Concentration needed to lower relative viability (RV) by 50%. [g]Hill coefficient for cell viability.

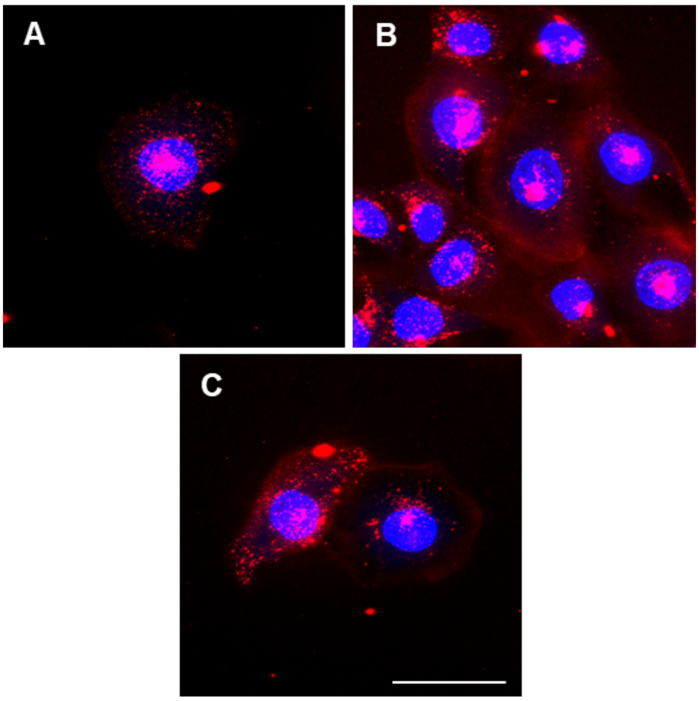

Figure S50. SDCM images (40X WI) showing fluorescence intensity (red) of **OPS-Cy5** (0.5 µM) A) without any inhibitor, or with B) **16F16** (50 µM) and C) **LOC14** (50 µM) (blue: Hoechst 33342, nuclei; scale bar 150 µm). Cell count: 150 cells for all conditions.

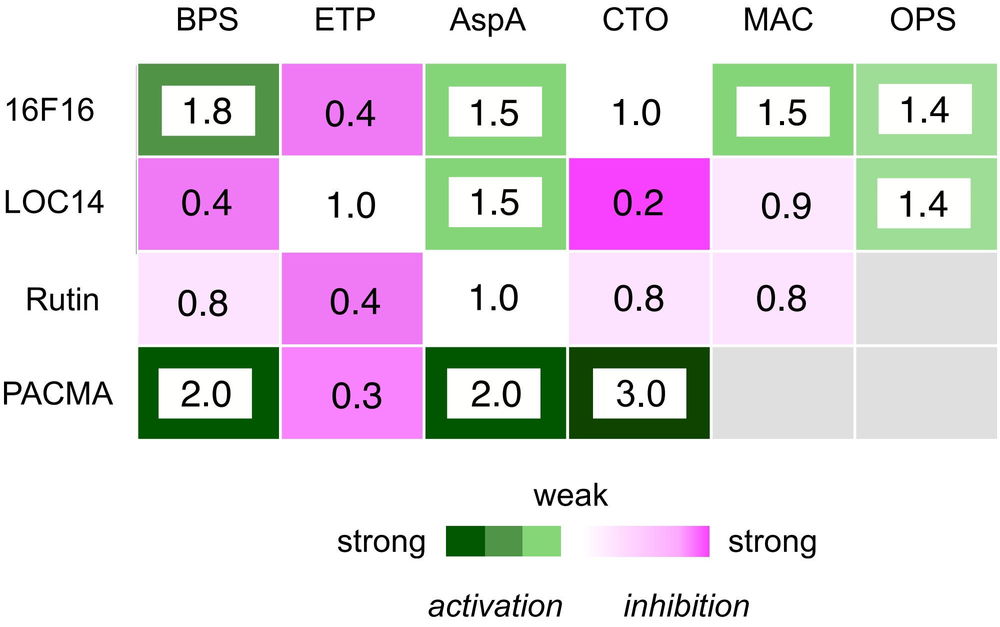

Figure S51. Heatmap for the relative fluorescence *I* (rel) obtained at the highest viable concentration for the inhibition of cellular uptake of different TMU transporters by the alternative inhibitors **16F16**, **LOC14**, **Rutin** and **PACMA-31**.

9.2.7. Fl-BPS Uptake in Different Cell Lines

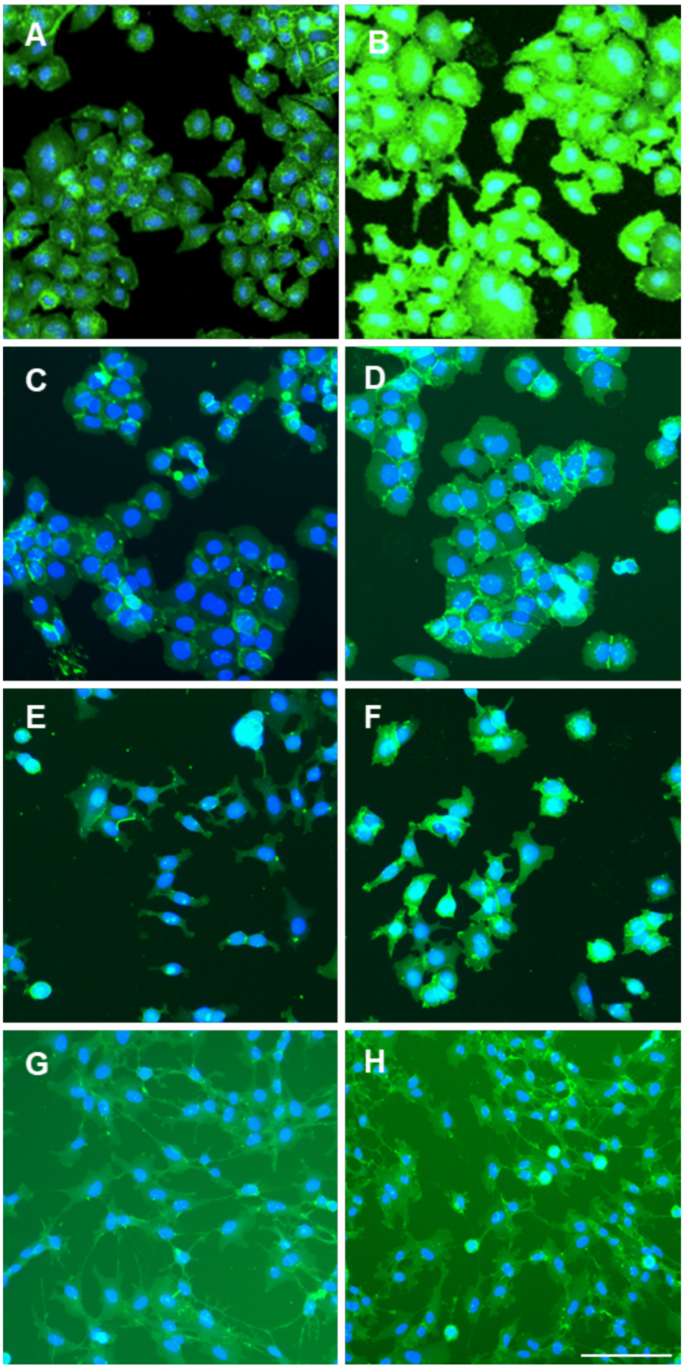

Figure S52. SDCM images (10X, widefield) showing fluorescent intensity (green) of **FI-BPS** (5 µM) in non-treated (left column) and **16F16** treated (50 µM, co-incubation, right column) (A, B) HK, (C, D) A431, (E, F) MCF-7 and (G, H) RPE-1 cell lines (blue: Hoechst 33342, nuclei; scale bar: 100 µm).

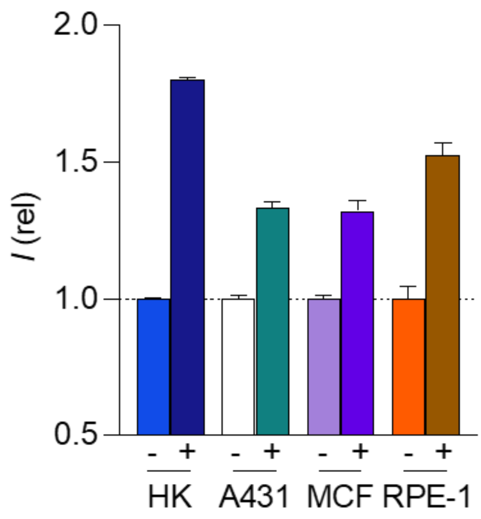

Figure S53. Relative fluorescence intensity *I* (rel) ± SEM of **FI-BPS** (5 µM) in different cell lines without (-) and with **16F16** treatment (50 µM, co-incubation, +).

9.3. Inhibitor Screening in the Presence of PDI Inhibitors

Experimental procedure was as described in section 6 for the co-incubation method. PDI inhibitor was added 30 min before the addition of TMU inhibitors (without washing) at a fixed concentration of 25 µM and 50 µM for LOC14 (**AI2**) and 16F16 (**AI1**), respectively. Duplicates were performed for each condition.

9.3.1. Fl-BPS Transporter

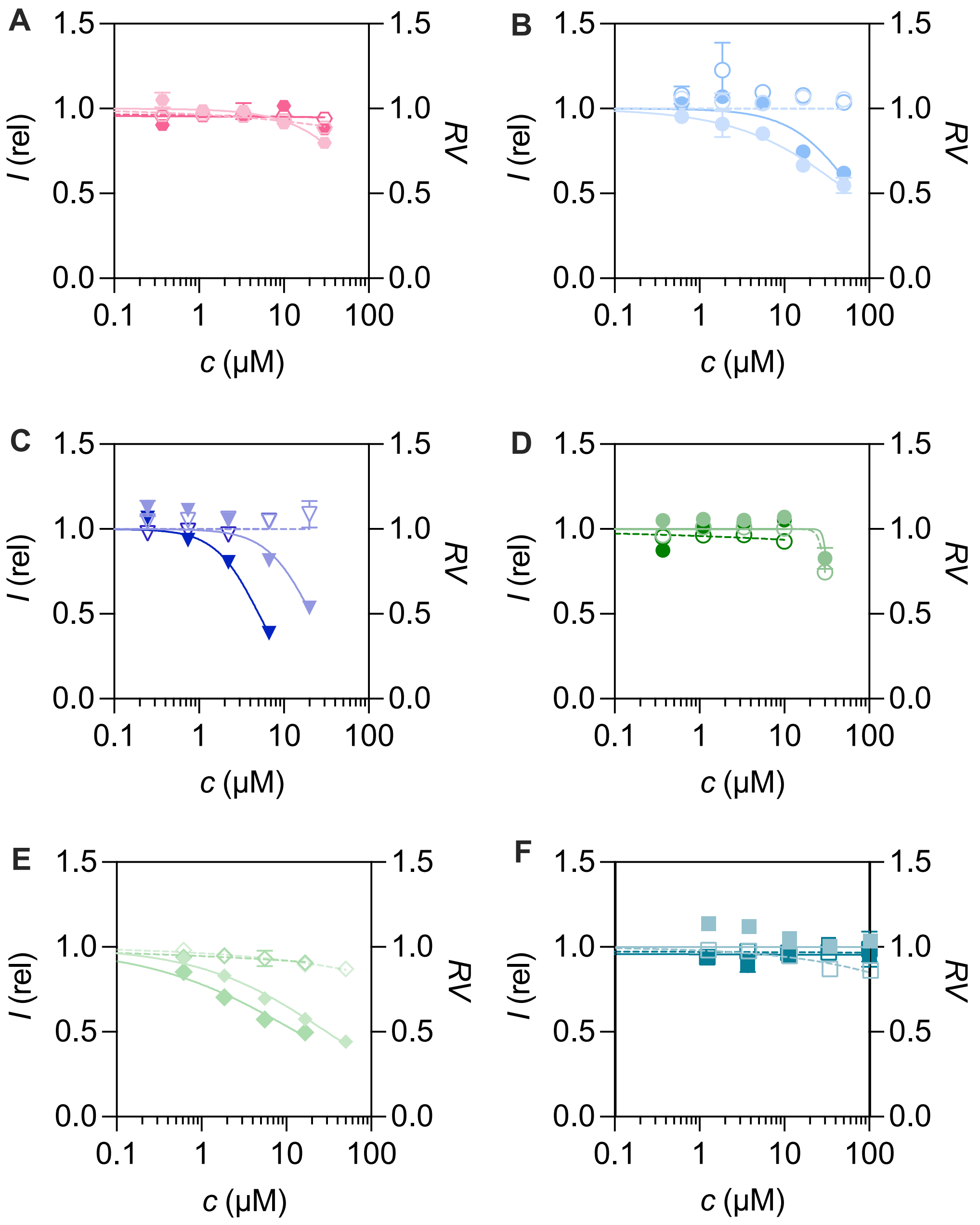

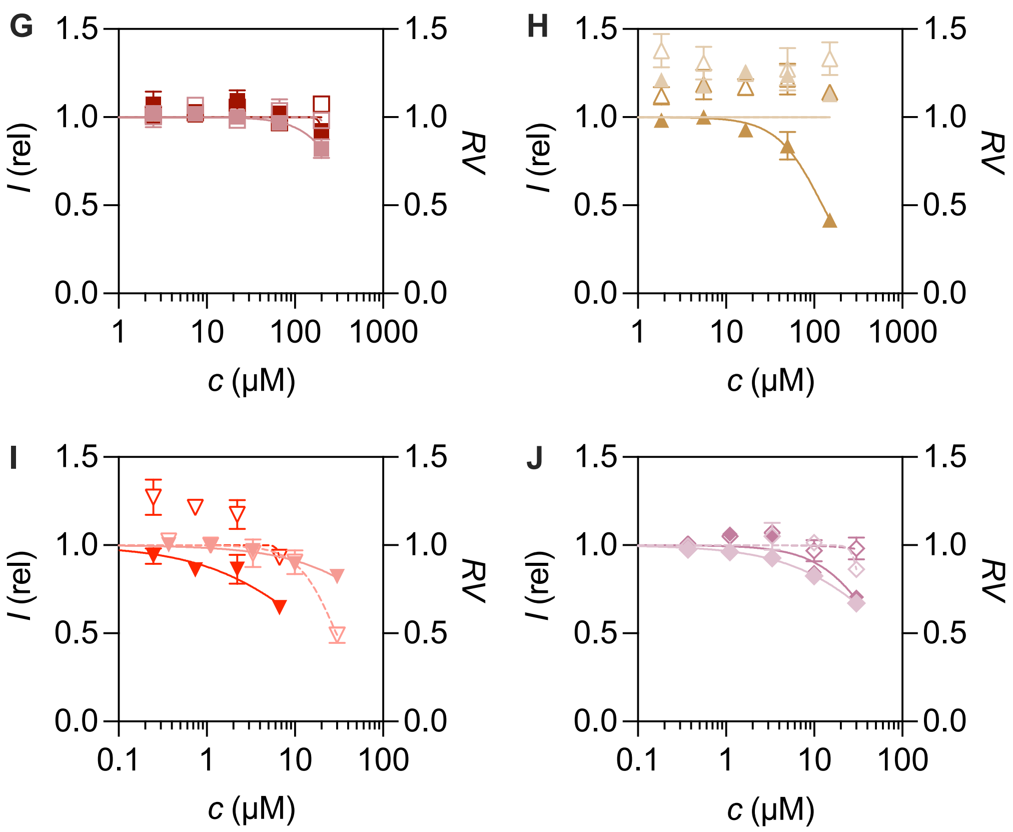

Figure S54. Relative fluorescence intensity *I* (rel) ± SEM (filled symbols) of **Fl-BPS** (5 µM) in HK cells and relative viability *RV* (rel) ± SEM (empty symbols) with or without **16F16** (50 µM; lighter or darker color, respectively) as a function of the concentration of A) **EBX**, B) **EBS**, C) **BiC**, D) **SS**, E) **dMAC**, F) **MAC**, G) **CTO**, H) **AspA**, I) **ETP** and J) **BPS**.

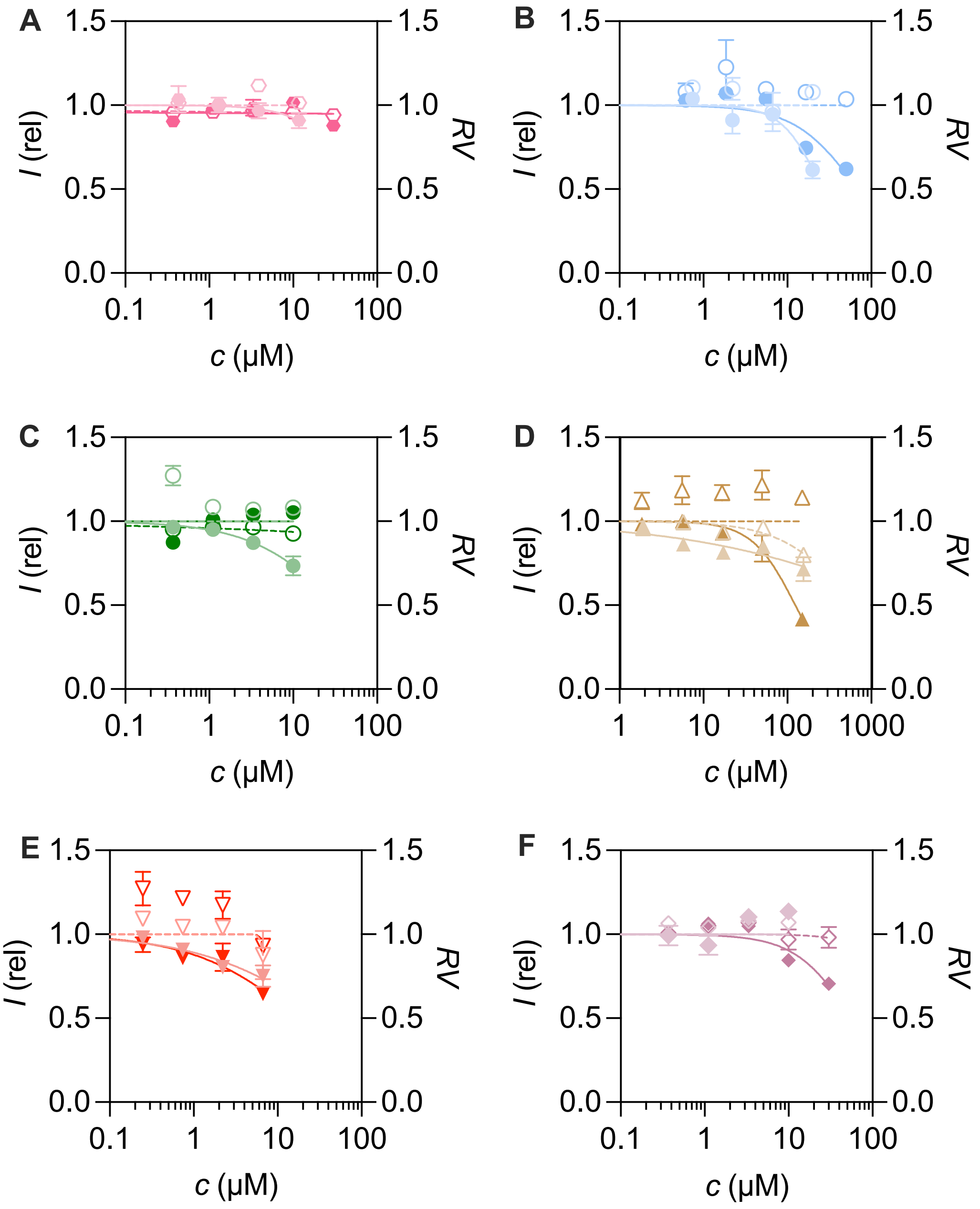

Figure S55. Relative fluorescence intensity *I* (rel) ± SEM (filled symbols) of **Fl-BPS** (5 μM) in HK cells and relative viability *RV* (reI) ± SEM (empty symbols) with or without **LOC14** (25 μM; lighter or darker color, respectively) as a function of the concentration of A) **EBX**, B) **EBS**, C) **SS**, D) **AspA**, E) **ETP** and F) **BPS**.

Table S20. Dependence of cellular uptake of **Fl-BPS** (5 μM) and cell viability in PDI inhibitor treated HK cells on the concentration of CAX inhibitors under co-incubation condition. (Part I)[a]

Entry	I[b]	AI[c]	MIC (μM)[d]	IC_{50} (μM)[e]	n (IC_{50})[f]	RV_{50} (μM)[g]	n (RV_{50})[h]
1	**EBX**	-	-	-	-	>30	-
2		**16F16**	20	(100)	-	>30	-
3		**LOC14**	-	(60)	-	>35	-
4	**EBS**	-	15	(70)	-	>50	-
5		**16F16**	4	(60)	-	>50	-
6		**LOC14**	10	(25)	-	>20	-
7	**BiC**	-	2	5.0 ± 0.4	2.0 ± 0.2	>7	-
8		**16F16**	7	20 ± 4	2 ± 1	>20	-
9	**SS**	-	-	-	-	>10	-
10		**16F16**	-	(35)	-	>30	-
11		**LOC14**	4	(35)	-	>10	-
12	**dMAC**	-	<0.6	15 ± 2	0.5 ± 0.1	>17	-
13		**16F16**	1	30 ± 4	0.6 ± 0.1	>50	-

[a]Results from dose-response curves in Figure S55. [b]Inhibitor. [c]Alternative inhibitor. [d]Concentration needed to reach 15% inhibition. [e]Concentration needed to reach 50% inhibition. [f]Hill coefficient for inhibition of cellular uptake. [g]Concentration needed to lower relative viability (RV) by 50%. [h]Hill coefficient for cell viability.

Table S21. Dependence of cellular uptake of **Fl-BPS** (5 μM) and cell viability in PDI inhibitor treated HK cells on the concentration of CAX inhibitors under co-incubation condition. (Part II)[a]

Entry	I[b]	AI[c]	MIC (μM)[d]	IC_{50} (μM)[e]	n (IC_{50})[f]	RV_{50} (μM)[g]	n (RV_{50})[h]
1	**MAC**	-	-	-	-	>100	-
2		**16F16**	-	-	-	>100	-
3	**CTO**	-	-	-	-	>200	-
4		**16F16**	170	(450)	-	>200	-
5	**AspA**	-	45	125 ± 10	2.0 ± 0.2	>150	-
6		**16F16**	-	-	-	>150	-
7		**LOC14**	20	>>100	-	>150	-
8	**ETP**	-	1	(20)	-	>7	-
9		**16F16**	20	(200)	-	30 ± 2	2.0 ± 0.3
10		**LOC14**	2	(40)	-	>7	-
11	**BPS**	-	15	(50)	-	>30	-
12		**16F16**	8	(75)	-	>30	-
13		**LOC14**	-	-	-	>10	-

[a]Results from dose-response curves in Figure S55. [b]Inhibitor. [c]Alternative inhibitor. [d]Concentration needed to reach 15% inhibition. [e]Concentration needed to reach 50% inhibition. [f]Hill coefficient for inhibition of cellular uptake. [g]Concentration needed to lower relative viability (RV) by 50%. [h]Hill coefficient for cell viability.

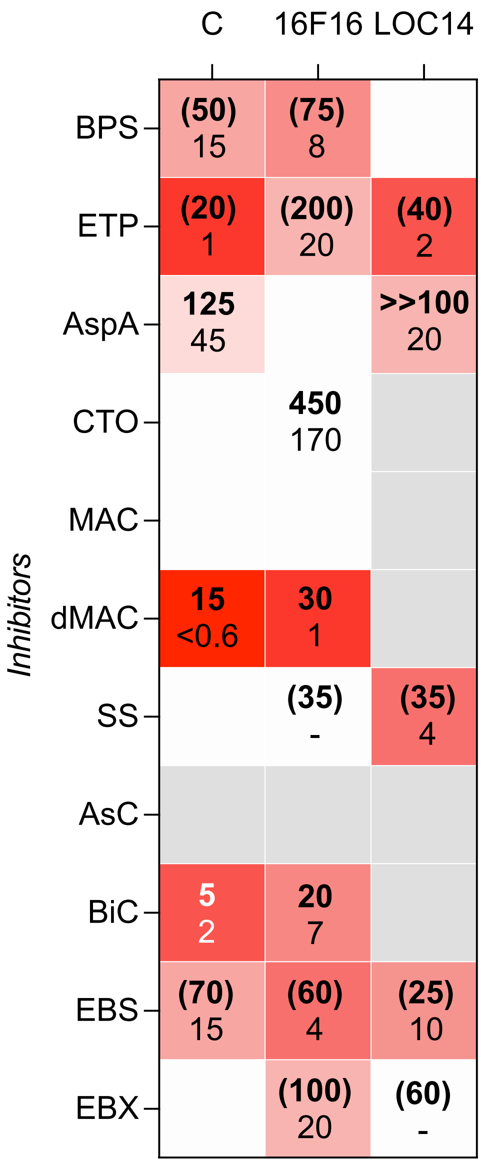

Figure S56. Heatmap for the MIC of candidates to inhibit cellular uptake of **FI-BPS** in HeLa Kyoto cells treated with PDI inhibitors.

9.3.2. Fl-ETP Transporter

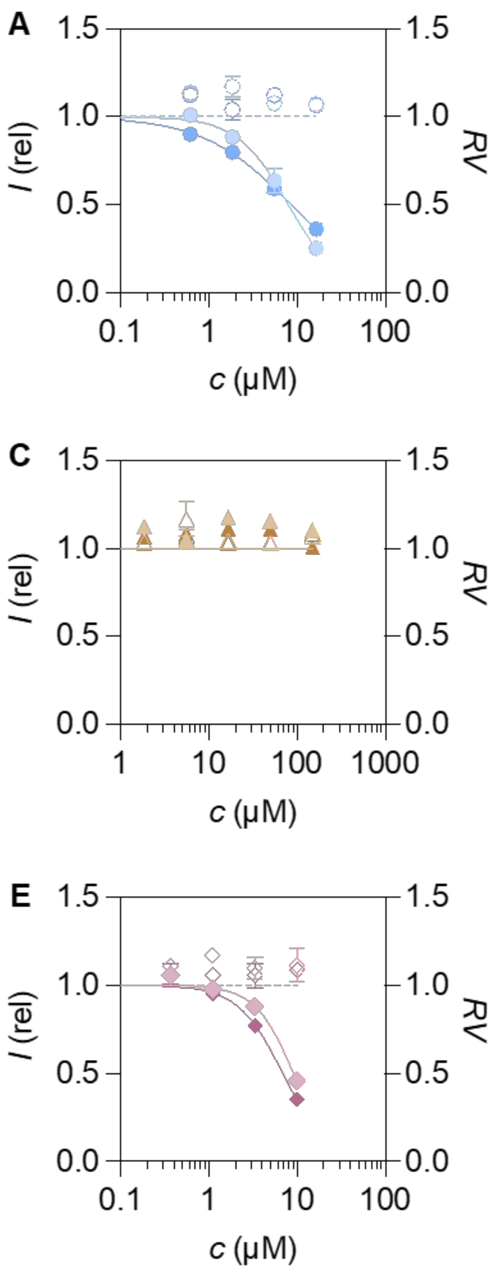

Figure S57. Relative fluorescence intensity *I* (rel) ± SEM (filled symbols) of **Fl-ETP** (10 µM) in HK cells and relative viability *RV* (rel) ± SEM (empty symbols) as a function of the concentration of A) **EBS**, B) **SS**, C) **AspA**, D) **ETP** and F) **BPS**. Dark color: control, lighter color: **16F16** (50 µM).

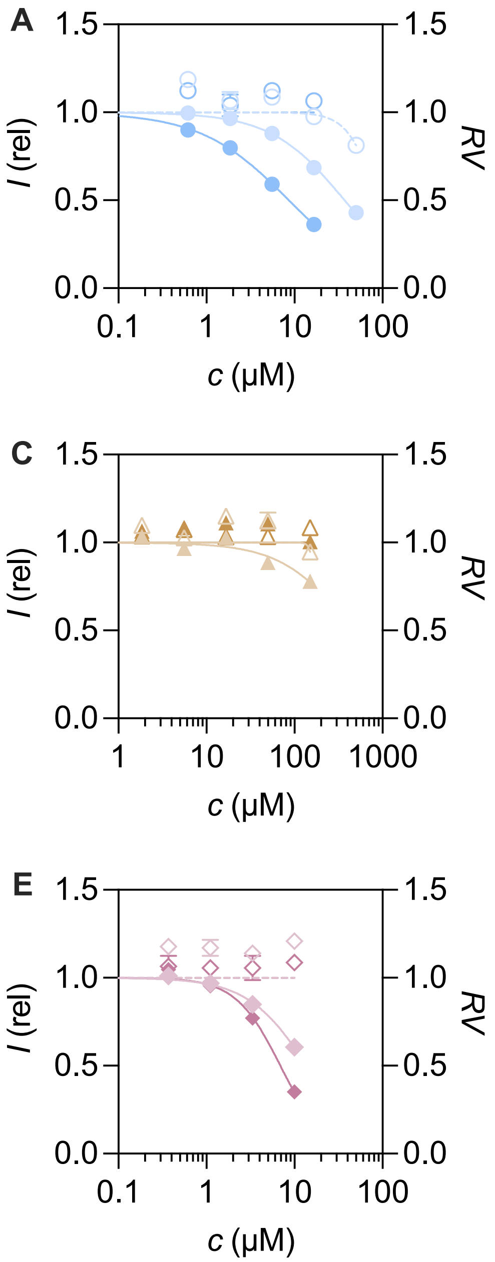

Figure S58. Relative fluorescence intensity *I* (rel) ± SEM (filled symbols) of **Fl-ETP** (10 µM) in HK cells and relative viability *RV* (rel) ± SEM (empty symbols) with or without **LOC14** (25 µM; lighter or darker color, respectively) as a function of the concentration of A) **EBS**, B) **SS**, C) **AspA**, D) **ETP** and E) **BPS**.

Table S22. Dependence of cellular uptake of **Fl-ETP** (10 μM) and cell viability in PDI inhibitor treated HK cells on the concentration of CAX inhibitors under co-incubation condition.[a]

Entry	I[b]	AI[c]	MIC (μM)[d]	IC_{50} (μM)[e]	*n* (IC_{50})[f]	RV_{50} (μM)[g]	*n* (RV_{50})[h]
1	**EBS**	-	1	8.0 ± 0.5	0.90 ± 0.05	>16	-
2		**16F16**	3	8.0 ± 0.7	1.0 ± 0.2	>16	-
3		**LOC14**	7	40 ± 2	1.0 ± 0.1	>50	-
4	**SS**	-	-[g]	-	-	>10	-
5		**16F16**	-[g]	-	-	>20	-
6		**LOC14**	-[g]	>30	-	>30	-
7	**AspA**	-	-	-	-	>150	-
8		**16F16**	-	-	-	>150	-
9		**LOC14**	100	>150	-	>150	-
10	**ETP**	-	1	5.0 ± 0.5	0.9 ± 0.1	>20	-
11		**16F16**	1	(10)	-	>7	-
12		**LOC14**	1	(10)	-	>7	-
13	**BPS**	-	3	7.0 ± 0.4	1.7 ± 0.2	>10	-
14		**16F16**	4	10.0 ± 0.4	2.0 ± 0.2	>10	-
15		**LOC14**	4	(15 ± 1)	1.0 ± 0.1	>10	-

[a]Results from dose-response curves in Figure S57. [b]Inhibitor. [c]Alternative inhibitor. [d]Concentration needed to reach 15% inhibition. [e]Concentration needed to reach 50% inhibition. [f]Hill coefficient for inhibition of cellular uptake. [g]Concentration needed to lower relative viability (RV) by 50%. [h]Hill coefficient for cell viability.

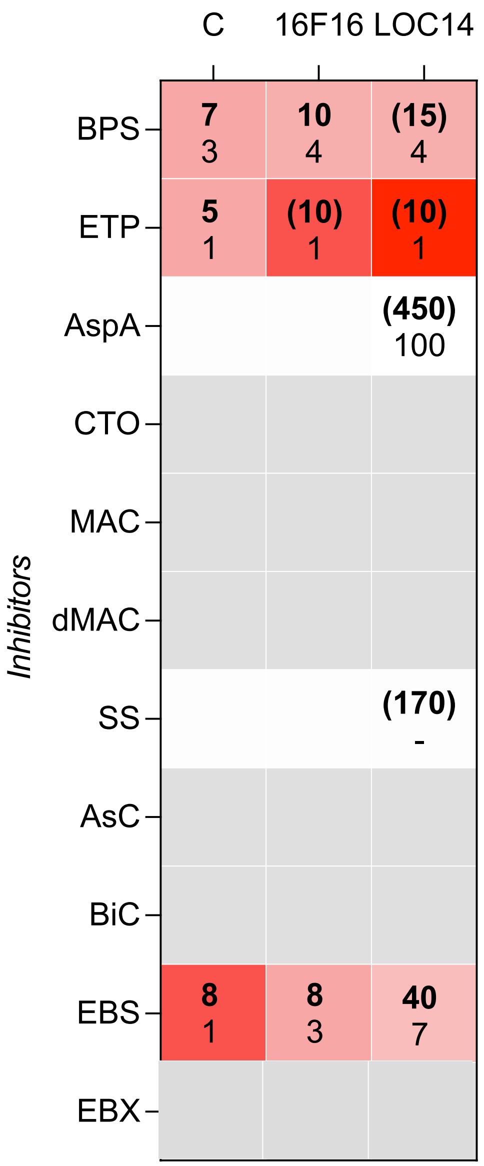

Figure S59. Heatmap for the MIC of candidates to inhibit cellular uptake of **FI-ETP** in HeLa Kyoto cells treated with PDI inhibitors.

10. Co-Localization of OPS-Cy5 and Transferrin CF488A Conjugate in HK Cells

HK cells were prepared as described in section 4. The medium was removed and cells were washed with PBS (3 × 3 mL/well) and fresh FDMEM (4 × 100 µL/well) using a plate washer (Biotek EL406®), keeping a final volume of 100 µL/well. Freshly prepared solution of **OPS-Cy5** in FDMEM (6X final concentration) and transferrin CF488A (**Tf CF488A**, BIOTIUM INC.) (6X final concentration) were added simultaneously to HK cells (25 µL each solution), resulting in a final concentration of 0.5 µM for **OPS-Cy5** and 40 µg/mL for **Tf CF488A**, and a final volume of 150 µL/well. The cells were incubated for indicated duration at 37 °C with 5% CO_2. The cells were washed with PBS (9 × 3 mL/well) and immediately fixed with a solution of PFA 3% (70 µL/well) for 15 min at rt. The cells were washed with PBS (9 × 3 mL/well) and treated with a solution of Hoechst 33342 (50 µg/mL, 50µL/well) in PBS for 10 min at rt. The excess of the dye was removed with additional washing with PBS (9 × 3 mL/well). The distribution of fluorescence stained cells was analyzed on a IXM-C automated microscope acquiring 30 images per well using a 60× objective lens WI with 3 channels, blue for Hoechst 33342 (377/50 nm excitation filter; 477/60 nm emission filter), green for **Tf CF488A** (475/34 nm excitation filter; 536/40 nm emission filter) and red for **OPS-Cy5** (excitation filter: 620/50 nm; emission filter: 690/50 nm).

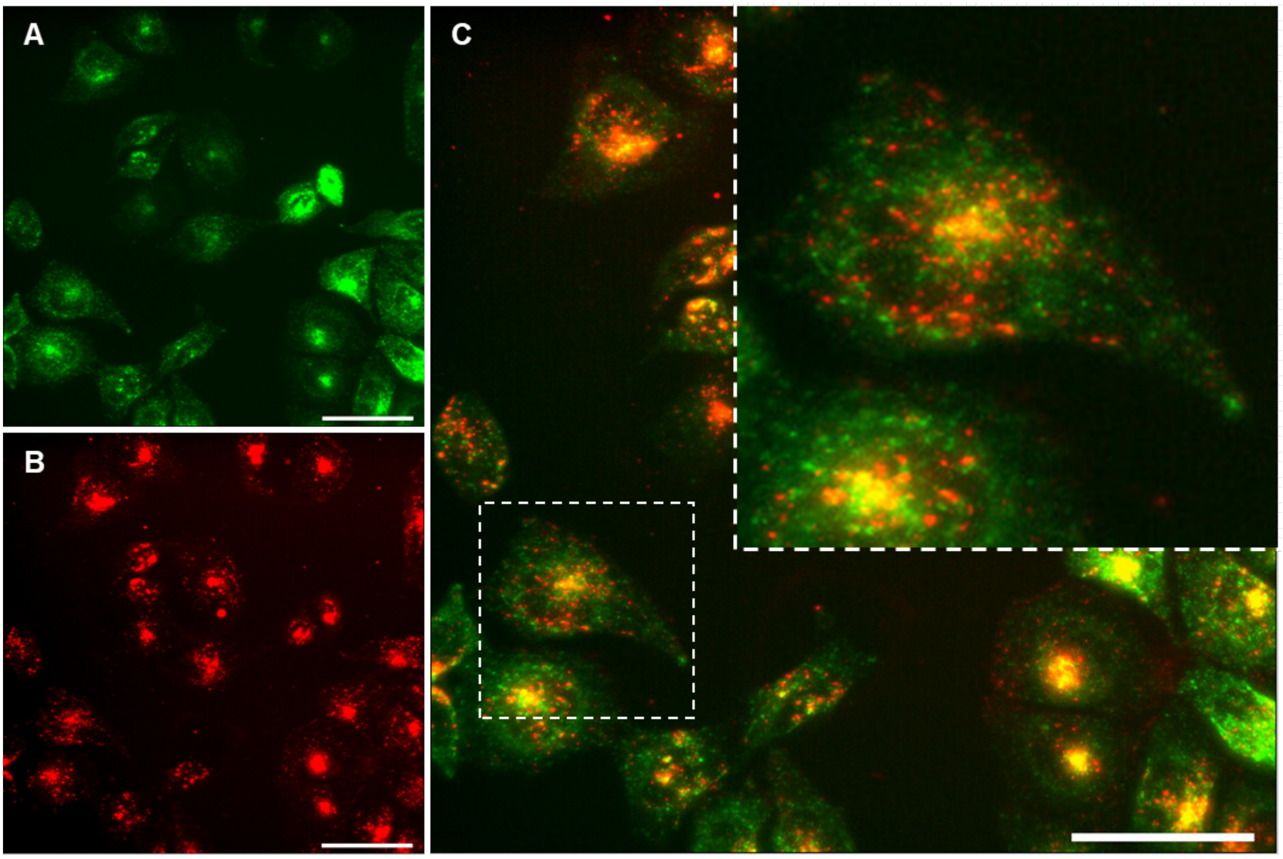

Figure S60. SDCM images (60X WI) of A) **Tf CF488A** (40 µg.ml^{-1}, green channel), B) **OPS-Cy5** (500 nM, red channel), and C) both (merged) showing the co-localized uptake (clatherin mediated endocytosis) in HK cells after 60 minutes of incubation.

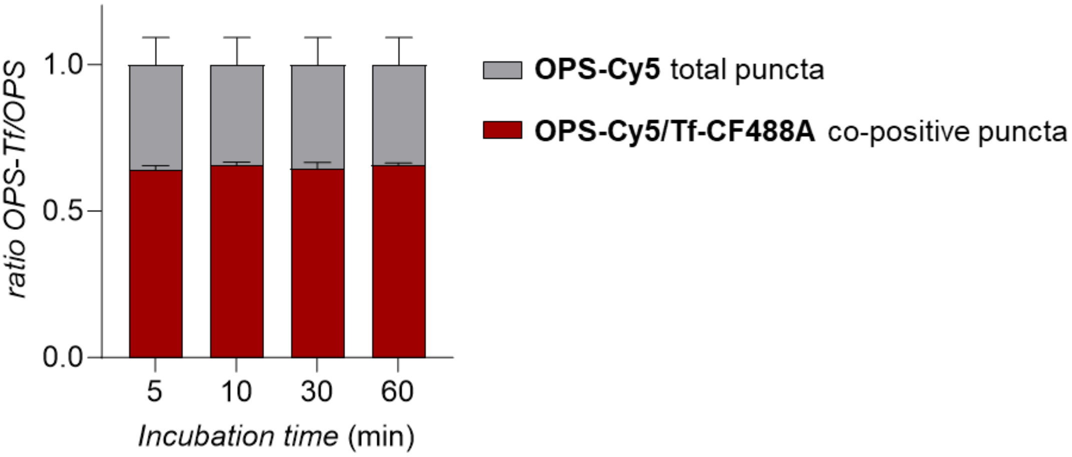

Figure S61. D) Data showing the ratio of co-localized dots of **Tf CF488A** with **OPS-Cy5** over the total amount of **OPS-Cy5** dots after 1 hour of co-incubation.

11. Data Analysis of OPS-Cy5 and Transferrin-CF448A Co-Localization

To determine the ratio of **OPS-Cy5/Tf-CF488A** positive puncta over the total amount of **OPS-Cy5** puncta, the following data analysis protocol was used: as a first step, the nuclei and the cell body were segmented using the blue channel images (Hoechst 333442). A logical operation was performed to build the cell mask form the precited two objects. (Figure S62).

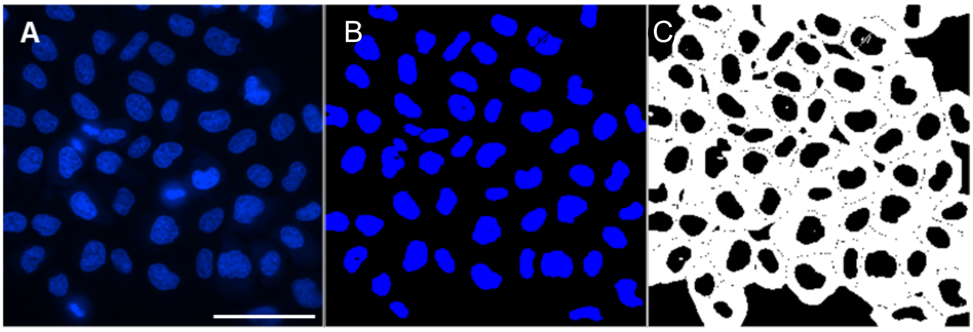

Figure S62. A) SDCM image of the blue channel (Hoechst 33342); B) nuclei mask and C) body cell mask. (scale bar 50 µm).

To count the amount of **Tf-CF488A** dots (green channel), a *Top-hat* transformation was applied to both channels to first detect the puncta. Then, the puncta were filtered out by their size (between 0.3 – 5 µm) and brightness (300 above the background) (Figure S63).

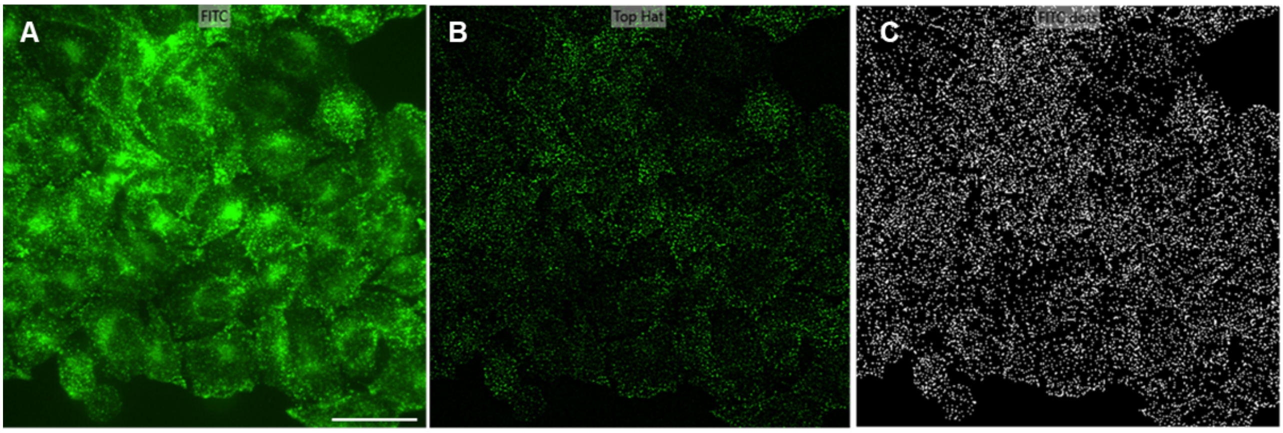

Figure S63. SDCM images of A) the green channel with **Tf-CF488A**, B) *Top-hat* transformation and C) punctum mask. Scale bar 50 µm.

The same procedure was applied to count **OPS-Cy5** dots in the red channel (Figures S64).

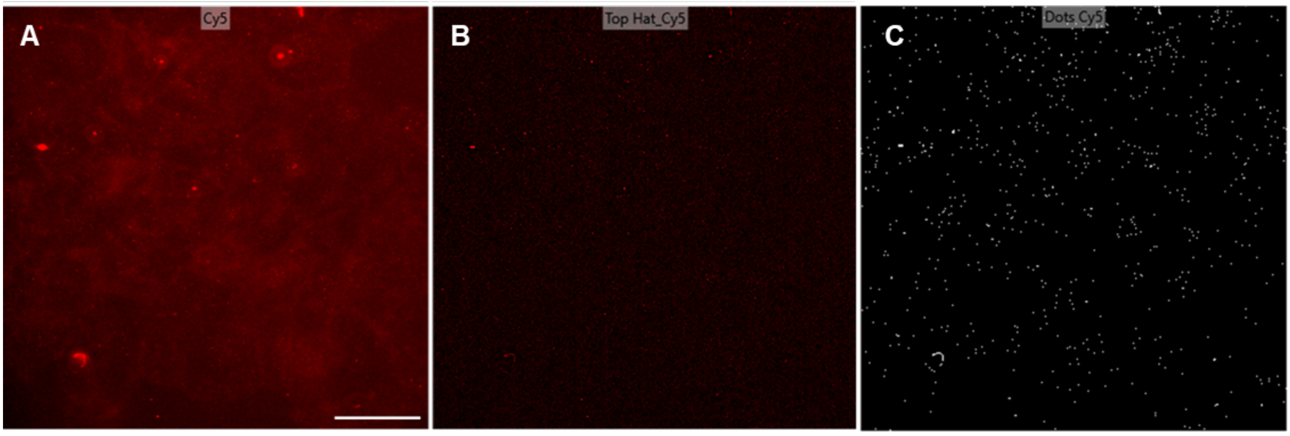

Figure S64. SDCM images of A) the green channel with **Tf-CF488A**, B) *Top-hat* transformation and C) punctum mask. Scale bar 50 µm.

A final co-positive mask was applied to determine the ratio of co-localization: both previous masks for **Tf-CF488A** and **OPS-Cy5** (Figure S61 and S62 respectively) were overlapped, and the puncta that overlap or touch one another are highlighted in yellow (Figure S65). The count of yellow dots over the count of red dots gave the ratio of co-localization.

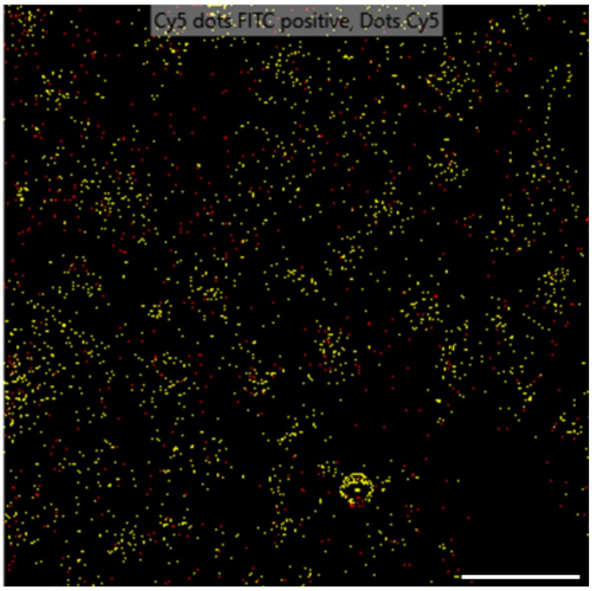

Figure S65. Final mask: yellow – co-positive dots; red – **OPS-Cy5** dots. Scale bar 50 µm.

12. Supporting References

(S1) Shybeka, I.; Maynard, J. R. J.; Saidjalolov, S.; Moreau, D.; Sakai, N.; Matile, S. Dynamic Covalent Michael Acceptors to Penetrate Cells: Thiol-Mediated Uptake with Tetrel-Centered Exchange Cascades, Assisted by Halogen-Bonding Switches. *Angew. Chem. Int. Ed.* **2022**, *61*, e202213433.

(S2) Lim, B.; Cheng, Y.; Kato, T.; Pham, A.-T.; Le Du, E.; Mishra, A. K.; Grinhagena, E.; Moreau, D.; Sakai, N.; Waser, J.; Matile, S. Inhibition of Thiol-Mediated Uptake with Irreversible Covalent Inhibitors. *Helv. Chim. Acta* **2021**, *104*, e2100085.

(S3) Jin, W. B.; Xu, C.; Cheng, Q.; Qi, X. L.; Gao, W.; Zheng, Z.; Chan, E. W. C.; Leung, Y.-C.; Chan, T. H.; Wong, K.-Y.; Chen, S.; Chan, K.-F. Investigation of Synergistic Antimicrobial Effects of the Drug Combinations of Meropenem and 1,2-Benzisoselenazol-3(2H)-One Derivatives on Carbapenem-Resistant Enterobacteriaceae Producing NDM-1. *Eur. J. Med. Chem.* **2018**, *155*, 285–302.

(S4) Lim, B.; Kato, T.; Besnard, C.; Poblador Bahamonde, A. I.; Sakai, N.; Matile, S. PnictogenCentered Cascade Exchangers for Thiol-Mediated Uptake: As(III), Sb(III) and Bi(III) Expanded Cyclic Disulfides as Inhibitors of Cytosolic Delivery and Viral Entry. *JACS Au* **2022**, *2*, 1105-1114.

(S5) Gu, J.; Xiao, B-X.; Chen, Y-R.; Li, Q-Z.; Ouyang, Q.; Du, W.; Chen, Y-C. Interrupted Morita-Baylis-Hillman-Type Reaction of α-Substituted Activated Olefins. *Org. Lett.* **2018**, *20*, 2088–2091.

(S6) Kato, T.; Lim, B.; Cheng, Y.; Pham, A.-T.; Maynard, J.; Moreau, D.; Poblador-Bahamonde, A. I.; Sakai, N.; Matile, S. Cyclic Thiosulfonates for Thiol-Mediated Uptake: Cascade Exchangers, Transporters, Inhibitors. *JACS Au* **2022**, *2*, 839–852.

(S7) Gasparini, G.; Sargsyan, G.; Bang, E.-K.; Sakai, N.; Matile, S. Ring Tension Applied to Thiol-Mediated Cellular Uptake. *Angew. Chem. Int. Ed.* **2015**, *54*, 7328–7331.

(S8) Cheng, Y.; Pham, A.-T.; Kato, T.; Lim, B.; Moreau, D.; López-Andarias, J.; Zong, L.; Sakai, N.; Matile, S. Inhibitors of Thiol-Mediated Uptake. *Chem. Sci.* **2021**, *12*, 626–631.

(S9) Cheng, Y.; Zong, L.; López-Andarias, J.; Bartolami, E.; Okamoto, Y.; Ward, T. R.; Sakai, N.; Matile, S. Cell-Penetrating Dynamic-Covalent Benzopolysulfane Networks. *Angew. Chem. Int. Ed.* **2019**, *58*, 9522–9526.

(S10) Zong, L.; Bartolami, E.; Abegg, D.; Adibekian, A.; Sakai, N.; Matile, S. Epidithiodiketopiperazines: Strain-Promoted Thiol-Mediated Cellular Uptake at the Highest Tension. *ACS Cent. Sci.* **2017**, *3*, 449–453.

(S11) Laurent, Q.; Martinent, R.; Moreau, D.; Winssinger, N.; Sakai, N.; Matile, S. Oligonucleotide Phosphorothioates Enter Cells by Thiol-Mediated Uptake. *Angew. Chem. Int. Ed.* **2021**, *60*, 19102–19106.

The original data can be found at: https://doi.org/10.5281/zenodo.10675775
